# Supplementary figures and images for: Intrinsic and extrinsic motivations governing prey choice by hunters in a post-war African forest-savannah macromosaic
Source: PLoS One. 2021 Dec 20;16(12):e0261198. doi: 10.1371/journal.pone.0261198 (PMC8687528; doi:10.1371/journal.pone.0261198)

Hunting motivation

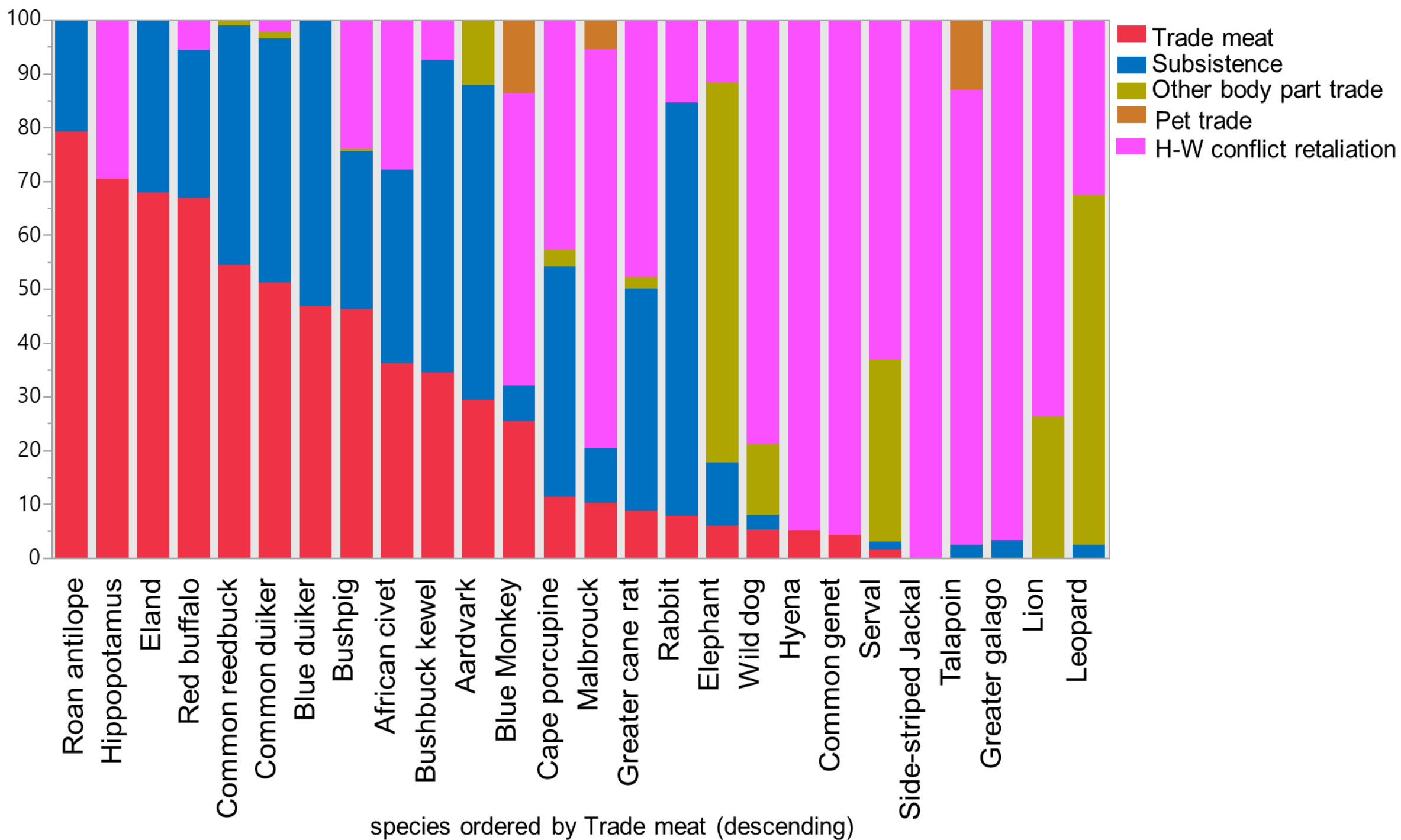

Supplement: S1 Fig — Report rates were calculate from the number of hunters reporting capturing each species for each motivation. Species are denoted by their English names and ordered left to right in accordance to the frequency of meat trade as a motivation for capturing each species. (PDF) [file pone.0261198.s001.pdf]

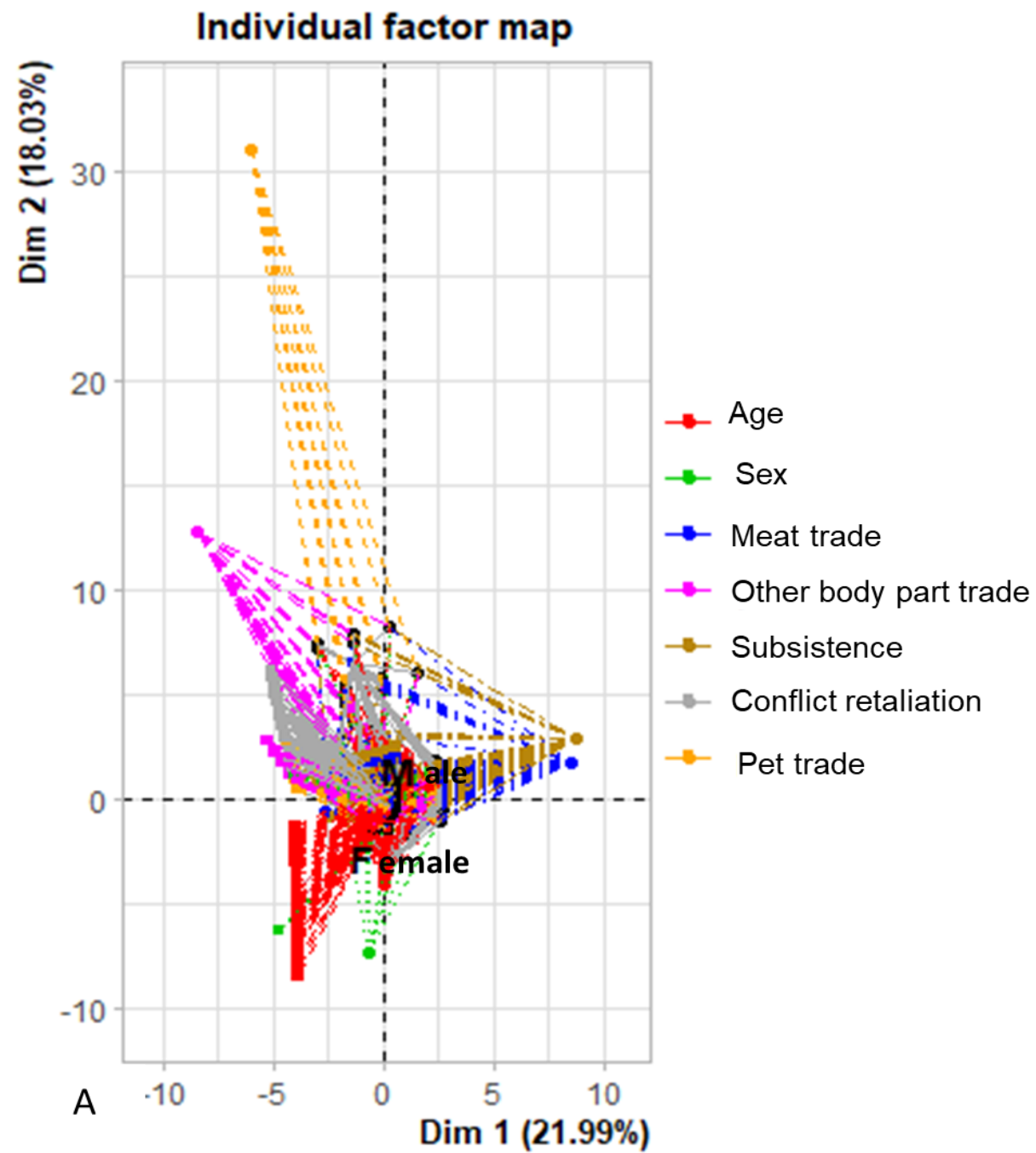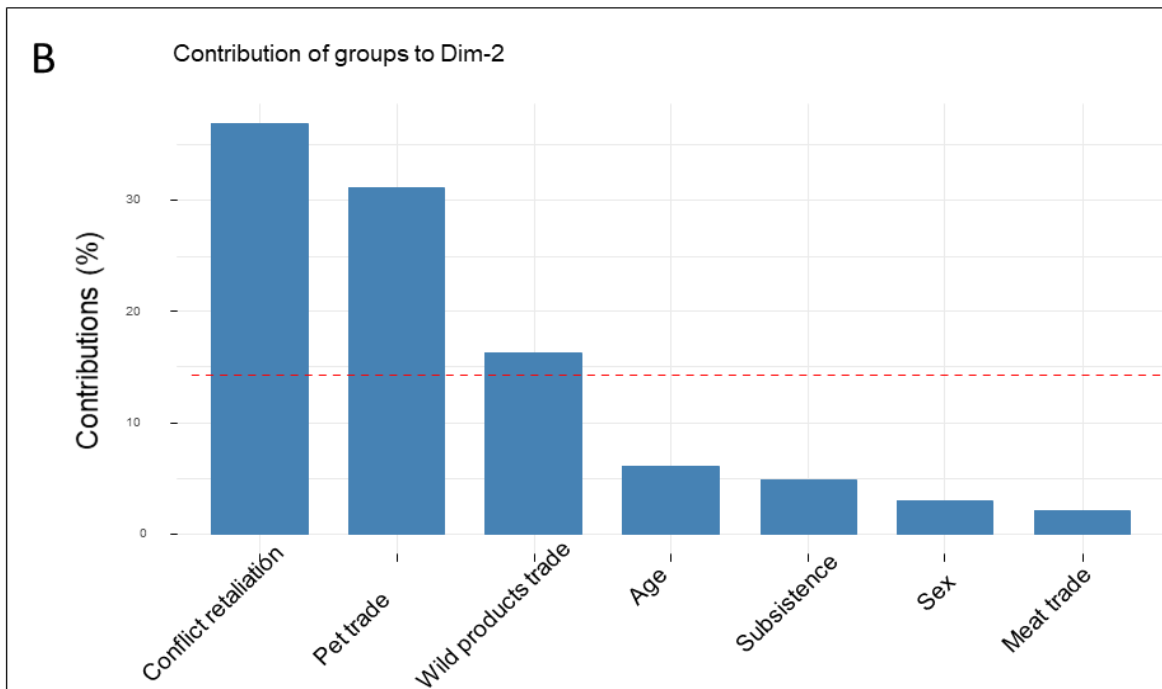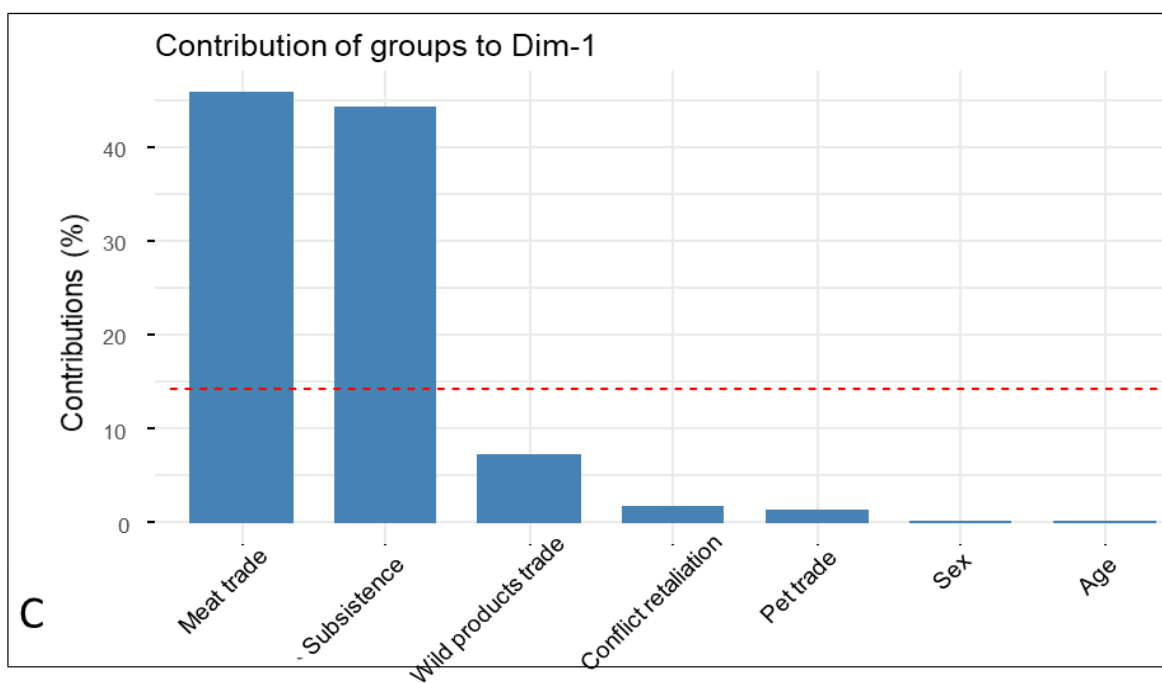

Supplement: S2 Fig — It was obtain from (a) a multiple factor analysis (MFA). Contributions of each hunting motivation and interviewee sex and age for (b) the first (Dim1) and (c) the second dimension (Dim2). The red dotted line indicates the percentage that would be obtained if all factors contributed equally to the overall variance. (PDF) [file pone.0261198.s002.pdf]

# Individual factor map

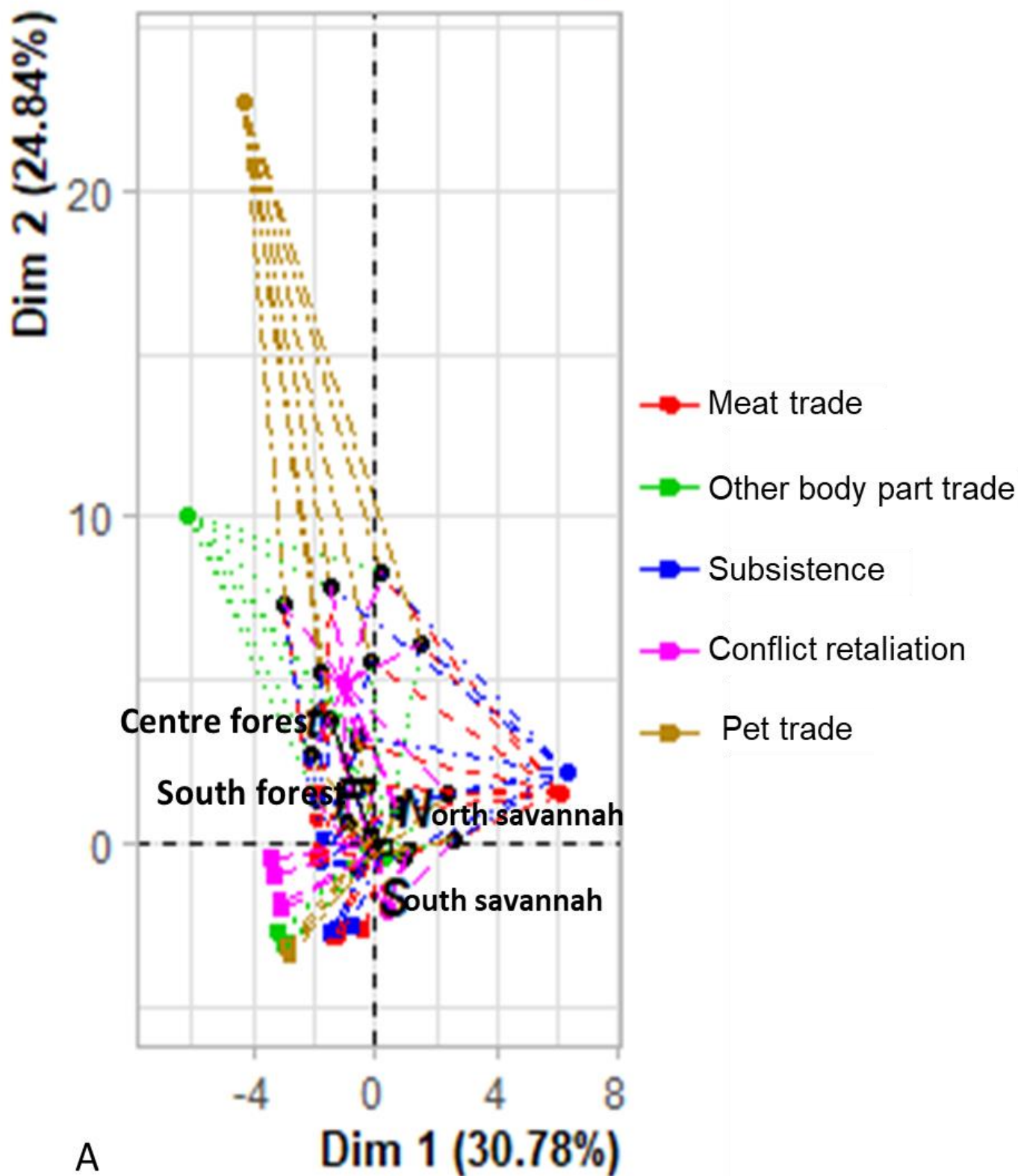

A

Supplement: S3 Fig — (PDF) [file pone.0261198.s003.pdf]
