# Supplementary material for: Intrinsic and extrinsic motivations governing prey choice by hunters in a post-war African forest-savannah macromosaic
Source: PLoS One. 2021 Dec 20;16(12):e0261198. doi: 10.1371/journal.pone.0261198 (PMC8687528; doi:10.1371/journal.pone.0261198)
Supplement: S1 Table — (PDF) [file pone.0261198.s004.pdf]

Table 1- Nonvolant terrestrial game mammal species larger than 1 kg recorded in hunting motivation within Quiçama National Park and Quiçama Game Reserve, Angola.

English name	Scientific name	Body mass (Kg)	Annual fecundity rate	Trophic level	Monetary value per animal
Thick-tailed Galago	Otolemur crassicaudatus	1	0.6	2.4	0
Talapoin	Miopithecus talapoin	1	0.6	3	0
African Savannah Hare	Lepus capensis	2	4.3	3	4
Blotched Genet	Genetta genetta	2	18	6.1	0
Marsh Cane Rat	Thryonomys swinderianus	4	1.7	2.8	3
Blue Duiker	Philantomba monticola	5	0.6	2.6	6
Vervet Malbrouck Monkey	Chlorocebus cynosurus	5	0.5	3.2	16
Blue Monkey	Cercopithecus mitis	5	0.4	2.5	22
Honey badger	Mellivora capensis	9	2.35	5.5	0
Serval	Leptailurus serval	12	1	7	20
African Civet	Civettictis civetta	12	12	4.6	30
Cape Porcupine	Hystrix africaeaustralis	15	11	2.8	10
Common Duiker	Sylvicapra grimmia	16	1	3.2	15
Wild-dog	Lycaon pictus	22	0.6	7	0
Side-striped Jackal	Canis mesomelas	22	0.6	5.2	0
Bushbuck Kewel	Tragelaphus scriptus	43	0.7	3	70
African Leopard	Panthera pardus	52	0.8	7	400
Aardvark	Orycteropus afer	56	0.6	5	70
Common Reedbuck	Redunca arundinum	58	0.6	3	141
Hyena	Crocuta crocuta	63	1	7	0
Bushpig	Potamochoerus larvatus	69	15	3.3	114
Lion	Panthera leo	159	0.7	6.8	400
Roan Antelope	Hippotragus equinus	264	0.6	3	900
Eland	Tragelaphus oryx	563	0.6	1.3	900
Hippopotamus	Syncerus caffer	593	0.2	3	900
Red Buffalo	Hippopotamus amphibius	1536	0.3	3	1500
Elephant	Loxodonta africana	3825	0.2	3	3117
