# Supplementary material for: Intrinsic and extrinsic motivations governing prey choice by hunters in a post-war African forest-savannah macromosaic
Source: PLoS One. 2021 Dec 20;16(12):e0261198. doi: 10.1371/journal.pone.0261198 (PMC8687528; doi:10.1371/journal.pone.0261198)
Supplement: S1 File — (PDF) [file pone.0261198.s005.pdf]

Supplementary material

Intrinsic and extrinsic motivations governing prey choice by hunters in a post-war African forest-savannah macromosaic

Questionnaire (English version)

General questions

Interviewee name:__________________; Age:______; Gender:_____;

Occupation(s):____________________________;

Income obtained from the mentioned occupation: ___________________;

Community name:_____________; landscape type:_____________;

geographic coordinate:

For each game species reported as killed by the interviewed hunter, he/ she was asked to provide information on the (i) the date of the most recent mammal specimen killing; (ii) sex and age class of the most commonly hunted individuals; (iii) motivation behind the pursuit each wild mammals ; and (iv) the revenue acquisition from the sale of the animal when traded including the trade value (per kg) as: a) live whole animal, b) whole dead animal (e.g. fresh meat), c) processed as dried meat, d) processed skin, and e) ivory. We were therefore able to quantify the total aggregate value of each prey item.

Game species questions

From the visual stimulus checklist, list the species you hunt:

For each reported species, cite the date of the most recent specimen capturing;

For each reported species, cite the sex and age class of the most commonly specimen capturing;

For each reported species, cite the motivation(s) behind its pursuit;

For each reported species, cite the revenue acquired from its sale when traded including the trade value (per kg) of: a) live whole animal, b) whole dead animal (e.g. fresh meat), c) processed as dried meat, d) processed skin, and e) ivory.

Questionário (versão em português)

Perguntais gerais

Nome do entrevistado: ___________________; Idade:______; Sexo:_____;

Ocupação (ões): ______________________________;

Renda obtido na referida ocupação: ____________________;

Nome da comunidade: ______________; tipo de paisagem: ______________;

coordenada geográfica:_________________

Perguntas associadas as espécies cinegéticas

A partir das fotografias para estímulo visual, liste quais espécies você caça:

Para cada espécie mencionada, cite a data do espécime mais recente capturado;

Para cada espécie mencionada, cite o sexo e a idade dos espécimes mais comumente caçados;

Para cada espécie mencionada, cite a(s) motivação(ões) para seu abate;

Para cada espécie mencionada, cite o lucro obtido com a sua venda incluindo o valor (por kg) de: a) animal inteiro vivo, b) animal inteiro morto (por exemplo, carne fresca), c) processado como carne seca, d) pele processada e e) marfim
