# Supplementary material for: Intrinsic and extrinsic motivations governing prey choice by hunters in a post-war African forest-savannah macromosaic
Source: PLoS One. 2021 Dec 20;16(12):e0261198. doi: 10.1371/journal.pone.0261198 (PMC8687528; doi:10.1371/journal.pone.0261198)
Supplement: Attachment — Submitted filename: Response_letter.pdf [file pone.0261198.s006.pdf]

Angola, July 2021.

From: Franciany B-P.
Franbraga83@yahoo.com.br

To: Emmanuel Serrano
Academic Editor of Plos One

Dear Editor,

We are submitting a new version of our manuscript (ID: **PONE-D-21-09230**) entitled "**Intrinsic and extrinsic motivations governing prey choice by hunters in a post-war African forest-savannah macromosaic". **We would like to thank the reviewer for the comprehensive comments and suggestions on the paper. We appreciate the feedback and believe that responding to these comments have improved the quality of our manuscript.
Please find below a point-by-point response to the comments provided by the Reviewer. We are providing two versions of the Manuscript, one with and other without track changes, so that you can see exactly what has been altered. Note that the lines indicated below is referring to the **clean version** of the Manuscript.

Yours sincerely

Franciany G. Braga-Pereira (on behalf of all co-authors)

Journal: Plos One

Ref:PONE-D-21-09230

Reviewer #1: The main merits of this manuscript are (1) using local knowledge to provide an interesting, useful and easy methodology to (2) estimate the motivations of hunters in a post-war area. In many places worldwide, the only data available for management and conservation proposes is the ecological local knowledge (besides hunting bags when available), therefore any method focused on using this knowledge to produce population estimates (management) and the motivations for killing certain species (conservation) will be proven very helpful, especially when referring to natural areas or countries with strong budget limitations or low-income economies.

As a matter of fact, it is impossible to change a person's behavior towards wildlife (e.g., feeding or poaching) without knowing the motivations behind that behaviour. This study addresses a stated need of new strategies which, based on the motivations towards hunting certain species, help to better plan for the management of ecosystems that are recovering from strong disturbances, such as wars, and where poaching is present with strong impact on the recovery of wildlife populations.

This is a sound-based manuscript; however, I have some remarks on the text.

Authors: We are very grateful for these positive comments and effort to improve our manuscript. We followed all of your suggestions and we believe that our manuscript has been further strengthened after these recommendations.

Reviewer #1

L79-82: I may not have understood this part of the manuscript correctly, so in that case the authors should state it clearer, but when asking about the motivation behind the pursuit each wild mammals during the semi-structured individual interviews, did the authors used a closed questionary based on Muth and Bowe 1998, and the rest of the intrinsic, extrinsic and individual based

motivations mentioned in the manuscript, or did you allowed the hunters to relate their motivations without any "constrain"?

I ask this question since you the authors had the opportunity to collect first-hand the motivations of hunters in a post-war area, I think that the appearance of unpredicted motivations could have had a great importance, both for the results and for the impact of the present manuscript.

Authors: We agree with your comments regarding the opportunity to first-hand collect the motivations of hunters and we confirmed we allowed the hunters to relate their motivations without any "constrain". We had improve the main manuscript for a better explanation on that (lines 186-199) and we provided the whole questionnaire used in this study in the supplementary material.

Reviewer #1 Moreover, I think the discussion of the manuscript could be improved if the results of this study were compared with other studies, stating similarities and differences with the motivations of poachers in other post-war areas (if any, and ideally in other continents), or in non-post-war areas, such as the study cited Muth and Bowe 1998, in the USA, where the conditions for poachers were totally different.

Authors: We agree with you and had improved the discussion on that (lines 452-460; 510-514; 521-545)

Reviewer 1# L211 Data analysis. I have several concerns about the data analysis: The authors interviewed 115 hunters with different ages (19-80 years old), from eight settlements (one located in the Game reserve), with different population densities and located in different ecosystems (forest-savannah). If I was a manager of this or a similar natural area wanting to implement conservation strategies based on working with the communities, I would find attractive your study but after reading the manuscript, several questions would come to my mind. Are the motivations for hunting the same along all ages and settlements regardless of their differences? Besides stating the differences in the

activity among male and female and adults, children and the elderly, the manuscript would be improved if the authors evaluated possible differences in the motivations due to the hunters' characteristics.

As the authors did not evaluated differences due to hunters' characteristics, why did the authors used a GLM instead of a GLMM, where the hunter and the settlement were included as nested random effects? The results would be more robust.

But as I stated before, I think that the authors are not taking full advantage of what they could get from the data. In my opinion and based on my experience, to study the motivations of human behaviors towards wildlife based on surveys, the multivariate statistical tests can give you more information, especially those based on the canonical ordination, such as multiple factor analysis (http://www.sthda.com/english/articles/31-principal-component-methods-in-r-practical-guide/116-mfa-multiple-factor-analysis-in-r-essentials/).

Authors: We appreciated this comment. We had performed a MFA for better understand the similarities of species selected to attend different hunting motivations regarding species body mass and energetic level; sampled site; and interviewee age and sex.

In addition, to examine the effects of species body mass and trophic level; and interviewee age, gender and natal community on the number of hunters reporting each hunting motivation for killing any given species we performed generalized linear mixed models (GLMM) and generalized linear models (GLM). We performed a GLMM considering the interviewee as a random variable; and the body mass and energetic level as fixed predict variable.

We assigned the 8 sampled communities into four study sites according to type of landscape structure (forest of savannah) and geographic position. We therefore sampled two communities in savannah areas of northern Quiçama; two in savannah areas of southern Quiçama; two in central forest areas; and two communities in southern forest areas. By this way we could better explain our results for readers not familiar to the area and in the same time to ensure the interviewee confidentiality, as some communities contained few hunters.

See lines in the Methods section: 217-228; 232-249; 260-272.

See lines in the Results section: 313-328; Table 1 (355); 396-409.

See lines in the Discussion section: 489-509; 562-567.

See Supplementary Material: Figures 2 and 3.

Reviewer 1#L44-46: I recommend to review the citations in text and references. Just as an example I found two mistakes between L82 and L86 (e.g. In L82 you cited (Muth and Bowe 1998) but I cannot find the citation in references, L86-87 you cited as (Knapp and Peace 2017) but the article has three authors.

Authors: Thank you for your comment. We have reviewed the citations and references.

Reviewer1# L199-201: Again, I may not have understood this part correctly, if so, the authors should state it clearer. In the example: "if the grazer/frugivore Tragelaphus oryx consumes 70% grass and 30% fruit, its mean trophic energy level would be 2.7 (= (0.7 × 3) + (0.3 × 2)", but according to the levels in L195-197 ((1) grazer/folivore < (2) frugivore < (3) granivore…) the level should be 1.3 (= (0.7 × 1) + (0.3 × 2).

Authors: Thank you for your comment. We corrected the text (line 203-208).

Reviewer 1#L343-367: The authors should state the usefulness of ecological local knowledge as a tool to estimate wildlife abundance.

Authors:

Thank you for your comment. We agree and had edited the text (lines:621-624).
