# Supplementary material for: Intrinsic and extrinsic motivations governing prey choice by hunters in a post-war African forest-savannah macromosaic
Source: PLoS One. 2021 Dec 20;16(12):e0261198. doi: 10.1371/journal.pone.0261198 (PMC8687528; doi:10.1371/journal.pone.0261198)
Supplement: Attachment — Submitted filename: PONE-D-21-09230_R1_Revised.pdf [file pone.0261198.s007.pdf]

PLOS ONE

Intrinsic and extrinsic motivations governing prey choice by hunters in a post-war African forest-savannah macromosaic

--Manuscript Draft--

Manuscript Number:	PONE-D-21-09230R1
Article Type:	Research Article
Full Title:	Intrinsic and extrinsic motivations governing prey choice by hunters in a post-war African forest-savannah macromosaic
Short Title:	motivations governing prey choice by hunters in a post-war African forest-savannah macromosaic
Corresponding Author:	Franciany Braga-Pereira<br>Universidade Federal da Paraiba<br>Joao Pessoa, PB BRAZIL
Keywords:	Angola; ethnobiology; Ethnozoology; local knowledge; Mammals; illegal hunting; Traditional knowledge; wildlife trade
Abstract:	Overhunting typically increases during and after armed conflicts, and may lead to regional-scale defaunation. The mitigation of hunting impacts is complex because, among other reasons, several intrinsic and extrinsic motivations underpin the elevated deployment of hunting practices. Here we present the first study focusing on these motivations in a post-war zone. Following persistently heavy hunting pressure during the 27-year Angolan civil war, prey choice associated with different motivations varied in terms of species trophic level and body size. While most residents hunted large-bodied species to maximize revenues from wildlife trade, many low-trophic level, smaller species were harvested to meet local subsistence demands because they were more palatable and could be captured using artisanal traps near hunters' households. Small-bodied species across all trophic levels were also frequently killed in retaliation for crop-raiding or livestock depredation. Considering all game species sampled in this study, 96% were captured to attend two or more motivations. In addition, hunting associated with different motivations was partitioned in terms of age and gender, with prey acquisition for the wildlife trade primarily carried out by adult men, while hunting to meet local subsistence needs and inhibit human-wildlife conflicts were carried out by adult men and women, children and even the elderly. In natural savannah areas lacking fish as a source of protein, a higher number of species was selected to supply both the meat trade and subsistence, while more species in forest areas were targeted for trade in animal body parts and conflict retaliation. Finally, local commerce in bushmeat and other body parts accrued higher domestic revenues compared to any alternative sources of direct and indirect income. However, these financial benefits were at best modest, largely unsustainable in terms of prey population collapses, and generated high long-term costs for the local to regional scale economy and native biodiversity.
Order of Authors:	Franciany Braga-Pereira
	Carlos A. Peres
	Rômulo Romeu da Nóbrega Alves
	Carmén Van-Dúnem Santos
Opposed Reviewers:	
Response to Reviewers:	To: Emmanuel Serrano<br>Academic Editor of Plos One<br>Dear Editor,<br>We are submitting a new version of our manuscript (ID: PONE-D-21-09230)<br>entitled "Intrinsic and extrinsic motivations governing prey choice by<br>hunters in a post-war African forest-savannah macromosaic". We would<br>like to thank the editor and reviewer for the comprehensive comments and suggestions on<br>the paper. We appreciate the feedback and believe that responding to these

Powered by Editorial Manager® and ProduXion Manager® from Aries Systems Corporation

comments have improved the quality of our manuscript.
Please find below a point-by-point response to the comments provided by the Editor and
Reviewer. We are providing two versions of the Manuscript, one with and other without track changes, so that you can see exactly what has been altered. Note that the lines indicated below is referring to the clean version of the Manuscript.
Yours sincerely
Franciany G. Braga-Pereira (on behalf of all co-authors)Journal: Plos One
Ref:PONE-D-21-09230

We included in the analysis results of the linear models the F-value, df and Adjusted R-squared (lines 252-257; 370-373). For generalized linear models and generalized linear mixed models we used residual checks to verify whether our models were, in principle, suitable or otherwise. Finally, we used the Akaike information criterion to select models of interest if ∆AIC values >6 (∆AIC obtain from the difference between a null and complete model AIC values). ∆AIC>6 was chosen following Harrison et al., (2018) and Richards (2008) (lines 240-243; 265-268).

We included the questionnaire in both in the original language (Portuguese) and in English, as Supporting Information.

The map we published in Elsevier (https://www.sciencedirect.com/science/article/abs/pii/S0006320720308028) is under copyright, which means the map used in the previous version of this article was also under copyright. We contacted Elsevier to request a license to use the images, however we didn't get a return, so we made a new map.
Because of that, below, we have included two map options. Option 1 is similar the original map, but not the same. If it is possible to use this option 1, it would interest as it demarcates the savannah and forest landscapes. If, because of the similarity between this new map and the original one, you choose a more different image; we have created a second map option (Option 2)

People involved in the research answered questions about hunting, which is an illegal activity in the region. For this reason, we need to maintain the anonymityof informants, because data contain potentially sensitive information imposed by UFPB ethics committee (email: comitedeetica@ccs.ufpb.br).

Results presented herein are entirely original and this paper is not being considered for publication in other journal. All sources of funding are acknowledged in the manuscript, and all appropriate ethics and other approvals were obtained for this research. This research project was registered in the Plataforma Brasil, which is a Brazilian database of research records involving human communities and prior research approval was obtained from the Research Ethics Committee of the Health Sciences Center at the Federal University of Paraiba - (CEP -CCS- UFPB-CAAE 59846816.3.0000.5188). This study was also authorized by the Environmental Ministry of Angola (Ministério do Ambiente de Angola) based on license number 148INBAC. MINAMB/2016 and by all local community leaders of human settlements in which this research was conducted. We also received a municipal authorization from the mayor of Quiçama (city of Angola) via license number 017 / GAB.ADM.M.Q / 2017.
We confirm the first author of the manuscript submitted to Plos One (Franciany Braga-Pereira) is the author of the map used in the manuscript.

Reviewer #1: The main merits of this manuscript are (1) using local knowledge to provide an interesting, useful and easy methodology to (2) estimate the motivations of hunters in a post-war area. In many places worldwide, the only data available for management and conservation proposes is the ecological local knowledge (besides hunting bags when available), therefore any method focused on using this knowledge to produce population estimates (management) and the motivations for killing certain species (conservation) will be proven very helpful, especially when referring to natural areas or countries with strong budget limitations or low-income economies.
As a matter of fact, it is impossible to change a person's behavior towards wildlife

Powered by Editorial Manager® and ProduXion Manager® from Aries Systems Corporation

(e.g., feeding or poaching) without knowing the motivations behind that behaviour. This study addresses a stated need of new strategies which, based on the motivations towards hunting certain species, help to better plan for the management of ecosystems that are recovering from strong disturbances, such as wars, and where poaching is present with strong impact on the recovery of wildlife populations.
This is a sound-based manuscript; however, I have some remarks on the text.
Authors: We are very grateful for these positive comments and effort to improve our manuscript. We followed all of your suggestions and we believe that our manuscript has been further strengthened after these recommendations.
Reviewer #1
L79-82: I may not have understood this part of the manuscript correctly, so in that case the authors should state it clearer, but when asking about the motivation behind the pursuit each wild mammals during the semi-structured individual interviews, did the authors used a closed questionary based on Muth and Bowe 1998, and the rest of the intrinsic, extrinsic and individual basedmotivations mentioned in the manuscript, or did you allowed the hunters to relate their motivations without any "constrain"?
I ask this question since you the authors had the opportunity to collect first-hand the motivations of hunters in a post-war area, I think that the appearance of unpredicted motivations could have had a great importance, both for the results and for the impact of the present manuscript.
Authors: We agree with your comments regarding the opportunity to first-hand collect the motivations of hunters and we confirmed we allowed the hunters to relate their motivations without any "constrain". We had improve the main manuscript for a better explanation on that (lines 186-199) and we provided the whole questionnaire used in this study in the supplementary material.
Reviewer #1 Moreover, I think the discussion of the manuscript could be improved if the results of this study were compared with other studies, stating similarities and differences with the motivations of poachers in other post-war areas (if any, and ideally in other continents), or in non-post-war areas, such as the study cited Muth and Bowe 1998, in the USA, where the conditions for poachers were totally different.
Authors: We agree with you and had improved the discussion on that (lines 452-460; 510-514; 521-545)
Reviewer 1# L211 Data analysis. I have several concerns about the data analysis: The authors interviewed 115 hunters with different ages (19-80 years old), from eight settlements (one located in the Game reserve), with different population densities and located in different ecosystems (forest-savannah). If I was a manager of this or a similar natural area wanting to implement conservation strategies based on working with the communities, I would find attractive your study but after reading the manuscript, several questions would come to my mind. Are the motivations for hunting the same along all ages and settlements regardless of their differences? Besides stating the differences in theactivity among male and female and adults, children and the elderly, the manuscript would be improved if the authors evaluated possible differences in the motivations due to the hunters' characteristics.
As the authors did not evaluated differences due to hunters' characteristics, why did the authors used a GLM instead of a GLMM, where the hunter and the settlement were included as nested random effects? The results would be more robust.
But as I stated before, I think that the authors are not taking full advantage of what they could get from the data. In my opinion and based on my experience, to study the motivations of human behaviors towards wildlife based on surveys, the multivariate statistical tests can give you more information, especially those based on the canonical ordination, such as multiple factor analysis (http://www.sthda.com/english/articles/31-principal-component-methods-in-rpractical-guide/116-mfa-multiple-factor-analysis-in-r-essentials/).
Authors: We appreciated this comment. We had performed a MFA for better understand the similarities of species selected to attend different hunting motivations regarding species body mass and energetic level; sampled site; and interviewee age and sex.
In addition, to examine the effects of species body mass and trophic level; and interviewee age, gender and natal community on the number of hunters reporting

Powered by Editorial Manager® and ProduXion Manager® from Aries Systems Corporation

	each hunting motivation for killing any given species we performed generalized linear mixed models (GLMM) and generalized linear models (GLM). We performed a GLMM considering the interviewee as a random variable; and the body mass and energetic level as fixed predict variable.<br>We assigned the 8 sampled communities into four study sites according to type of landscape structure (forest of savannah) and geographic position. We therefore sampled two communities in savannah areas of northern Quiçama; two in savannah areas of southern Quiçama; two in central forest areas; and two communities in southern forest areas. By this way we could better explain our results for readers not familiar to the area and in the same time to ensure the interviewee confidentiality, as some communities contained few hunters.See lines in the Methods section: 217-228; 232-249; 260-272.<br>See lines in the Results section: 313-328; Table 1 (355); 396-409.<br>See lines in the Discussion section: 489-509; 562-567.<br>See Supplementary Material: Figures 2 and 3.<br>Reviewer 1#L44-46: I recommend to review the citations in text and references. Just as an example I found two mistakes between L82 and L86 (e.g. In L82 you cited (Muth and Bowe 1998) but I cannot find the citation in references, L86-87 you cited as (Knapp and Peace 2017) but the article has three authors.<br>Authors: Thank you for your comment. We have reviewed the citations and references.<br>Reviewer1# L199-201: Again, I may not have understood this part correctly, if so, the authors should state it clearer. In the example: "if the grazer/frugivore Tragelaphus oryx consumes 70% grass and 30% fruit, its mean trophic energy level would be 2.7 (= (0.7 × 3) + (0.3 × 2)", but according to the levels in L195-197 ((1) grazer/folivore < (2) frugivore < (3) granivore…) the level should be 1.3 (= (0.7 × 1) + (0.3 × 2).<br>Authors: Thank you for your comment. We corrected the text (line 203-208).<br>Reviewer 1#L343-367: The authors should state the usefulness of ecological local knowledge as a tool to estimate wildlife abundance.<br>Authors:<br>Thank you for your comment. We agree and had edited the text (lines:621-624)
**Additional Information:**	
**Question**	**Response**
**Financial Disclosure**<br><br>Enter a financial disclosure statement that describes the sources of funding for the work included in this submission. Review the submission guidelines for detailed requirements. View published research articles from *PLOS ONE* for specific examples.<br><br>This statement is required for submission and **will appear in the published article** if the submission is accepted. Please make sure it is accurate.	FB-P was supported by the Faculdade de Ciências-Universidade de Agostinho Neto (Angola), by The Rufford Foundation (ID 20153-1) and by an MSc scholarship granted by the Brazilian Ministry of Education (CAPES, 20151025938). CVDS was supported by Faculdade de Ciências-Universidade de Agostinho Neto (Angola). RRNA was supported by a productivity research grant of National Council for Scientific and Technological Development (CNPq).<br><br>Universidade de Agostinho Neto: https://uan.ao/<br>The Rufford Foundation: https://www.rufford.org/projects/franciany-gabriella-braga-pereira/hunting-and-use-of-wild-mammals-by-human-communities-in-a-conservation-area-in-angola-the-influence-of-environment-on-hunterss-niche-breadth/<br>CAPES: https://www.gov.br/capes/pt-br<br>CNPq:https://www.gov.br/cnpq/pt-br<br><br>Did the sponsors or funders play any role in the study design, data collection and analysis, decision to publish, or preparation of the manuscript?<br>•NO

**Unfunded studies**<br>Enter: *The author(s) received no specific funding for this work.*<br><br>**Funded studies**<br>Enter a statement with the following details:<br>• Initials of the authors who received each award<br>• Grant numbers awarded to each author<br>• The full name of each funder<br>• URL of each funder website<br>• Did the sponsors or funders play any role in the study design, data collection and analysis, decision to publish, or preparation of the manuscript?<br>• **NO** - Include this sentence at the end of your statement: *The funders had no role in study design, data collection and analysis, decision to publish, or preparation of the manuscript.*<br>• **YES** - Specify the role(s) played.<br><br>* typeset	
**Competing Interests**<br><br>Use the instructions below to enter a competing interest statement for this submission. On behalf of all authors, disclose any competing interests that could be perceived to bias this work—acknowledging all financial support and any other relevant financial or non-financial competing interests.<br><br>This statement is required for submission and **will appear in the published article** if the submission is accepted. Please make sure it is accurate and that any funding sources listed in your Funding Information later in the submission form are also declared in your Financial Disclosure statement.<br><br>View published research articles from *PLOS ONE* for specific examples.	The authors have declared that no competing interests exist.

**NO authors have competing interests**<br><br>Enter: *The authors have declared that no competing interests exist.*<br><br>**Authors with competing interests**<br><br>Enter competing interest details beginning with this statement:<br><br>*I have read the journal's policy and the authors of this manuscript have the following competing interests: [insert competing interests here]*<br><br>* typeset	
**Ethics Statement**<br><br>Enter an ethics statement for this submission. This statement is required if the study involved:<br><br>• Human participants<br>• Human specimens or tissue<br>• Vertebrate animals or cephalopods<br>• Vertebrate embryos or tissues<br>• Field research<br><br>Write "N/A" if the submission does not require an ethics statement.<br><br>General guidance is provided below. Consult the submission guidelines for detailed instructions. **Make sure that all information entered here is included in the Methods section of the manuscript.**	This research was approved by the i) National Health Council (Resolution 466/12), through the Research Ethics Committee of the Universidade Federal da Paraíba (license<br>number 59846816.3.0000.5188); ii) Environmental Ministry of Angola (148INBAC.MINAMB/2016); iii) municipal administration of Quiçama (license number 017/GAB.ADM.MQ/2017); iv) leaderships of all sampled villages. An informed consent form was given to the interviewee, stating the purpose of the interview and ensuring the interviewee's anonymity, so each interview could not begin until after this form had been signed.

Powered by Editorial Manager® and ProduXion Manager® from Aries Systems Corporation

**Format for specific study types**<br><br>**Human Subject Research (involving human participants and/or tissue)**<br>• Give the name of the institutional review board or ethics committee that approved the study<br>• Include the approval number and/or a statement indicating approval of this research<br>• Indicate the form of consent obtained (written/oral) or the reason that consent was not obtained (e.g. the data were analyzed anonymously)<br><br>**Animal Research (involving vertebrate animals, embryos or tissues)**<br>• Provide the name of the Institutional Animal Care and Use Committee (IACUC) or other relevant ethics board that reviewed the study protocol, and indicate whether they approved this research or granted a formal waiver of ethical approval<br>• Include an approval number if one was obtained<br>• If the study involved *non-human primates*, add *additional details* about animal welfare and steps taken to ameliorate suffering<br>• If anesthesia, euthanasia, or any kind of animal sacrifice is part of the study, include briefly which substances and/or methods were applied<br><br>**Field Research**<br><br>Include the following details if this study involves the collection of plant, animal, or other materials from a natural setting:<br>• Field permit number<br>• Name of the institution or relevant body that granted permission	
**Data Availability**<br><br>Authors are required to make all data underlying the findings described fully available, without restriction, and from the time of publication. PLOS allows rare exceptions to address legal and ethical concerns. See the PLOS Data Policy and FAQ for detailed information.	No - some restrictions will apply

Powered by Editorial Manager® and ProduXion Manager® from Aries Systems Corporation

A Data Availability Statement describing where the data can be found is required at submission. Your answers to this question constitute the Data Availability Statement and **will be published in the article**, if accepted.<br><br>**Important:** Stating 'data available on request from the author' is not sufficient. If your data are only available upon request, select 'No' for the first question and explain your exceptional situation in the text box.<br><br>Do the authors confirm that all data underlying the findings described in their manuscript are fully available without restriction?	
**Describe where the data may be found in full sentences. If you are copying our sample text, replace any instances of XXX with the appropriate details.**<br><br>• If the data are **held or will be held in a public repository**, include URLs, accession numbers or DOIs. If this information will only be available after acceptance, indicate this by ticking the box below. For example: *All XXX files are available from the XXX database (accession number(s) XXX, XXX.*).<br>• If the data are all contained **within the manuscript and/or Supporting Information files**, enter the following: *All relevant data are within the manuscript and its Supporting Information files.*<br>• If neither of these applies but you are able to provide **details of access elsewhere**, with or without limitations, please do so. For example:<br><br>*Data cannot be shared publicly because of [XXX]. Data are available from the XXX Institutional Data Access / Ethics Committee (contact via XXX) for researchers who meet the criteria for access to confidential data.*<br><br>*The data underlying the results presented in the study are available from (include the name of the third party*	Data cannot be shared publicly because it contains potentially identifying details of the poachers. Data are available from the Research Ethics Committee (CEP) / Health Sciences Center (CCS) of the Federal University of Paraiba (UFPB) (contact via comitedeetica@ccs.ufpb.br) for researchers who meet the criteria for access to confidential data.

and contact information or URL).<br>• This text is appropriate if the data are owned by a third party and authors do not have permission to share the data.<br><br>* typeset	
Additional data availability information:	

Powered by Editorial Manager® and ProduXion Manager® from Aries Systems Corporation

Angola, July, 2021.

From: Franciany B-P.
Franbraga83@yahoo.com.br

To: Emmanuel Serrano

Academic Editor of Plos One

Dear editor,

We are submitting a new version of our manuscript (ID: PONE-D-21-09230R1) entitled "**Intrinsic and extrinsic motivations governing prey choice by hunters in a post-war African forest-savannah macromosaic**" to Plos One. We would like to thank you for the comments and suggestions on the paper.

Please, find below our comments for your previous questions.

We included in the analysis results of the linear models the F-value, df and Adjusted R-squared (lines 252-257; 370-373). For generalized linear models and generalized linear mixed models we used residual checks to verify whether our models were, in principle, suitable or otherwise. Finally, we used the Akaike information criterion to select models of interest if ΔAIC values >6 (ΔAIC obtain from the difference between a null and complete model AIC values). ΔAIC>6 was chosen following Harrison et al., (2018) and Richards (2008) (lines 240-243; 265-268).

We included the questionnaire in both in the original language (Portuguese) and in English, as Supporting Information.

The map we published in Elsevier (https://www.sciencedirect.com/science/article/abs/pii/S0006320720308028) is under copyright, which means the map used in the previous version of this article was also under copyright. We contacted Elsevier to request a license to use the images, however we didn't get a return, so we made a new map.

Because of that, below, we have included two map options. Option 1 is similar the original map, but not the same. If it is possible to use this option 1, it would interest as it demarcates the savannah and forest landscapes. If, because of the similarity between this new map and the original one, you choose a more different image; we have created a second map option (Option 2).

Option 1

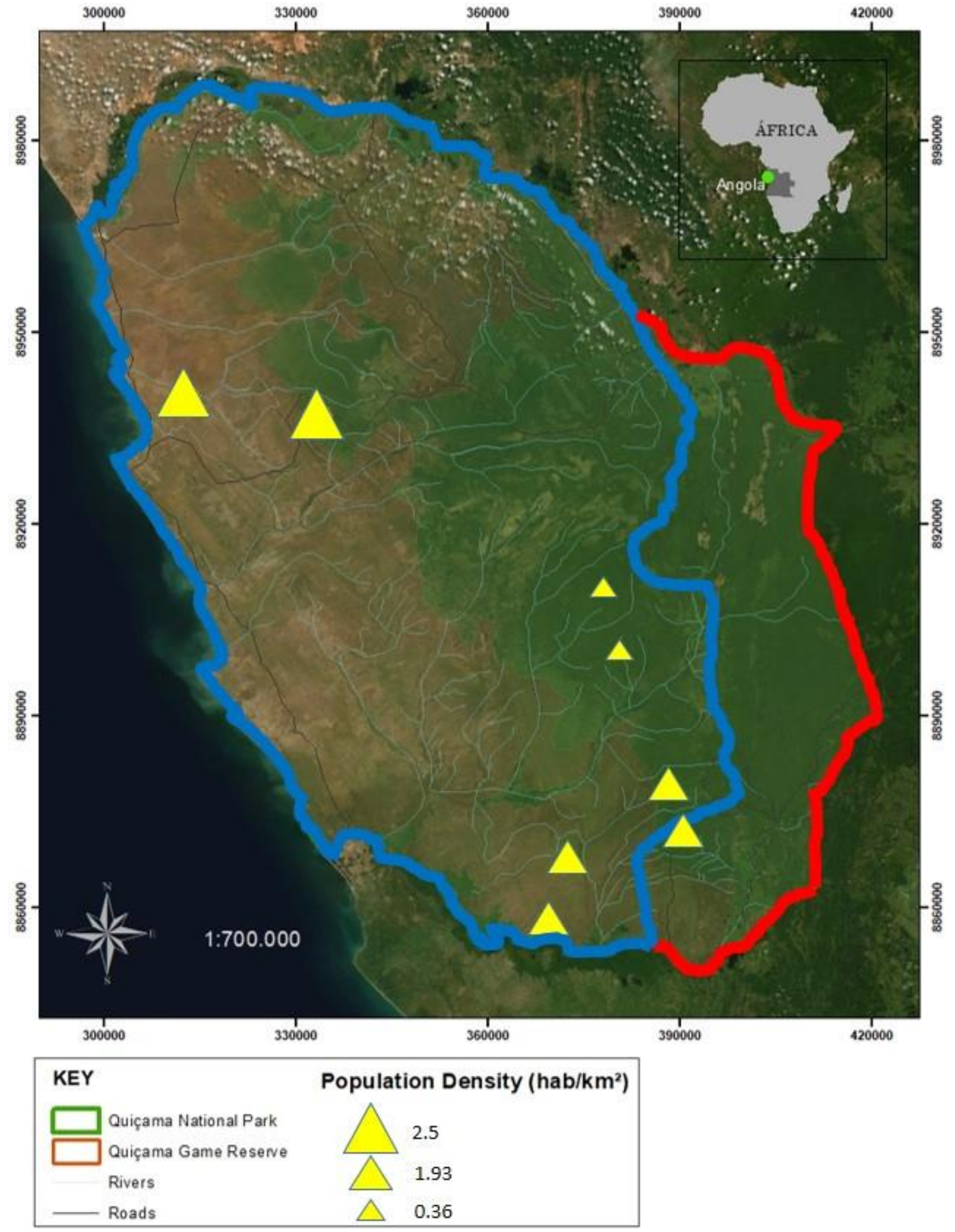
300000
330000
360000
390000
420000
8980000
8950000
8920000
8890000
8860000
ÁFRICA
Angola
1:700.000
KEY
Population Density (hab/km²)
Quiçama National Park
Quiçama Game Reserve
Rivers
Roads
2.5
1.93
0.36

Option 2

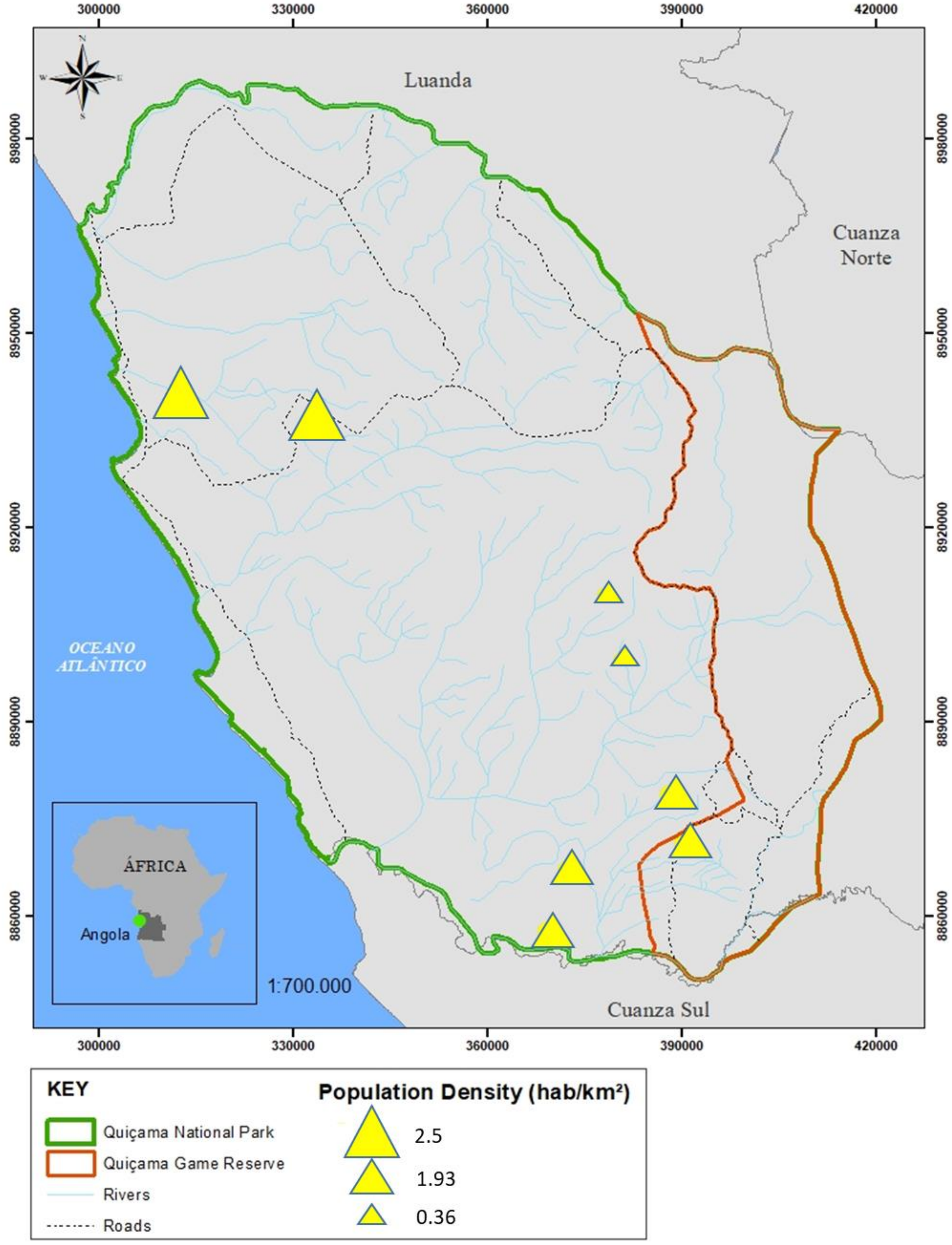

People involved in the research answered questions about hunting, which is an illegal activity in the region. For this reason, we need to maintain the anonymity

of informants, because data contain potentially sensitive information imposed by UFPB ethics committee (email: eticaccsufpb@hotmail.com).

Results presented herein are entirely original and this paper is not being considered for publication in other journal. All sources of funding are acknowledged in the manuscript, and all appropriate ethics and other approvals were obtained for this research. This research project was registered in the Plataforma Brasil, which is a Brazilian database of research records involving human communities and prior research approval was obtained from the Research Ethics Committee of the Health Sciences Center at the Federal University of Paraiba - (CEP -CCS- UFPB-CAAE 59846816.3.0000.5188). This study was also authorized by the Environmental Ministry of Angola (Ministério do Ambiente de Angola) based on license number 148INBAC. MINAMB/2016 and by all local community leaders of human settlements in which this research was conducted. We also received a municipal authorization from the mayor of Quiçama (city of Angola) via license number 017 / GAB.ADM.M.Q / 2017.

We confirm the first author of the manuscript submitted to Plos One (Franciany Braga-Pereira) is the author of the map used in the manuscript.

Yours truly,

Franciany G. Braga-Pereira (on behalf of all co-authors)

Manuscript Click here to access/download;Manuscript;Manuscript.docx

Intrinsic and extrinsic motivations governing prey choice by hunters in a post-war African forest-savannah macromosaic

Franciany Braga-Pereira [1,2,3*]; Carlos A. Peres [4,5]; Rômulo Romeu Nóbrega Alves [1,6]; Carmén Van-Dúnem Santos [3]

1- Department of Ecology and Systematics, Universidade Federal da Paraíba, João Pessoa, PB, Brazil

2- Rede de Pesquisa para Estudos sobre Diversidade, Conservação e Uso da Fauna na Amazônia (RedeFauna), Manaus, Amazonas – Brasil

3- Agostinho Neto University, FCUAN, Avenida 4 de Fevereiro, 71, Luanda, Angola

4-Instituto Juruá, Rua das Papoulas, 97, Aleixo, Manaus, AM, 69083-300, Brazil.

5- School of Environmental Sciences, University of East Anglia, Norwich, UK

6- Department of Biology, Universidade Estadual da Paraíba, Campina Grande, 58429-500, Brazil

*Corresponding author: franbraga83@yahoo.com.br

SUMMARY

Overhunting typically increases during and after armed conflicts, and may lead to regional-scale defaunation. The mitigation of hunting impacts is complex because, among other reasons, several intrinsic and extrinsic motivations underpin the elevated deployment of hunting practices. Here we present the first study focusing on these motivations in a post-war zone. Following persistently heavy hunting pressure during the 27-year Angolan civil war, prey choice associated with different motivations varied in terms of species trophic level and body size. While most residents hunted large-bodied species to maximize revenues from wildlife trade, many low-trophic level smaller species were harvested to meet local subsistence demands because they were more palatable and could be captured using artisanal traps near hunters' households. Small-bodied species across all trophic levels were also frequently killed in retaliation for crop-raiding or livestock depredation. Considering all game species sampled in this study, 96% were captured to attend two or more motivations. In addition, hunting associated with different motivations was partitioned in terms of age and gender, with prey acquisition for the wildlife trade primarily carried out by adult men, while hunting to meet local subsistence needs and inhibit human-wildlife conflicts were carried out by adult men and women, children and even the elderly. In natural savannah areas lacking fish as a source of protein, a higher number of species was selected to supply both the meat trade and subsistence, while more species in forest areas were targeted for trade in animal body parts and conflict retaliation. Finally, local commerce in bushmeat and other body parts accrued higher domestic revenues compared to any alternative

sources of direct and indirect income. However, these financial benefits were at best modest, largely unsustainable in terms of prey population collapses, and generated high long-term costs for the local to regional scale economy and native biodiversity.

Key- words: Angola, ethnozoology, local knowledge, mammals, illegal hunting, wildlife trade

1 INTRODUCTION

Hunting of wild terrestrial vertebrates, as one of the oldest activities pursued by both archaic and modern humans, has been undoubtedly critical in human ecology and evolution because hunting represents a form of defence against wild animals and procurement of food, clothing and therapeutic products (1–3). However, hunting practices have resulted in large-scale defaunation, and even subsistence hunting to meet local needs represents one of the primary drivers of local extinctions of wildlife populations (4–6). In many tropical regions, hunting exerts become a more severe threat to native wildlife than primary habitat loss via the conversion of natural vegetation (7,8). Moreover, the potential ecological effects of overhunting are not only on populations of game species, but also on the overall structure and dynamics of natural ecosystems (5,9), and overhunting places local livelihoods and food security at risk (10). Large-bodied mammals are often the most frequently selected prey species for the bushmeat trade due to their larger meat yields and greater returns on energy invested in hunting (11,12), in addition to being more intrinsically detectable for game hunters in open landscapes (13,14). However, these mammal species generally exhibit

lower fecundity rates, larger home ranges and lower population densities, which result in higher vulnerability to population depletion and local extirpation (15–17). Species-specific hunter selectivity is often also both size- the sex-biased, so that the impact of hunting tends to be higher on the survival of sexually mature males, particularly in size-dimorphic polygynous species (18). However, selective hunter-induced mortality can also be markedly sex-biased in weakly dimorphic species depending on the hunter preferences and hunting regulations, if any (19,20).

Overhunting has increased in the last three decades partly due to widespread road expansion, more affordable transportation and hunting technology, population growth, higher market demand, and the use of more efficient hunting methods (20,21,22). In addition, all of these aggravating factors tend to be correlated with each other. For example, the contemporary use of shotguns in some traditional communities in Cameroon is related to the emergence of more specialized and highly efficient hunters with clear market orientation (24). A critical element of effective conservation is therefore a proper understanding of the range of motivations propelling hunting behaviour in humans (25).

A comprehensive list of typologies of illegal hunting motivations could include household consumption, commercial gain, recreational and trophy killings, protection of self and property, poaching as a traditional right, gamesmanship, disagreements with specific regulations and subversive poaching (26). Moreover, we can essentially distinguish hunting motivations into either intrinsic, when the demand for game kills arises from *in situ* concerns in which the hunters themselves are embedded, or

extrinsic, which arises from a motivation outside the community and often accounts for the largest fraction of the animal biomass offtake (27,28). For example, the perceived risk of livestock predation by wolves in Spain and jaguars in Brazil are key motivations for the systematic poisoning of these animals (29). In addition, wild meat (including terrestrial and aquatic vertebrates) represents the main source of protein in the daily diet of traditional populations worldwide (30), and is critical for the well-being and nutrition of local communities (31). Hunting intended for self-consumption also includes animals selected for therapeutic purposes (32), amounting to intrinsic motivations to hunt. On the other hand, commercial offtake of elephants and rhinos is primarily motivated by the exceptional market value of ivory and horns, respectively. Moreover, the illegal trade in bushmeat and other body-parts has led to many social and economic problems as local prices of commercially valuable species and their derivatives continue to rise, which in turn generates ever greater incentives for this illegal practice (33,34).

Individual-based motivations can become even more complex at sites experiencing episodes of socio-political upheaval, which can increase demand in meat and other body parts and effectively lift restrictions on game hunting. For example, during civil wars in low-governance countries (outside of land mine zones) hunters become more encouraged to hunt due to sociopolitical changes induced by the uprise of conflicts (e.g. suspension of anti-poaching patrols, increased access to automatic weapons, and growing demand in urban markets). Increased market demand can be fueled by diverse sets of consumers, including rural war refugees, urban elites, and the international gourmet meat market. In addition, high-value animal by-products from the

illegal wildlife trade (such as elephant ivory and rhino horns) are often exchanged to finance militias. During the post-war period, however, large mammal populations will continue to decline unless there is a concerted cooperative effort to protect natural ecosystems and their wildlife (14,35). Regulation of high-value trade in threatened species also requires coordinated efforts to manage procurement. Few studies have focused on these intrinsic or extrinsic motivations underlying hunting and/or the mechanistic motivations underpinning game pursuit and choice, and ultimately why some mammal populations are more impacted than others.

Here, we examine the patterns of hunter motivation in a post-war zone in relation to species body mass and trophic level, including capture rates of individuals in different sex and age classes. Considering that conservation problems are inextricably linked to socioeconomic context, we also examine the degree to which the illegal wildlife trade has been more profitable in the short term than alternative livelihood activities pursued by local residents. To do so, we conducted interviews using a robust sample size of local hunters within the two most important protected areas of Angola, Southwest Africa. Finally, we show that different forms of motivation bias hunting selectivity in terms of species body mass, trophic level, and availability and further discuss the economic drivers behind hunting practices.

2 MATERIAL AND METHODS

2.1 Study Landscape

This study was carried out at Quiçama National Park and Quiçama Game Reserve, which span a total area of 960,000 ha (9°09'–10°23'S and 13°09'–14°08'E) and represent the most important conservation areas of Angola (36). We selected eight human settlements distributed throughout this area (Fig. 1). A government census showed that there were 25,086 residents in the Quiçama municipal county in 2014. Although this local population is not legally permitted to occupy these two protected areas, they have done so for generations, which creates tensions between residents and the reserve management. The semi-subsistence economy of the households in the area is sustained by hunting, small-scale fisheries, slash-and-burn cassava agriculture, and non-timber forest products. In early 2000, a rehabilitation project (Operation Noah’s Ark) conducted a species translocation program to the Special Conservation Area, but unfortunately many of these species were exotic to the park (37). These introduced and reintroduced alien species are not considered in this study.

Figure 1. Location of Quiçama National Park (demarcated by the blue line) and Quiçama Game Reserve (red line) in Southwest Africa. Solid yellow triangles indicate the surveyed townships, which encompassed more than one human settlement. Triangles sizes are proportional to the population size of the townships. Green and brownish background areas represent forest and savannah environments, respectively. Map generated using ArcGIS 10.3.1; Datum: WGS84. Elaborated by Ana Caroline Imbelloni; Franciany Braga-Pereira in August/2021.

2.2 Ethics approval

This research was approved by the i) National Health Council (Resolution 466/12), through the Research Ethics Committee of the Universidade Federal da Paraíba (license number 59846816.3.0000.5188); ii) Environmental Ministry of Angola (148INBAC.MINAMB/2016); iii) municipal administration of Quiçama (license number 017/GAB.ADM.MQ/2017); iv) leaderships of all sampled villages. An informed consent form was given to the interviewee, stating the purpose of the interview and ensuring the interviewee's anonymity, so each interview could not begin until after this form had been signed.

2.3 Data collection

We used Local Ecological Knowledge (LEK) to obtain information on hunting and wildlife use. The first contact between FBP and community dwellers occurred in 2014 to delineate the study area and present proposals to local villagers prior to deployment of this study. This facilitated access to the study area and a relationship of trust with local informants, including dedicated and semi-professional hunters. Data for this study were obtained from January 2014 to January 2015 and from January to April 2017. Translators were not required because both the interviewer and local informants spoke Portuguese. Interviews were conducted with 115 experienced local hunters (112 men and 3 women), who ranged in age from 19 to 80 years old. These hunters were selected using a snowball sampling technique (38), and on average we interviewed 14 informants per settlement. In addition to engaging in hunting activities, interviewees

also occasionally worked as small-scale farmers, fisherfolk, teachers and community leaders.

Field data were collected through semi-structured individual interviews (39) with the help of a visual stimulus checklist using colour photos of mammals living within the research area. For each game species reported as killed by the interviewed hunter, he/ she was asked to provide information on the (i) the date of the most recent mammal specimen killing; (ii) sex and age class of the most commonly hunted individuals; (iii) motivation behind the pursuit each wild mammals ; and (iv) the revenue acquisition from the sale of the animal when traded including the trade value (per kg) as: a) live whole animal, b) whole dead animal (e.g. fresh meat), c) processed as dried meat, d) processed skin, and e) ivory. We were therefore able to quantify the total aggregate value of each prey item. Each hunter was also asked about sources and amounts of income acquired from wage labour and other activities he or she may have performed, including farming, teaching and community leadership (see the complete questionnaire in Supplementary Material).

2.4 Data compilation

As all questionnaire answers were open-ended, in order to facilitate data analysis different answers to the question "what is the motivation behind the pursuit [this] species?" were classified into common themes following Braun and Clarke (40). Coded responses were summarized, thereby resulting in five hunting motivation categories: meat trade, pet trade, other trade in body parts, subsistence needs, and conflict

retaliation. This method of open-ended questioning provided the flexibility to explore, whenever necessary, different lateral topics of relevance while providing rapid anthropological assessments valid for wildlife research. Finally, we organized a binary data frame structured as species and interviewees in rows and hunting motivations in columns, in which the absence or presence of a specific motivation associated with a specific species, according to each interviewee, was attributed the values 0 or 1, respectively.

We used the PanTHERIA database (41) to obtain information on body mass and trophic level of each species. Based on specific morpho-ecological traits, all mammal species were assigned to seven dietary functional groups. We initially ranked the energetic stratum of the modal dietary pattern of each species as follows: (1) folivore < (2) frugivore < (3) granivore < (4) insectivore < (5) myrmecophage < (6) mesocarnivore < (7) hypercarnivore. We then weighted the proportion of each major dietary mode of any given species (sourced from (42,43) given these food energy levels (e.g. if the grazer/frugivore *Tragelaphus oryx* consumes 70% foliage and 30% fruit, its mean trophic energy level would be 1.3 (= (0.7 × 1) + (0.3 × 2)). We used the PanTHERIA and AnAge databases (44) to obtain information on species litter size and number of litters per year. Per capita annual fecundity (defined as female young per adult female per year) were therefore calculated as (litter size × number of litters per year)/2 (i.e. assuming a 50:50 birth sex ratio)(45). The monetary value of each animal is expressed as the mean transaction value per individual reported for each species by all independent interviewees. For details of English and scientific species name, and

species body mass annual fecundity rate, trophic level and monetary value acquired per animal see Supplementary material, Table 1.

We assigned the 8 sampled communities into four study sites according to type of landscape structure and geographic position. We therefore sampled two communities in savannah areas of northern Quiçama; two in savannah areas of southern Quiçama; two in central forest areas; and two communities in southern forest areas. Some differences between these sites include: the northern savannah communities are near (~20 km) a semi-urbanized area, and influenced by a nearby paved road. The central/southern forest sites had access to the highest large mammal abundance in Quiçama and access roads were in poor condition. The southern forest sites are near lakes and small streams, so the local population could also meet their protein needs from fish. The southern savannah site, although far from an urban center, had access to a road in good condition, thereby facilitation trade. There were some lakes and small rivers at this site, which could also supply some animal protein from fish.

2.4 Data analysis

Species traits associated to each hunting motivation

To examine the effects of body mass and trophic level on the number of hunters reporting each hunting motivation for killing any given species we performed generalized linear mixed models (GLMMs). We performed five different models associated with each hunting motivation. We considered i) the hunting motivation as a

response variable; ii) species body mass and trophic level as fixed effects predictor variables; and iii) interviewee as a random factor. Given that this is a binary response (0 or 1), we used binomial distribution. There was no observed collinearity ($p > 0.05$) among predictor variables. We used residual checks to verify whether our models were, in principle, suitable or otherwise. We used the Akaike information criterion to select models of interest if ΔAIC values >6 (ΔAIC obtain from the difference between a null and complete model AIC values) (46).

To test whether different hunting motivations, and species body size and trophic level were related to each other, we performed multiple factor analysis (MFA) (47). Compared to the frequently used principal component analysis (PCA), MFA takes into account the fact that data are structured into groups (here different species) to balance the importance of each group in the analysis. Species traits then become more important when any given species is repeatedly cited by several interviewees.

Local revenues from wildlife offtake

We performed linear models (LM) to examine the strength and direction of linear relationships between prey body size and fecundity rate; body size and local revenues accrued from sales of each species; and fecundity rate and local revenues. Given that only one predictor variable was explored in each LM, we used frequentist statistics to assess variable effects, including F-values, degrees of freedom, p-values and adjusted R^2-values.

Hunter demography associated with hunting motivations

To examine the effects of age, gender and natal community on the number of hunters reporting killing motivations for different species, we performed generalized linear models (GLMs). We structured five different models associated with each hunting motivation. We considered i) hunting motivation as a response variable; and ii) age, gender, and natal community of each interviewee as predictor variables, and used a binomial distribution to model the binary responses. We used residual checks to verify whether our models were, in principle, suitable or otherwise. We used the Akaike information criterion to select models of interest if ΔAIC values >6 (ΔAIC obtain from the difference between a null and complete model AIC values) (46).

These GLMs ensured we could assess which catchment area were associated with a higher number species hunted by a larger number of hunters according to each hunting motivation. MFAs were further performed to examine the degree to which hunting motivations were the same across all game animal age and sex classes, and hunting sites.

All analyses were performed in R ver. 3.5.3 (R Core Team 2019). GLMMs and GLMs were based on the *lme4* (48) package and MFAs were based on the *FactoMineR* (47) package.

3 RESULTS

3.1 Prevalence and history of kills and game choice

Species reported as captured by all interviewed hunters were Bushbuck Kewel and Bushpig. Blue Monkey and Blue Duiker were also killed by 94.7% and 93.0% of all interviewed hunters, respectively. Species killed by a small number of hunters included Hippopotamus (16.5%) and Elephant (10.4%). Most of the recent capture records for small and medium-sized species had occurred within the previous 10 years, with few exceptions such as Serval, Wild dog and Hyena. These former species followed the pattern of large mammals, for which the most recent captures occurred within 20 to 50 years prior to interviews (Fig. 2). Adult animals were far more frequently selected, as reported by 89.6% of all informants, with the remainder indicating that animals were either selected regardless of age class (8.0%) or that young animals were more frequently selected (2.4%). In relation to sex classes, most informants (54.9%) reported that there were no differences in the frequency with which either males or females were selected, although 39.5% indicated that males were mainly targeted, with only 5.6% of all informants indicating that females were more frequently selected.

Figure 2. Records of the most recent year in which each interviewee had killed a mammal species*.* Data are presented in 10-year increments for historical (1967-2016) and modern periods (2017). Response rates were calculate from the number of hunters reporting killing each species. Species are denoted by their English names and ordered top to bottom from the smallest to the largest species.

3.2 Motivations driving prey acquisition

From those hunters reporting intentionally capturing each species, the main intrinsic motivations were subsistence meat acquisition and retaliation against wildlife posing perceived threats; while extrinsic motivations were bushmeat trade, and trade in animal body-parts and pets. With the exception of Greater Galago, all other game species were captured to attend two or more motivations and seven species to attend for out of the five motivations (Common Duiker, Bushpig, Blue Monkey, Cape Porcupine, Malbrouck, Greater Cane Rat and Talapoin) (Supplementary Material, Fig 1). Some hunters also used some animal body parts for zootherapeutical purposes, but this was rarely the primary motivation for killing any given species. All respondents reported killing at least one species for food, retaliation and sales of meat. However, trade in body parts was restricted to only 40% of all informants.

Species hunted to supply both subsistence needs and the meat trade were trophically similar and primarily herbivores at low dietary energy levels. . The first dimension (Dim_1) of the MFA accounted for 25.9% of the variability in hunting motivation across different species (Fig. 3a). The meat trade, subsistence consumption and dietary level contributed 34%, 32% and 25% of the variance explained by Dim_1, respectively, whereas procurement for other body parts, the pet trade, retaliation against "problem" species and prey body size combined contributed with only 10% (Fig. 3b). The second MFA dimension (Dim_2) explained 19.6% of the variance with retaliation and body size explaining most of the variance (37% and 32%, respectively), whereas other factors were relatively unimportant (pet trade 14%, trade in body-parts 10%, subsistence needs 5%, dietary level 2% and the meat trade 2%; Fig. 3c).

Figure 3. Species compositional similarity in terms of hunting motivations obtained from (a) a multiple factor analysis (MFA). Contributions of each hunting motivation and species trait for (b) the first (Dim_1) and (c) the second dimension (Dim_2). The red dotted line indicates the percentage that would be obtained if all factors contributed equally to the overall variance.

Local meat consumption was associated with the killing of species at low trophic levels ($p< 0.001$), yet harvested prey spanned the entire spectrum of body mass, from Cane Rats (4 kg) to Bushpigs (69 kg). Killings justified as retaliation against human-wildlife conflicts involved mainly smaller-bodied species ($p<0.01$) across all trophic levels, for reasons including crop-raiding (e.g. primates such as Blue Monkeys and Malbrouck Monkeys) and predation on domestic livestock, fish or wild animals of human interest (e.g. felid depredation on chickens). For the bushmeat trade, species at low trophic levels were cited as significantly positively selected ($p< 0.001$), particularly large-bodied species. Kills to fuel the trade in animal body parts included mainly felids ($p< 0.01$) of varying sizes and intentional captures of primates were also motivated by the pet trade (Fig. 4; Table 1). Animal products were often sold to intermediaries who, in turn, traded at higher profits outside local communities. Although informants were unsure about reporting the ultimate motivation behind felid skeletal parts traded, they believed those would be used largely for ornamental or medicinal purposes either within Angola or other countries. In addition to Carnivores, other prey species provided body

parts that were secondarily used for medicinal purposes by local communities. However, Honey Badgers (*Mellivora capensis*) were reportedly killed primarily for therapeutic purposes (Table 2).

Figure 4. Species selected to meet different types of hunting motivations. Response rates were calculate from all interviewed hunters (n=115). Species are denoted by their English names and ordered left to right from smallest to largest. Circle sizes are proportional to species body mass; circle colours denote trophic level in terms of dietary energy, ranging from green (lowest) to red (highest).

Table 1. Details of the complete and null model using generalized linear mixed models (GLMMs) to examine the effects of body size and dietary level on the number of hunters reporting each form of hunting motivation for killing any given species. Estimated values indicate the coefficients associated with the variable listed on the left. This represents the estimated amount by which the odds (that each response variable would increase if each explanatory variable were one unit higher). Z-values indicate the degree to which explanatory variables exert a significant effect. Pr (>|z) denote significance levels as following: ns P > 0.05; ** P ≤ 0.01; *** P ≤ 0.001.

Response variable	Predictors	Estimate	Std. Error	z value	Pr(>|z|)		AIC	AIC Null model	ΔAIC
Subsistence meat	Body mass	-1.74E-06	6.96E-07	-2.502	0.0124	*	2604.5	3117.1	512.6
	Dietary level	-7.63E-01	5.18E-02	-14.755	<2e-16	***			
Wild meat trade	Body mass	-4.49E-04	6.97E-04	-0.644	0.519		2846	3260.7	414.7
	Dietary level	-7.29E-01	4.93E-02	-14.79	<2e-16	***			
Trade in other body parts	Body mass	2.94E-04	7.03E-04	0	0.675		1006.6	1113.9	107.3
	Dietary level	4.11E-02	5.11E+01	10.590	<2e-16	***			
Pet trade	Body mass	-8.90E-02	1.40E-03	-63.776	<2e-16	***	376	479	103
	Dietary level	-1.257	4.18E-01	-3.000	0.0027	**			
Retaliation	Body mass	-1.74E-06	6.96E-07	-2.502	0.0124	*	2604.5	3602.4	997.9
	Dietary level	-7.63E-01	5.18E-02	-14.755	<2e-16	***			

Table 2- Mammal species and their respective body parts used for medicinal and "magical" purposes within Quiçama National Park and Quiçama Game Reserve, Angola, Southwest Africa

Species/Vernacular name	Ailment treated	Animal body part used	IUCN status
Panthera leo - Lion	Asthma	Fat	VU
Panthera pardus - Leopard	Ward off envy	Skin	VU
Mellivora capensis - Honey badger	Physical weakness	Bone	LC
Tragelaphus scriptus - Bushbuck kewel	Personal animosity	Skin	LC

3.3 Local revenues from wildlife offtake vs other activities

Large-bodied species yielded the highest household revenues obtained from sales of single prey items ($F_{1,17}$ = 299.2; p< 0.001; R^2_{adj} = 0.94). To make matters worse, these large mammals often exhibit low fecundity rates (Fig. 5). The five most valuable prey species fetched an average market price per individual carcass of US$ 1359 (range = US$ 638 - 3117 per individual). These species, however, were becoming scarce and had not been recently captured by local hunters (Fig. 2).

Compared to revenues provided by medium and large-bodied species, local revenues accrued from any alternative wage labour option were very modest and well below the poverty line for Angola, where monthly income are US$98, US$43, and US$33 for teachers, farmers, and community leadership roles, respectively. In comparison, the value accrued from a single kill exceeding 50 kg was considerably greater. For example, annual revenues from gazelle kills (*T. scriptus)* alone were twice that from teaching, and the sale value of a single leopard skin was nearly four-fold

higher than the monthly wages of a primary school teacher, who earned a higher income than either a farmer or community leader.

Figure 5. Monetary values obtained from each prey item traded in the local market. Mammal species are ordered along different axes i) z: from top to bottom from least to most profitable; ii) x: from left to right from largest to smallest; and iii) y: from left to right from lowest to highest fecundity rate. Warmer colours indicate higher offtakes in monetary values. Dollar values were based on the 2017 Kwanza conversion rate (US$1 = 165 Kwanzas).

Hunter traits and hunting motivations

In relation to the sites (regarding landscape structure and geographic position), GLMs showed that a higher number of kill reports in northern savannah areas were due to subsistence and the meat trade than in either southern savannah and forest sites. Forest sites showed a higher number of prey offtake reports induced by retaliation killings and trade in other body parts compared to savannah sites (Supplementary Material, Table 2). This is consistent with the MFA analysis which showed a high overlap of species selected for the meat trade and subsistence in northern savannahs, and a similar set of species killed due to the body-part trade or conflict retaliation across all forest sites (Supplementary Material, Fig 2). Although middle-aged male hunters more frequently reported mammal kills, sex and age classes of informants had a low contribution in explaining MFA dimensions 1 and 2 (Supplementary Material, Fig 3).

Hunting for the wildlife trade was primarily done by adult men, whereas kills attributed to local crop-raiding or livestock depredation and local subsistence, were also carried out by adult women, children and the elderly.

4 DISCUSSION

In the early stage of the Angolan civil war, target mammal species harvested at the Quiçama National Park region of Angola consisted primarily of medium- to large-bodied species. This was consistent with the set of game species sold in the markets in Luanda (capital of Angola), which were commercially valuable as either bushmeat or other body parts. However, as large-bodied mammals became increasingly overhunted and their populations commensurately depleted, the size structure of prey species gradually shifted towards small to medium bodied species (14). This explains why recent mammal captures are largely comprised of small to medium-sized species. In addition, even if the most important motivation associated with kills of large-bodied species was the wildmeat trade, medium and small-bodied species were also captured to supply the bushmeat market. These species were also often captured for other reasons, such as subsistence needs and retaliation against wildlife posing threats.

Given the temptation to participate in the bushmeat trade, some informants explained that they were prepared to invest several days searching more remote areas of Quiçama in pursuit of large-bodied mammals even if their numbers had been depleted. This is consistent with a study in Indonesia, where market demand and supply of wild pigs was unaffected when local wild pig populations collapsed because

hunters simply travelled farther in pursuit of game (49). This hunter-prey system must, however, be carefully evaluated because despite the great effort by some hunters to capture species providing high energy and monetary returns, several species are absent from prey profiles in the recent years, which likely indicates severe population declines if not local extinctions. Among the five largest and most profitable species, red buffalos were recently slaughtered, but their remaining populations are now restricted to remote forest areas in the southern Quiçama region. Those buffalo populations, therefore, should receive immediate conservation attention, to prevent regional scale extirpation such as the case of native populations of Eland and Roan Antelope.

Intensification of hunting activities over the last decade can be understood given the dire post-war economic crisis in Angola, which is now further exacerbated by the COVID-19 outbreak that is plaguing the country. As a consequence, hunters from Northern Quiçama are hunting in the proximity or within the special protection zone of QNP, which corresponds to 10% of the park area that is patrolled on a daily basis by 15 rangers. This area and even the most remote portions of Quiçama have become increasingly accessible to not only local residents, but also outsiders in search of desirable game and other nontimber products. In a plight similar to that of red buffalos, native elephant populations at Quiçama are now restricted to forest landscapes where they are less detectable (14). Elephant populations in Angola as a whole are experiencing intense poaching pressure as well as losing habitat to expanding human settlements. Based on observed carcass ratios, a study has shown that elephant populations in southern Angola started to increase after the war until 2005, but then

began to decrease at an annual rate >2% (50)(Schlossberg et al 2019). Although many landmines remain in southeast Angola following the civil war, and elephants have been killed by mines (51) poaching for ivory trade, rather than landmines, is the primary cause of mortality based on a large number of carcasses observed in a survey conducted in 2015 in southern Angola(50). As a consequence, Angola is in the midst of one of the worst poaching crises of any country within the savannah range of African elephants and nationwide population declines will almost certainly continue if the drivers of mortality are not reversed.

Hunters reported that they often captured more than one individual larger than 40 kg each month to maximize income from bushmeat sales, but that those species often failed to contribute meat to their household subsistence for several months. In this case, only less desirable leftovers from dressed carcasses of large animals, such as the head and hooves, would be locally consumed, with consumption of whole prey items of large mammals in the hunter's household restricted to the most important holidays. This partially explains why body mass did not exert a significant effect on species selectivity in terms of community-level subsistence, given that more frequent local consumption of smaller-bodied species can be explained by both the higher abundance of those species and the fact that larger species were primarily directed to the bushmeat trade. However, another important factor to be considered is overall preference in terms of palatability. For example, interviewees reported that medium-sized mammals such as Blue Duiker (5 kg) have "soft tasting" meat compared to several larger species such as Red Buffalo (600 kg). Also, villagers typically complied

with some important dietary taboos regarding primates, which were rarely consumed at Quiçama even though they were one of the most abundant mammal taxa larger than 1 kg.

Primates at Quiçama were killed primarily in retaliation against crop raiding and livestock depredation and to supply the pet trade, with exception of Blue Monkeys (6 kg), reported by many hunters as killed to fuel the bushmeat trade. Although primates were rarely used as a source of meat across the study area, this practice is common in other African and Neotropical sites (52), and they account for the largest number of mammal species (126) threatened by hunting worldwide (5). Given plausible scenarios of wildlife depletion, however, hunters in the future may become less selective and gradually change their hunting practices to include primates as game species, which could be directed to either the bushmeat trade or household subsistence (14). Local use of mammals for medicinal purposes did not necessarily involve new kills since the animal parts used were essentially minor byproducts of prey acquisition to meet other purposes. In addition, there are cases in which the medicinal exploitation of wild mammals involved the non-invasive use of scats or dung, rather than actual mortality (32).

In Quiçama, the median abundance value of all large and medium-sized game species, and some small-bodied game species (Blue Duiker and Blue Monkey), in savannah areas declined during the war and did not recover during the post-war period. In contrast, in forest landscapes, 15.8% of all large and medium-sized game species, whose abundance had declined during the war, have since experienced post-war

population recovery (14). This helps explain why hunting for both for subsistence and meat trade encompassed a larger number of species in the northern savannahs than in the forest sites; since savannah areas are the most defaunated, prey acquisition in this environment follows a generalist hunting strategy that goes well beyond selection of preferred species. On the other hand, hunters in the southern savannah areas had access to alternative fish protein, which was either consumed locally or sold. Hunters at these sites would thus also go fishing but concentrate on the most desirable game species when hunting. In terms of non-food motivations, the number of reports of species killed for trade in other animal body parts and retaliation was higher in forest than in savannah areas, because forest sites hosted the highest abundance of species whose body parts are traded (such as leopard skin and elephant ivory); and crops and domestic livestock were nearer a wider spectrum of crop-raiders and larger carnivores. In recent years, residents of forest sites in the Center-South of Quiçama have asked the local government for support because elephants that emigrated from the region during the war years, are currently returning to their habitat and raiding local crops.

We found that species at the lowest dietary trophic levels were the most frequently captured for both the wild meat trade and local subsistence. This is consistent with data from kill profiles in Afrotropical forests showing that bushmeat harvest rates of frugivore–herbivores are the highest, both in terms of the number of carcasses (44.5%) and overall biomass (57.2%) (53). However, carnivores were frequently involved in human-wildlife conflicts and were also affected by commercial use of body parts for both medicinal (bones and fat) and ornamental (skin and teeth)

purposes. Many leopards were incidentally caught in traps set for antelopes, and then subsequently killed for the illegal wildlife trade. Given that carnivore skin prices are based on quality which is reduced by bullet perforations, some hunters at Quiçama either hanged the animal or waited for it to die from bleeding, rather than immediately killing the animal. Protecting hide quality from projectile damage also explains why many carnivores were poisoned to death rather than shot(54).

Considering the hunting motivations described here (subsistence, animal trade and conflict retaliation), similar motivations were found in a study carried out in Tanzania, which showed that food acquisition and generation of income (mentioned by 79% and 78% of all interviewees, respectively) were most frequently cited as to why hunters poached(28). A review on hunting motivations in Vietnam also showed that crop protection and cash income were the main motivations behind hunting (55). On the other hand, a survey conducted in Oregon, USA, showed that recreational and trophy hunting were the main motivations driving deer hunting (56). These differences can be partly explained by the low-income populations of villagers at Quiçama who are unlikely to go hunting unless this can generate either direct or indirect income. Our results also show positive hunter selectivity targeting adults, which we attributed to their larger size and greater profits from sales. Likewise, a global survey revealed that humans kill adult males and females, the reproductive capital of prey populations, up to 14 times more often than natural terrestrial predators, such as wolves or lions (2). As a consequence, life-history traits may often shift to early reproduction since older animals are consistently eliminated by hunters(57). We found that this also applied to males,

which were generally larger and provided higher returns than females. In addition, interviewees indicated that males were more frequently captured because they are more "distracted" and consequently failed to detect active traps. Some studies have shown that such selection for adults of a single sex may lead higher reproductive investments (58,59). Additionally, due to the logic dictating that larger animals are more frequently selected in both subsistence and commercial exploitation systems (20), phenotypic changes of populations selected by humans show a 20% decline in traits related to size and 25% in traits associated with reproductive stage. These values are higher than those observed in societies in which hunters select prey only on the basis of local consumption (59).

In relation to division of labour and social norms, there was a male-bias in hunters involved in the bushmeat trade, whereas women primarily pursued smaller-bodied species for either local consumption or protecting livestock. Among modern hunter-gatherers, female exclusion from hunting large mammals is apparently closely related to the manufacture and use of traditional or fire weapons, and associated economic and/or religious norms (60). Women often have no hunting weapons of their own so their hunting activities under those restrictions are confined to small-bodied prey. A number of anthropologists agree that the division of labour among such groups stems from culturally derived, gender-based beliefs, the importance attributed to big-game hunting, and the convertibility of these into prestige and authority that preclude female access to essential weapons, rather than fundamental sex-based anatomical or physiological differences. Male-biased hunting effort allocates to men society's most

valued labour and opportunities to distribute high-value foods. Division of labour in relation to prey acquisition is thus grounded in gender politics rather than inherent sex differences (60).

Regarding the motivations across different sites, the roads that traverse savannah habitats in Northern to Southern Quiçama serves as an important route for the wildlife traffic. The greater difficulty in accessing and navigating forest areas has rendered hunting activities therein less intensive than in savannah areas (14), but we found that established traffic routes have connected cities like Muxima and Dondo to forest refugia serving as the last stronghold for buffalo and large carnivore populations. Another threat is the marked growth of urban development and extractive activities in Quiçama coastal areas. The detrimental effects of human incursion into Angolan protected areas on the abundance and distribution of large mammal species underscore the need for intensive mitigation of anthropogenic threats to restore the high wildlife conservation value in all areas impacted by the civil war (61). Also, the problem is collective, given that hunting is carried out by both residents and outsiders, often motivated by urban commerce and illegal traders of ammunition and fire weapons. Some of the arsenal typically used by hunters at Quiçama consisted of new ammunition and automatic rifles, which indicate that they were sourced from military bases within the park, which were installed during the Angolan civil war.

Our results show that hunting is considerably more profitable in the short term compared to alternative sources of local income. It also pays to go hunting because enforcement against (and penalties related to) poaching are virtually non-existent and

people cannot, and will not, protect species and natural habitats as a long-term strategy if their short-term needs are not met (28,62). However, these benefits are modest, largely unsustainable and come at a high price. They are modest because local hunters receive only the smallest share of the profits from the illegal wildlife supply chain, yet this follows a logic rationale because alternative opportunities to derive benefits from wildlife are unavailable. For example, although the income from a Cape Bushbuck sale was on average US$41 (24000kz) for a local hunter, the profits associated with selling this animal to an urban restaurant could reach US$100 (36000kz). The price discrepancy of ivory per kilogram was even greater, with local hunters earning less than US$50/kg of ivory, whereas this can fetch US$2150/kg in international markets despite the fact that ivory prices have plummeted since 2014(63). Needless to say, the ivory trade is largely unsustainable and comes at a high price in several African countries, with the value generated from the ivory trade corresponding to only 20% (US$85/km^2) of the value generated from ecotourism(64,65). A loss of up to US$41.1 million in potential annual income has therefore been estimated for some countries as consequence of bushmeat hunting. Hence, poaching should not be seen as a solution to the lack of alternative livelihoods because its food security benefits are generally unsustainable and often brings fewer economic and livelihood benefits compared to ecotourism and trophy hunting (64,65).

The traditional population of Quiçama remains disenfranchised, in terms of healthcare, education, income opportunities and other rights. The lack of institutional capacity to effectively mitigate anthropogenic threats to both biodiversity and traditional

communities at Quiçama is often justified by many as an ongoing consequence of the civil war, but there are examples that wildlife populations can recover through protection, and local residents can be ethically assisted even in the aftermath of the war. For example, after peace was declared, the Mozambique government partnered with a Foundation to restore Gorongosa National Park. Those living around parks received assistance for farming, education and healthcare and several hundreds also found jobs in the park(66). A persistent absence of institutional enforcement in countering threats to wildlife could thus be a lingering effect of sustained armed conflicts, but it should not always be justified as an obstacle to nature conservation. Although the mammal fauna of Quiçama is exposed to mounting perils, such as human population growth, deforestation and spread of exotic species, bushmeat exploitation for commerce is undoubtedly the most imperative threat. We therefore argue for the imminent implementation of activities that can ensure both wildlife recovery, and food security and egalitarian rights for the local human communities. These efforts should include environmental education, greater human rights and social equality, and the understanding and integration of legal anti-poaching instruments and hunting management. Given the frequent engagement of residents with hunting and the highly scenic landscape value of the study area, we strongly encourage community-based ecotourism and wildlife management activities. In addition, considering that local ecological knowledge can efficiently strengthen management and conservation guidelines, we also highlight the importance of employing local villagers as wildlife guides and including them in official wildlife management and monitoring plans. We

also draw attention to the need to implement adequate anti-poaching enforcement and full-time presence of park staff in the central and southern portions of Quiçama, especially in areas exposed to the most intensive hunting activity and along roads through which the wildlife trade operates. Finally, the routes that link Quiçama to large cities must be monitored and transformed from traffic routes into highways that enable sustainable activities that capitalize on the regional ecological and cultural diversity, such as educational endeavors for school kids and ecotourism.

Acknowledgements

We sincerely thank the people from local communities who shared their knowledge on wildlife and supported us in data collection through opportunist observations. Without them, this work would not be possible. We would like to thank the Environmental Ministry of Angola, Quiçama municipal administration, Quiçama Park administration and all village coordinators for authorizing and support this research. We thank the Faculdade de Ciências of Universidade de Agostinho Neto (Angola) and The Rufford Foundation for the financial support. F-BP thanks the Coordenação de Aperfeiçoamento Pessoal de Nível Superior (CAPES) for the master scholarship. RRNA thanks National Council for Scientific and Technological Development by a productivity research grant. We thank the reviewer for the important contribution to this manuscript.

REFERENCES

1. Bordes, F., Thibault C. Thoughts on the initial adaptation of Hominids to European glacial climates. Quat Res [Internet]. 1997; Available from: https://doi.org/10.1016/0033-5894(77)90059-X
2. Darimont, C.T., Fox, C.H., Bryan, H.M., Reimchen TE. The unique ecology of human predators. Science (80-) [Internet]. 2015; Available from: https://doi.org/10.1126/science.aac4249
3. Alves, R.R.N., Silva, J.S., Chaves, L.S., Albuquerque UP. Ethnozoology and Animal Conservation. In: Alves, R.R.N., Albuquerque UP, editor. Ethnozoology: animals in our lives. Academic Press. Elsevier, London,; 2018. p. 481–496.
4. Peres, C.A. Effects of hunting on western Amazonian primate communities. Biol Conserv. 1990;54(1):47–59.
5. Ripple WJ, Abernethy K, Betts MG, Chapron G, Dirzo R, Galetti M, et al. Bushmeat hunting and extinction risk to the world's mammals. R Soc Open Sci [Internet]. 2016;3(10):160498. Available from: http://rsos.royalsocietypublishing.org/lookup/doi/10.1098/rsos.160498
6. Benítez-López, A., Santini, L., Schipper, A. M., Busana, M., & Huijbregts MAJ. Intact but empty forests? Patterns of hunting-induced mammal defaunation in the tropics. PLOS Biol [Internet]. 2019;17(5). Available from: 10.1371/journal.pbio.3000247
7. Peres, C.A. and Lake IR. Extent of nontimber resource extraction in tropical forests: accessibility to game vertebrates by hunters in the Amazon basin. Conserv Biol. 2003;17(2):521–35.
8. Bogoni JA, Peres CA, Ferraz KMPMB. Extent, intensity and drivers of mammal defaunation: a continental-scale analysis across the Neotropics. Sci Rep [Internet]. 2020 Dec 15;10(1):14750. Available from: http://www.nature.com/articles/s41598-020-72010-w
9. Effiom, E.O., G. Nuñez-Iturri, H.G. Smith UO& OO. Bushmeat hunting changes regeneration of African rainforests. Proc R Soc B Biol Sci. 2013;280(1759):1–8.
10. Nasi, R., A. Taber and N van V. Empty forests, empty stomachs? Bushmeat and

livelihoods in the Congo and Amazon Basins. Int For Rev. 2011;13(3):355–68.

11. Leopold AS. Wildlife of Mexico: The Game Birds and Mammals. Berkeley and Los Angeles: Univ of California Press; 1959.

12. Redford, K.H., Robinson JG. The game of choice: patterns of indian and colonist hunting in the Neotropics. Am Anthr. 1987;89:650–667.

13. Cardillo M, Cardillo M, Mace GM, Jones KE, Bielby J, Bininda-Emonds ORP, et al. Multiple Causes of High Extinction Risk in Large Mammal Species. Science (80-) [Internet]. 2005;309(5738):1239–41. Available from: http://www.ncbi.nlm.nih.gov/pubmed/16037416%5Cnhttp://www.sciencemag.org/content/309/5738/1239%5Cnhttp://www.sciencemag.org.proxy.bibliotheques.uqam.ca:2048/content/309/5738/1239.abstract?ijkey=1c808d0dc7686f93d6eb0d6c1352429f2390e3d2&keytype2=tf_ipsecsha

14. Braga-Pereira F, Peres CA, Campos-Silva JV, Santos CV-D, Alves RRN. Warfare-induced mammal population declines in Southwestern Africa are mediated by species life history, habitat type and hunter preferences. Sci Rep. 2020;10(1):1–15.

15. Di Marco M, Boitani L, Mallon D, Hoffmann M, Iacucci A, Meijaard E, et al. A Retrospective evaluation of the global decline of carnivores and ungulates. Conserv Biol. 2014;28(4):1109–18.

16. Dirzo R, Young HS, Galetti M, Ceballos G, Isaac NJB, Collen B. Defaunation in the Antrhopocene. Science (80-). 2014;401(6195):401–6.

17. Davidson AD, Hamilton MJ, Boyer AG, Brown JH, Ceballos G. Multiple ecological pathways to extinction in mammals. Proc Natl Acad Sci [Internet]. 2009;106(26):10702–5. Available from: http://www.pnas.org/cgi/doi/10.1073/pnas.0901956106

18. Pac, D. F., & White GC. Survival and Cause-Specific Mortality of Male Mule Deer Under Different Hunting Regulations in the Bridger Mountains, Montana. J Wildl Manage. 2007;71(3):816–827.

19. Corlatti, L., Storch, I., Filli F and PA. Does selection on horn length in males and females differ between protected and hunted populations of a weakly dimorphic

ungulate? Ecol Evol. 2017;7:3713–3723.

20. Bugir, C.K.; Peres, C.A.; White, K.S.; Montgomery, R.A.; Griffin, A.S.; Rippon, P.; Clulow, J.; Hayward MW. Prey preferences of modern human hunter-gatherers. Food Webs. 2021;26.

21. Abernethy, K. A., Coad, L., Taylor, G., Lee, M. E., & Maisels F. Extent and ecological consequences of hunting in Central African rainforests in the twenty-first century. Philos Trans R Soc B Biol Sci. 2013;368(1625).

22. Bowler M, Beirne C, Tobler MW, Anderson M, Dipaola A, Fa JE, et al. LED flashlight technology facilitates wild meat extraction across the tropics. 2020;489–95.

23. Dudley JP, Ginsberg JR, Plumptre AJ, Hart J a, Campos LC. Effects of War and Civil Strife on Wildlife and Wildlife Habitats\nEfectos de la Guerra y Conflictos Civiles Sobre la Vida Silvestre y Sus Hábitats. Conserv Biol [Internet]. 2002;16(2):319–29. Available from: http://dx.doi.org/10.1046/j.1523-1739.2002.00306.x

24. Duda R. Hunting Techniques, Wildlife Offtake and Market Integration. A Perspective from Individual Variations among the Baka (Cameroon). The Center for African Area Studies, Kyoto University, Japan.; 2017.

25. Milner-Gulland EJ. Interactions between human behaviour and ecological systems. Philos Trans R Soc B Biol Sci. 2012;367(1586):270–278.

26. Muth, R. M., & Bowe JF. Illegal harvest of renewable natural resources in North America: Toward a typology of the motivations for poaching. Soc Nat Resour. 1998;11(1):9–24.

27. Bowen-Jones, E., Brown, D., & Robinson EJZ. Economic commodity or environmental crisis? An interdisciplinary approach to analysing the bushmeat trade in central and west Africa. Area. 2003;35(4):390–402.

28. Knapp EJ, Peace N BL. Poachers and Poverty: Assessing Objective and Subjective Measures of Poverty among Illegal Hunters Outside Ruaha National Park, Tanzania. Conserv Soc. 2017;15:24–32.

29. Mateo-Tomás, P., Olea, P. P., Sánchez-Barbudo, I. S., & Mateo R. Alleviating

human-wildlife conflicts: identifying the causes and mapping the risk of illegal poisoning of wild fauna. J Appl Ecol. 2012;49(2):376–385.

30. Bakarr M. OW and AE. West Africa: regional overview of the bushmeat crisis. In BCTF Collaborative Action Planning Meeting Proceedings. In: N. D. Bailey, H. E. Eves AS and JTS, editor. 2001. Silver Spring, MD;
31. Fa JE, Peres CA, Meeuwig J. Bushmeat Exploitation in Tropical Forests: an Intercontinental Comparison. Conserv Biol [Internet]. 2002 Feb;16(1):232–7. Available from: http://doi.wiley.com/10.1046/j.1523-1739.2002.00275.x
32. Braga-Pereira F, Santoro FR, Van-Duném Santos C, Alves RRN. First record of Angola's medicinal animals: A case study on the use of mammals in local medicine in quiçama national park. Indian J Tradit Knowl. 2017;16(4).
33. Wittemyer, G., Daballen, D. & Douglas-Hamilton I. Poaching prices: rising ivory prices threaten elephants. Nature. 2011;476:282–3.
34. Challender, D. W. S., & MacMillan DC. Poaching is more than an Enforcement Problem. Conserv Lett. 2014;7(5):484–494.
35. Pringle RM. Upgrading protected areas to conserve wild biodiversity. Nature. 2017;
36. Ministério do Urbanismo e Ambiente. Primeiro Relatório Nacional para a Conferência das Partes da Convenção da Diversidade Biológica [Internet]. 2006. Available from: https://www.cbd.int/doc/world/ao/ao-nr-01-pt.pdf
37. Liebenberg, A., Basterfield, P., Daehnke, A., Rogery GR. Report on the Elephants Relocated to the Quiçama National Park – Angola. University of Pretoria; 2001.
38. Bailey K. Methods of social research. 4th ed. The Free Press, New York; 1994.
39. H.R. B. Research methods in cultural anthropology. 1998. 200 p.
40. Braun V, Clarke V. Using thematic analysis in psychology. Qual Psychol Res. 2006;3(2):77–101.
41. JONES KE, BIELBY J, CARDILLO M, FRITZ SA, O'DELL J, ORME CDL, et al. PanTHERIA : a species-level database of life history , ecology , and geography of extant and recently extinct mammals. 2009;90(9).
42. Wilman, H., Belmaker, J., Simpson, J., de la Rosa, C., Rivadeneira, M. M., & Jetz

W. EltonTraits 1.0: Species-level foraging attributes of the world's birds and mammals. Ecology. 2014;95(7):2027–2027.

43. Bogoni, J. A., Silva, P. G. da, & Peres CA. Co- declining mammal- dung beetle faunas throughout the Atlantic Forest biome of South America. Ecography (Cop). 2019;

44. Tacutu, R., Thornton, D., Johnson, E., Budovsky, A., Barardo, D., Craig, T., Diana, E., Lehmann, G., Toren, D., Wang, J., Fraifeld, V. E., de Magalhaes JP. Human Ageing Genomic Resources: new and updated databases. Nucleic Acids Res. 2018;

45. ERNEST SKM. LIFE HISTORY CHARACTERISTICS OF PLACENTAL NONVOLANT MAMMALS Ecological. Ecol Soc Am. 2003;84(12):3402–2403.

46. Harrison, X. A., Donaldson, L., Correa-Cano, M. E., Evans, J., Fisher, D. N., Goodwin, C. E., ... & Inger R. A brief introduction to mixed-effects modelling and multi-model inference in ecology. PeerJ. 2018;6.

47. Husson F, Josse J, Le S, Mazet J. FactoMineR: multivariate exploratory data analysis and data mining. J Stat Softw [Internet]. 2018;25:1–18. Available from: https://rdrr.io/cran/FactoMineR/man/FactoMineR-package.html

48. Oksanen J, Blanchet F, Kindt R, Legendre P, Others, Other, et al. vegan:community ecology package. 2013.

49. Whitfield J. The law of the jungle. Nature. 2003;421(6918):8–9.

50. Schlossberg S, Chase MJ GC. Poaching and human encroachment reverse recovery of African savannah elephants in south-east Angola despite 14 years of peace. PLoS One. 2018;13(3).

51. AA B. The contribution of landmines to land degradation. L Degrad Dev. 2007;18:1–15.

52. Fa JE, Peres CA. Game vertebrate extraction in African and Neotropical forests: an intercontinental comparison. Conserv Exploit Species. 2001;203–41.

53. Fa, J. E., Ryan, S. F., & Bell DJ. Hunting vulnerability, ecological characteristics and harvest rates of bushmeat species in afrotropical forests. Biol Conserv. 2005;121(2):167–176.

54. Braga-Pereira F., Bogoni JA., Alves RRN. From spears to automatic rifles: The shift in hunting techniques as a mammal depletion driver during the Angolan civil war. Biol Conserv. 2020;249.

55. MacMillan, D.C. & Nguyen AQ. Factors influencing the illegal harvest of wildlife by trapping and snaring. 2013;

56. Shrestha, Suresh K.; Burns RC. Big game hunting practices, meanings, motivations and constraints: a survey of Oregon big game hunters. In: Fisher, Cherie LeBlanc; Watts, Clifton E. J, editor. Proceedings of the 2010 Northeastern Recreation Research Symposium. US; 2012. p. 1–8.

57. Milner, J.M., Nilsen, E.B., Amdreassen HP. Demographic side effects of selective hunting in ungulates and carnivores. Conserv Biol. 2007;

58. Milner-Gulland G. Sex-biased harvesting and population dynamics in ingulates: implications for conservation and sustainable use. Conserv Biol. 1994;

59. Darimont CT, Carlson SM, Kinnison MT, Paquet PC, Reimchen TE, Wilmers CC. Human predators outpace other agents of trait change in the wild. Proc Natl Acad Sci [Internet]. 2009;106(3):952–4. Available from: http://www.pnas.org/cgi/doi/10.1073/pnas.0809235106

60. Speth JD. Big-Game Hunting: Protein, Fat, or Politics? In: The Paleoanthropology and Archaeology of Big-Game Hunting Interdisciplinary Contributions to Archaeology. Springer, New York, NY; 2010.

61. Petracca, L. S.; Funston, P. J.; Henschel, P.; Cohen, J. B.; Maclennan, S.; Frair JL. Modeling community occupancy from line transect data: a case study with large mammals in post- war Angola. Anim Conserv. 2019;

62. Borgerhoff-Mulder M and PC. Conservation: linking ecology, economics, and culture. New Jersey Princet Univ Press. 2005;

63. Gao, Y., & Clark SG. Elephant ivory trade in China: Trends and drivers. Biol Conserv. 2014;180:23–30.

64. Lindsey, P.A., Balme, G., Becker, M., Begg. C., Bento, C., Bocchino, C., Dickman. A., Diggle, R.W., Eves, H., Henschel, P., Lewis . D., Marnewick , K., Mattheus, J., McNutt, W., McRobb, R., Midlane , N., Milanzi, J., Morley , R., Murphree , M.,

Opyene , V P. The bushmeat trade in African savannas: Impacts, drivers, and possible solutions. 2012.

65. Lindsey, P., Taylor, W.A., Nyirenda, V., Barnes L. Bushmeat, wildlife-based economies, food security and conservation: Insights into the ecological and social impacts of the bushmeat trade in African savannahs [Internet]. FAO. 2015. p. 58. Available from: FAO/Panthera/Zoological Society of London/SULi Report, Harare

66. Daskin JH, Pringle RM. Warfare and wildlife declines in Africa’s protected areas. Nature [Internet]. 2018;553(7688):328–32. Available from: http://dx.doi.org/10.1038/nature25194

Figure 1a

Click here to access/download;Figure;Figure1a.jpg

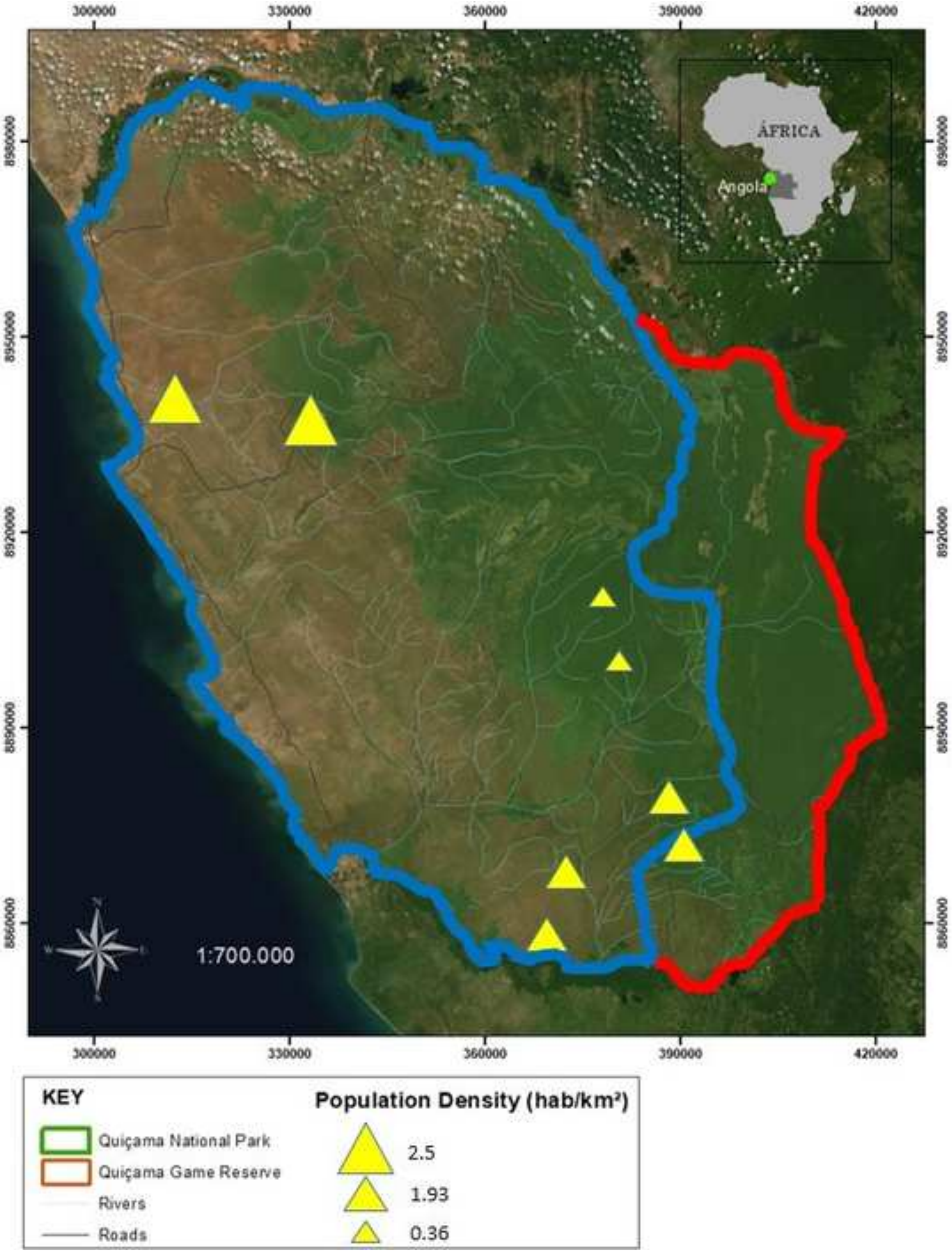

Figure 1b

Click here to access/download;Figure;Figure1b.jpg

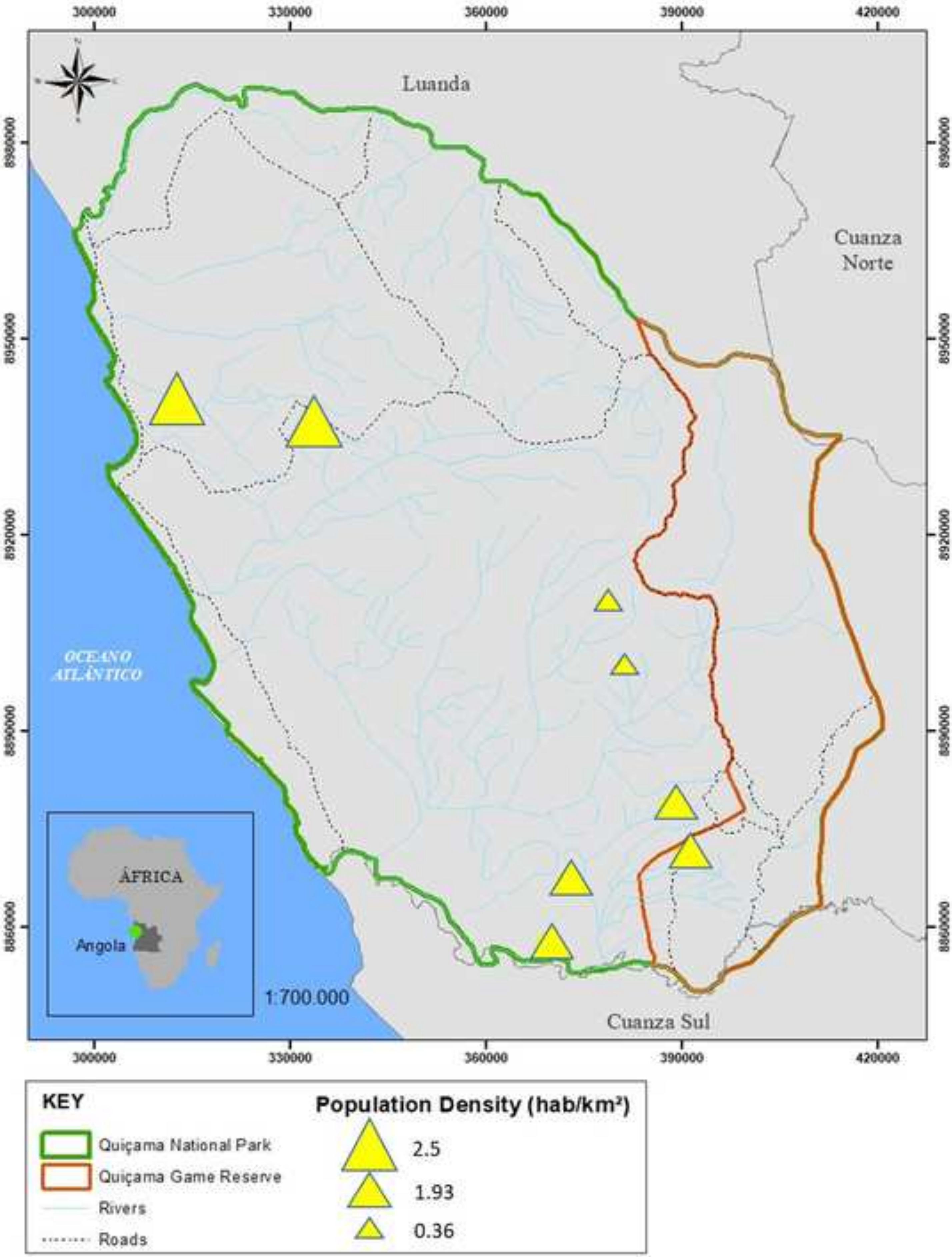

Figure 2

Click here to access/download;Figure;Figure 2.pdf

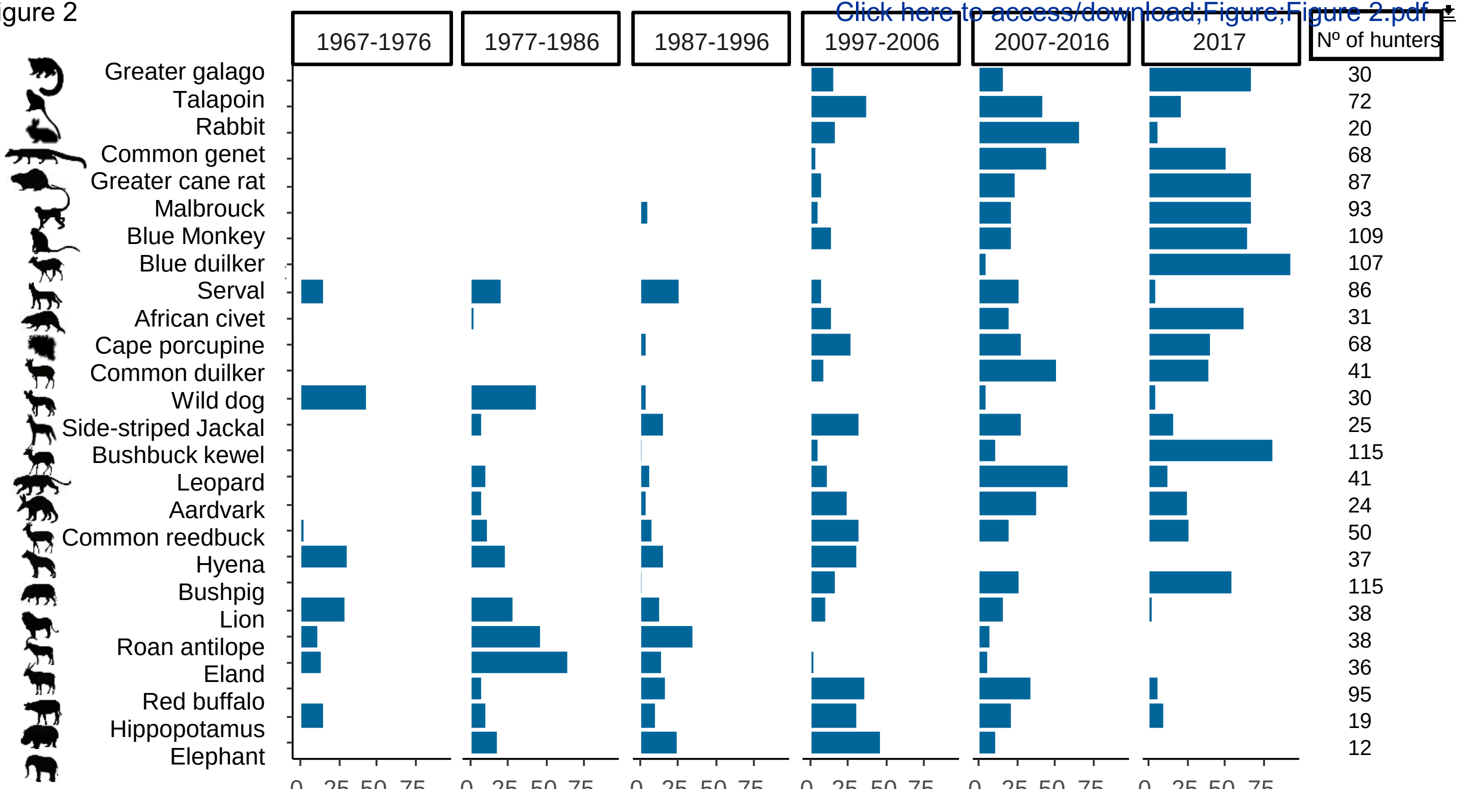

Figure 3

Click here to access/download;Figure;Figure 3.pdf

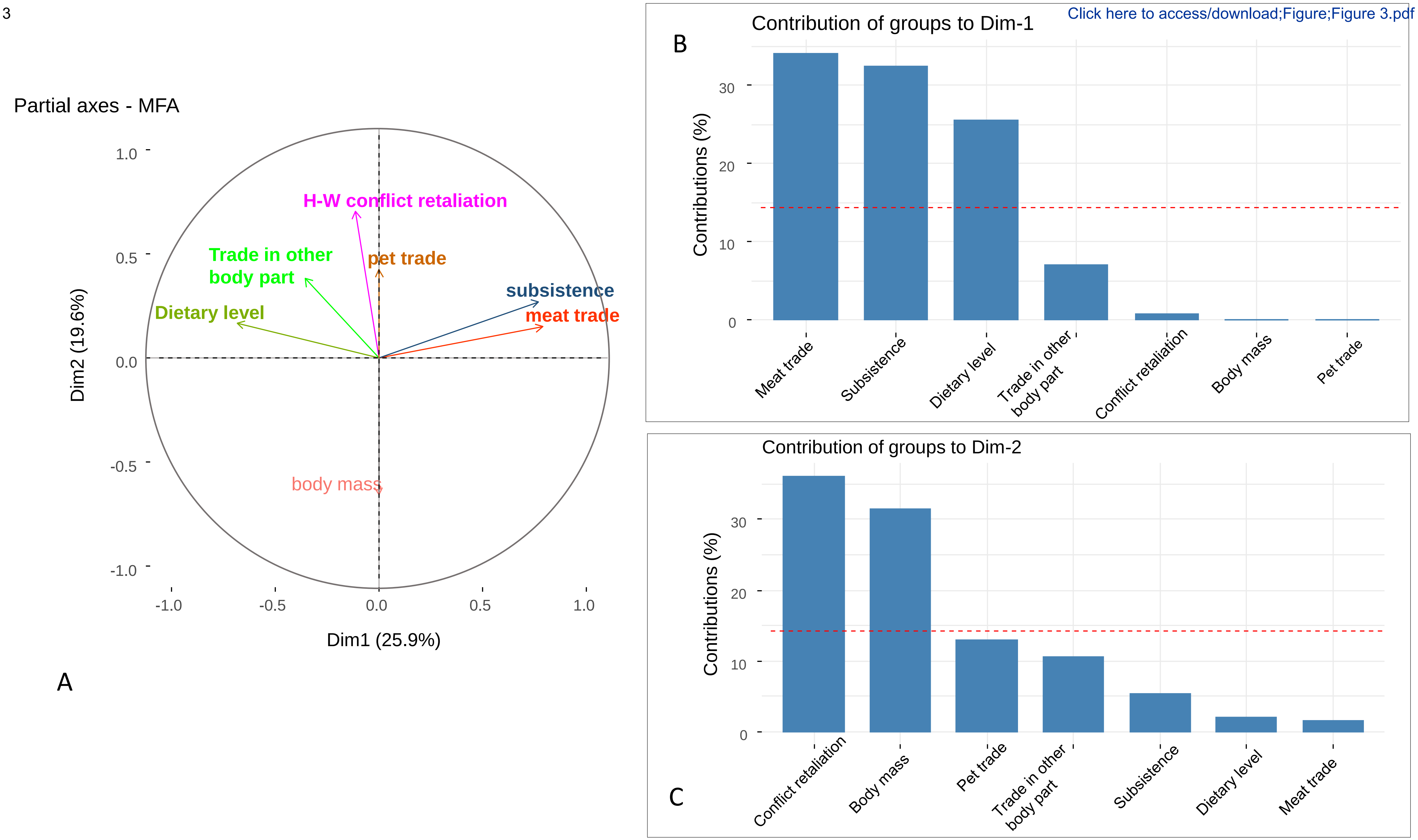

Figure 4

Click here to access/download;Figure;Figure 4.pdf

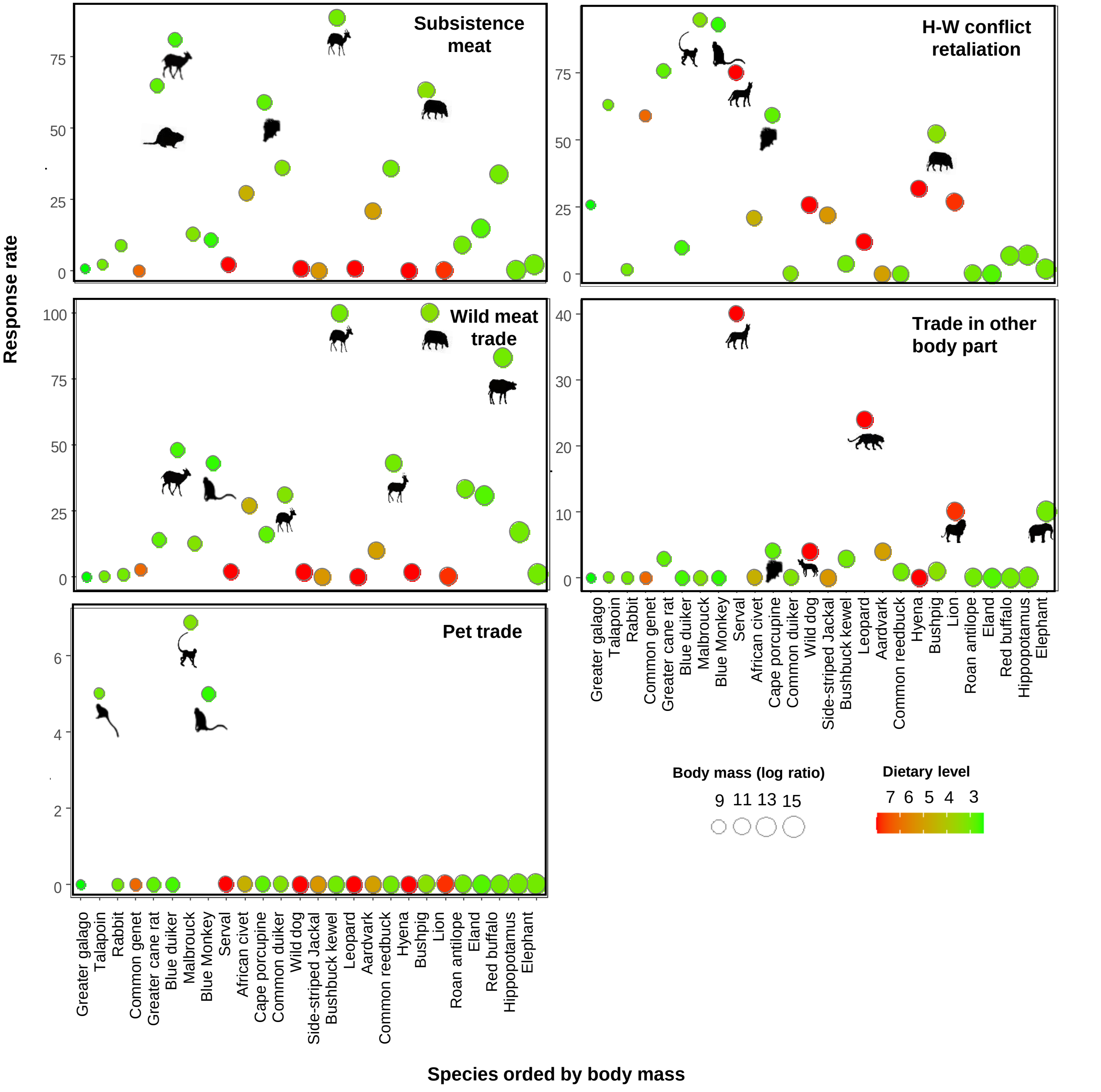

Figure 5

Click here to access/download;Figure;Figure 5.pdf

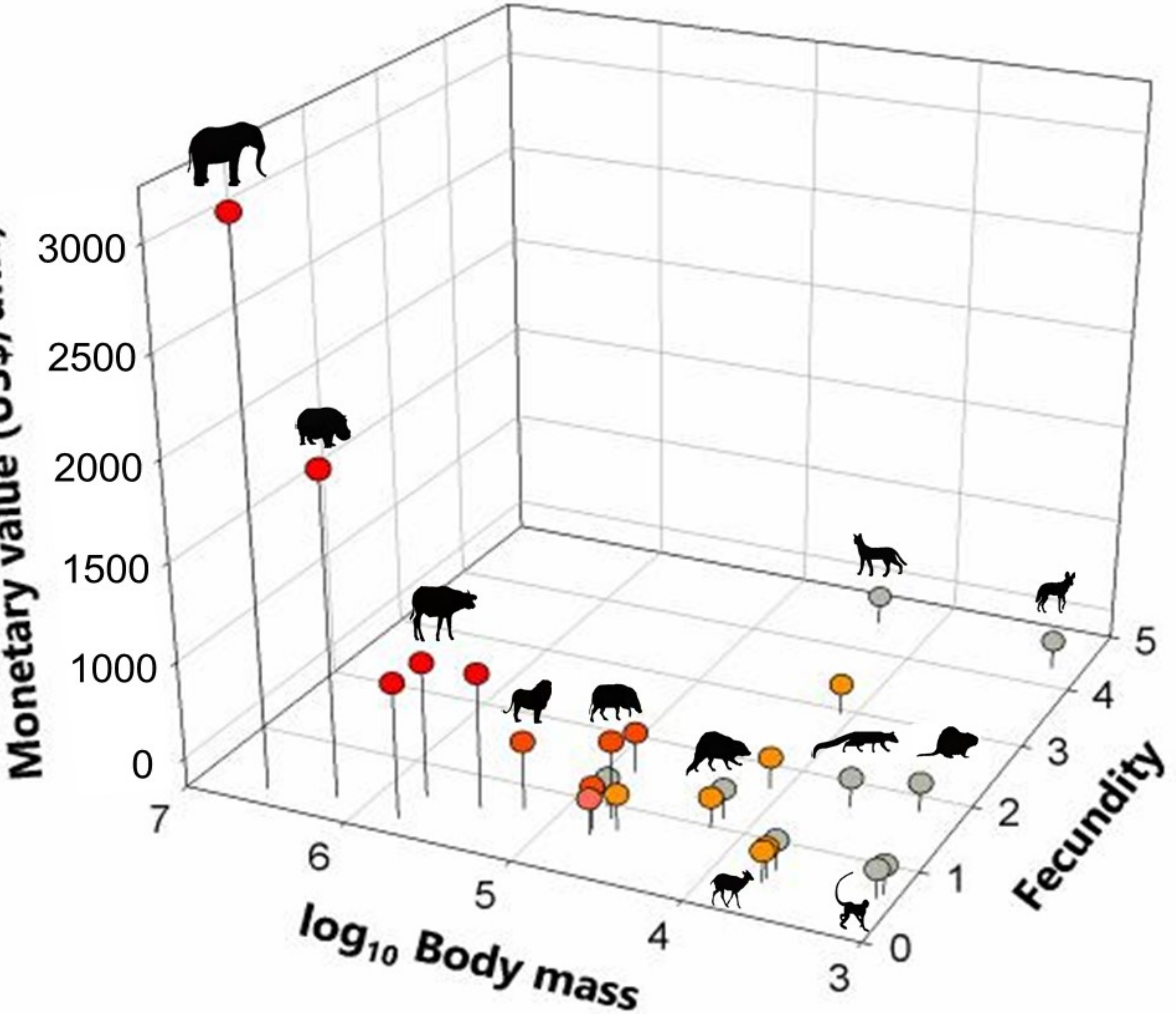

Click here to access/download
Supporting Information
Supplementary material.docx

Click here to access/download
Other
Braga-Pereira et.al., 2020.pdf

related manuscript

Click here to access/download
Other
braga-pereira2020b.pdf

related manuscript

Click here to access/download
Other
116-E-book-340-1-10-20181025.pdf

Intrinsic and extrinsic motivations governing prey choice by hunters in a post-war African forest-savannah macromosaic

Formatted: Space After: 0 pt

Franciany Braga-Pereira [1,2,3*]; Carlos A. Peres [4,5]; Rômulo Romeu Nóbrega Alves [1,6]; Carmén Van-Dúnem Santos [3]

1- Department of Ecology and Systematics, Universidade Federal da Paraíba, João Pessoa, PB, Brazil

2- Rede de Pesquisa para Estudos sobre Diversidade, Conservação e Uso da Fauna na Amazônia (RedeFauna), Manaus, Amazonas – Brasil

3- Agostinho Neto University, FCUAN, Avenida 4 de Fevereiro, 71, Luanda, Angola

4-Instituto Juruá, Rua das Papoulas, 97, Aleixo, Manaus, AM, 69083-300, Brazil.

5- School of Environmental Sciences, University of East Anglia, Norwich, UK

6- Department of Biology, Universidade Estadual da Paraíba, Campina Grande, 58429-500, Brazil

*Corresponding author: franbraga83@yahoo.com.br

SUMMARY

Overhunting typically increases during and after armed conflicts, and may lead to regional-scale defaunation. The mitigation of hunting impacts is complex because, among other reasons, several intrinsic and extrinsic motivations underpin the elevated deployment of hunting practices~~ and vertebrate populations range widely in their resilience to hunting.~~. Here we present the first study focusing on these ~~issues~~motivations in a post-war zone. ~~We found that, following~~Following persistently heavy hunting pressure during the 27-year Angolan civil war~~, the offtake of small to medium-bodied species has increased recently as a result of large mammal depletion. However~~, prey choice associated with different motivations varied in terms of species trophic level and body ~~mass~~size. While most residents hunted large-bodied species to maximize revenues from ~~the~~ wildlife trade, many low-trophic level smaller species ~~at low trophic levels~~ were harvested to ~~supply~~meet local subsistence demands because they were more palatable and could be captured using ~~rudimentary~~artisanal traps near hunters' households. Small-bodied species across all trophic levels were also ~~killed because they were~~ frequently ~~involved~~killed in ~~human-wildlife conflicts~~retaliation for crop-raiding or livestock depredation. Considering all game species sampled in this study, 96% were captured to attend two or more motivations. In addition, hunting associated with different motivations was partitioned in terms of age and gender, with prey acquisition for the wildlife trade primarily carried out by adult men, while hunting to meet local subsistence needs and inhibit ~~crop-raiding or livestock depredation~~human-wildlife conflicts were~~ in addition to adult men~~ carried out by adult men and women,

Formatted: Pattern: Clear (White)

children and even the elderly~~ frequently killed animals.~~. In natural savannah areas lacking fish as a source of protein, a higher number of species was selected to supply both the meat trade and subsistence, while more species in forest areas were targeted for trade in animal body parts and conflict retaliation. Finally, ~~trade~~local commerce in bushmeat and other body parts accrued higher domestic revenues compared to any alternative sources of direct and indirect income. However, these financial benefits were at best modest, largely unsustainable in terms of prey population collapses, and generated high long-term costs for the local to regional scale economy~~, the renewable natural resource capital~~ and native biodiversity.

Key- words: Angola, ethnozoology, local knowledge, mammals, illegal hunting, wildlife trade

1 INTRODUCTION

Hunting of wild terrestrial vertebrates, as one of the oldest activities pursued by ~~mankind~~both archaic and modern humans, has been undoubtedly critical in human ecology and evolution because hunting represents a form of defence against wild animals and procurement of food, clothing and therapeutic products (1–3)~~(1–3).~~. However, hunting practices have resulted in large-scale defaunation, and even subsistence hunting to meet local needs represents one of the primary drivers of local extinctions of wildlife populations (4–6). In many tropical regions, hunting ~~has~~exerts become a more severe threat to native wildlife than primary habitat loss via the conversion of natural vegetation (7,8). Moreover, the potential ecological effects of

overhunting are not only on populations of game species, but also on the overall structure and dynamics of natural ecosystems (5,9), and overhunting places local livelihoods and food security at risk (10). Large-bodied mammals are often the most frequently selected prey species for the bushmeat trade due to their larger meat yields and greater returns on energy invested in hunting (11,12), in addition to being more ~~easily detected by game hunters in open landscapes ().~~intrinsically detectable for game hunters in open landscapes (13,14). However, these mammal species generally exhibit lower fecundity rates, larger home ranges and lower population densities, which result in higher vulnerability to population depletion and local extirpation (15–17). Species-specific hunter selectivity is often also both size- the sex-biased, so that the impact of hunting tends to be higher on the survival of sexually mature males, particularly in size-dimorphic polygynous species (18)~~(18)~~. However, selective hunter-induced mortality can also be markedly sex-biased in weakly dimorphic species depending on the hunter preferences and hunting regulations, if any (19,20).

Overhunting has increased in the last three decades partly due to widespread road expansion, more affordable transportation and hunting technology, population growth, higher market demand, and the use of more efficient hunting methods (20,21,22). In addition, all of these aggravating factors tend to be correlated with each other. For example, the contemporary use of shotguns in some traditional communities in Cameroon is related to the emergence of more specialized and highly efficient hunters with clear market orientation (24). A critical element of effective conservation is

Formatted: English (United Kingdom)

Field Code Changed

therefore a proper understanding of the range of motivations propelling hunting behaviour in humans (25).

A comprehensive list of typologies of illegal hunting motivations could include household consumption, commercial gain, recreational and trophy killings, protection of self and property, poaching as a traditional right, gamesmanship, disagreements with specific regulations and subversive poaching (26). Moreover, we can essentially distinguish hunting motivations into either intrinsic, when the demand for game kills arises from *in situ* concerns in which the hunters themselves are embedded, or extrinsic, which arises from a motivation outside the community and often accounts for the largest fraction of the animal biomass offtake (27,28). For example, the perceived risk of livestock predation by wolves in Spain and jaguars in Brazil are key motivations for the systematic poisoning of these animals (29). In addition, wild meat (including terrestrial and aquatic vertebrates) represents the main source of protein in the daily diet of traditional populations worldwide (30), and is critical for the well-being and nutrition of local communities (31). Hunting intended for self-consumption also includes animals selected for therapeutic purposes (32), amounting to intrinsic motivations to hunt. On the other hand, commercial offtake of elephants and rhinos is primarily motivated by the exceptional market value of ivory and horns, respectively. Moreover, the illegal trade in bushmeat and other body-parts has led to many social and economic problems as local prices of commercially valuable species and their derivatives continue to rise, which in turn generates ever greater incentives for this illegal practice (33,34).

Individual-based motivations can become even more complex at sites experiencing episodes of socio-political upheaval, which can increase demand in meat and other body parts and effectively lift restrictions on game hunting. For example, during civil wars in low-governance countries (outside of land mine zones) hunters become more encouraged to hunt due to sociopolitical changes induced by the uprise of conflicts (e.g. suspension of anti-poaching patrols, increased access to automatic weapons, and growing demand in urban markets). Increased market demand can be fueled by diverse sets of consumers, including rural war refugees, urban elites, and the international gourmet meat market. In addition, high-value animal by-products from the illegal wildlife trade (such as elephant ivory and rhino horns) are often exchanged to finance militias. During the post-war period, however, large mammal populations will continue to decline unless there is a concerted cooperative effort to protect natural ecosystems and their wildlife (14,35). Regulation of high-value trade in threatened species also requires coordinated efforts to manage procurement. Few studies have focused on these intrinsic or extrinsic motivations underlying hunting and/or the mechanistic motivations underpinning game pursuit and choice, and ultimately why some mammal populations are more impacted than others.

Here, we examine the patterns of hunter motivation in a post-war zone in relation to species body mass and trophic level, including capture rates of individuals in different sex and age classes. Considering that conservation problems are inextricably linked to socioeconomic context, we also examine the degree to which the illegal wildlife trade has been more profitable in the short term than alternative livelihood

activities pursued by local residents. To do so, we conducted interviews using a robust sample size of local hunters within the two most important protected areas of Angola, Southwest Africa. Finally, we show that different ~~motivations~~forms of motivation bias hunting selectivity in terms of species body mass, trophic level, and availability and further discuss the economic ~~motivations~~drivers behind hunting practices.

2 MATERIAL AND METHODS

2.1 Study Landscape

This study was carried out at Quiçama National Park and Quiçama Game Reserve, which span a total area of 960,000 ha (9°09'–10°23'S and 13°09'–14°08'E) and represent the most important conservation areas of Angola (36). We selected eight human settlements distributed throughout this area (Fig. 1). A government census showed that there were 25,086 residents in the Quiçama municipal county in 2014. Although this local population is not legally permitted to occupy these two protected areas, they have done so for generations, which creates tensions between residents and the reserve management. The semi-subsistence economy of the households in the area is sustained by hunting, small-scale fisheries, slash-and-burn cassava agriculture, and non-timber forest products. In early 2000, a rehabilitation project (Operation Noah's Ark) conducted a species ~~relocation~~translocation program to the Special Conservation Area, but unfortunately many of these species were exotic to the park (37). These introduced and reintroduced alien species are not considered in this study.

Figure 1. Location of Quiçama National Park (demarcated by the greenblue line) and Quiçama Game Reserve (orangered line) in Southwest Africa. Solid yellow circlestriangles indicate the surveyed townships, which encompassed more than one human settlement. CircleTriangles sizes are proportional to the population size of the townships. Green and brownish background areas represent forest and savannah environments, respectively. Map generated using ArcGIS 10.3.1; Datum: WGS84. Elaborated by Ana Caroline Imbelloni; Franciany Braga-Pereira in August/2021.

Formatted: Font color: Text 1

Formatted: English (United Kingdom)

2.2 Ethics approval

This research was approved by the i) National Health Council (Resolution 466/12), through the Research Ethics Committee of the Universidade Federal da Paraíba (license number 59846816.3.0000.5188); ii) Environmental Ministry of Angola (148INBAC.MINAMB/2016); iii) municipal administration of Quiçama (license number 017/GAB.ADM.MQ/2017); iv) leaderships of all sampled villages. An informed consent form was given to the interviewee, stating the purpose of the interview and ensuring the interviewee's anonymity, so each interview could not begin until after this form had been signed.

Formatted: Pattern: Clear

2.3 Data collection

We used Local Ecological Knowledge (LEK) to obtain information on hunting and wildlife use. The first contact between FBP and community dwellers occurred in 2014 to delineate the study area and present proposals to local villagers prior to deployment of this study. This facilitated access to the study area and a relationship of trust with local informants, including dedicated and semi-professional hunters. Data for this study were obtained from January 2014 to January 2015 and from January to April 2017. Translators were not required because both the interviewer and local informants spoke Portuguese. Interviews were conducted with 115 experienced local hunters (112 men and 3 women), who ranged in age from 19 to 80 years old. These hunters were selected using a snowball sampling technique (38), and on average we interviewed 14 informants per settlement. In addition to engaging in hunting activities, interviewees also occasionally worked as small-scale farmers, fisherfolk, teachers and community leaders.

Field data were collected through semi-structured individual interviews (39) with the help of a visual stimulus checklist using colour photos of mammals living within the research area. For each game species reported as killed by the interviewed hunter, he/ she was asked to provide information on the (i) the date of the most recent mammal specimen killing; (ii) sex and age class of the most commonly hunted individuals; (iii) motivation behind the pursuit each wild mammals ; and (iv) the revenue acquisition from the sale of the animal when traded including the trade value (per kg) as: a) live whole animal, b) whole dead animal (e.g. fresh meat), c) processed as dried meat, d) processed skin, and e) ivory. We were therefore able to quantify the total aggregate

value of each prey item. Each hunter was also asked about sources and amounts of income acquired from wage labour and other activities he or she may have performed, including farming, teaching and community leadership~~.~~ (see the complete questionnaire in Supplementary Material).

2.4 Data compilation

As all questionnaire answers were open-ended, in order to facilitate data analysis different answers to the question “what is the motivation behind the pursuit [this] species?” were classified into common themes following Braun and Clarke (40). Coded responses were summarized, thereby resulting in five hunting motivation categories: meat trade, pet trade, other trade in body parts, subsistence needs, and conflict retaliation. This method of open-ended questioning provided the flexibility to explore, whenever necessary, different lateral topics of relevance while providing rapid anthropological assessments valid for wildlife research. Finally, we organized a binary data frame structured as species and interviewees in rows and hunting motivations in columns, in which the absence or presence of a specific motivation associated with a specific species, according to each interviewee, was attributed the values 0 or 1, respectively.

Formatted: Font color: Auto

We used the PanTHERIA database (41)~~(Jones et al 2009) to obtain information on body mass and trophic level of each species. Based on the~~ to obtain information on body mass and trophic level of each species. Based on specific morpho-ecological traits, all mammal species were assigned to seven dietary functional groups. We initially ranked

the energetic stratum of the modal dietary pattern of each species as follows: (1) ~~grazer/~~folivore < (2) frugivore < (3) granivore < (4) insectivore < (5) myrmecophage < (6) mesocarnivore < (7) hypercarnivore. We then weighted the proportion of each major dietary mode of any given species (sourced from (42,43) ~~(Wilman et al 2014; Bogoni et al 2019) given these food energy levels (e.g. if the grazer/frugivore *Tragelaphus oryx* consumes 70% grass and 30% fruit, its mean trophic energy level would be 2.7 (= (0.7 × 3) + (0.3 × 2)).~~given these food energy levels (e.g. if the grazer/frugivore *Tragelaphus oryx* consumes 70% foliage and 30% fruit, its mean trophic energy level would be 1.3 (= (0.7 × 1) + (0.3 × 2)). We used the PanTHERIA and AnAge databases (44)~~(Tacutu et al 2018)~~ to obtain information on species litter size and number of litters per year. Per capita annual fecundity (defined as female young per adult female per year) were therefore calculated as (litter size × number of litters per year)/2 (i.e. assuming a 50:50 birth sex ratio)(45). The monetary value of each animal is expressed as the mean transaction value per individual reported for each species by all independent interviewees. For details of English and scientific species name, and species body mass annual fecundity rate, trophic level and monetary value acquired per animal see Supplementary material, Table 1.

We assigned the 8 sampled communities into four study sites according to type of landscape structure and geographic position. We therefore sampled two communities in savannah areas of northern Quiçama; two in savannah areas of southern Quiçama; two in central forest areas; and two communities in southern forest areas. Some differences between these sites include: the northern savannah communities are near (~20 km) a

semi-urbanized area, and influenced by a nearby paved road. The central/southern forest sites had access to the highest large mammal abundance in Quiçama and access roads were in poor condition. The southern forest sites are near lakes and small streams, so the local population could also meet their protein needs from fish. The southern savannah site, although far from an urban center, had access to a road in good condition, thereby facilitation trade. There were some lakes and small rivers at this site, which could also supply some animal protein from fish.

2.4 Data analysis

~~We performed generalized linear models (GLMs) to~~*Species traits associated to each hunting motivation*

To examine the effects of body mass and trophic level on the number of hunters reporting each hunting motivation for killing any given species~~.~~ we performed generalized linear mixed models (GLMMs). We performed five different models associated with each hunting motivation. We considered i) the hunting motivation as a response variable; ii) species body mass and trophic level as fixed effects predictor variables; and iii) interviewee as a random factor. Given that this is a ~~continuous variable (including zero~~binary response (0 or 1), we used ~~a negative~~ binomial distribution. There was no observed collinearity (p >0.05) among predictor variables. ~~Each model was compared~~We used residual checks to ~~a null model using AIC values, and~~ verify whether our models were ~~selected considering the~~ , in principle, suitable or

Formatted: English (United Kingdom)

otherwise. We used the Akaike information criterion to select models of interest if ΔAIC values >6 (ΔAIC obtain from the difference between a null and complete model AIC values~~ of ΔAIC>6)~~ (46).

To test whether different hunting motivations, and species body size and trophic level were related to each other, we performed multiple factor analysis (MFA) (47). Compared to the frequently used principal component analysis (PCA), MFA takes into account the fact that data are structured into groups (here different species) to balance the importance of each group in the analysis. Species traits then become more important when any given species is repeatedly cited by several interviewees.

Local revenues from wildlife offtake

We performed linear models (LM) to examine the strength and direction of linear relationships between prey body ~~mass~~size and fecundity rate; body ~~mass~~size and local revenues accrued from sales of each species; and fecundity rate and local revenues. ~~Once in each~~ Given that only one ~~of these LM there is just one predict~~predictor variable ~~of fixed effect~~was explored in each LM, we used frequentist statistics to ~~evaluate the effect of the~~ assess variable effects, including F-values, degrees of freedom, p-values and adjusted R^2-values.

Hunter demography associated with hunting motivations

To examine the effects of age, gender and natal community on the number of hunters reporting killing motivations for different species, we performed generalized linear

Formatted: Font color: Auto

models (GLMs). We structured five different models associated with each hunting motivation. We considered i) hunting motivation as a response variable; and ii) age, gender, and natal community of each interviewee as predictor variables, in the case with p-value.and used a binomial distribution to model the binary responses. We used residual checks to verify whether our models were, in principle, suitable or otherwise. We used the Akaike information criterion to select models of interest if ΔAIC values >6 (ΔAIC obtain from the difference between a null and complete model AIC values) (46). These GLMs ensured we could assess which catchment area were associated with a higher number species hunted by a larger number of hunters according to each hunting motivation. MFAs were further performed to examine the degree to which hunting motivations were the same across all game animal age and sex classes, and hunting sites.

All analyses were performed in R ver. 3.5.3 (R Core Team 2019). GLMMs and GLMs were based on the *lme4* (48) package and MFAs were based on the *FactoMineR* (47) package.

3 RESULTS

Formatted: English (United Kingdom)

3.1 Prevalence and history of kills and game choice

The speciesSpecies reported as captured by all interviewed hunters were bushbuck kewelBushbuck Kewel and bushpigBushpig. Blue Monkey and Blue Duiker were also killed by 94.7% and 93.0% of all interviewed hunters, respectively. Species killed by a smallersmall number of hunters included hippopotamusHippopotamus (16.5%) and

~~elephant~~Elephant (10.4%). Most of the recent capture records for small and medium-sized species had occurred within the previous 10 years, with few exceptions such as ~~serval, wild~~Serval, Wild dog and ~~hyena~~Hyena. These former species followed the pattern of large mammals, for which the most recent captures occurred within 20 to 50 years prior to interviews (Fig. 2). Adult animals were far more frequently selected, as reported by 89.6% of all informants, with the remainder indicating that animals were either selected regardless of age class (8.0%) or that young animals were more frequently selected (2.4%). In relation to sex classes, most informants (54.9%) reported that there were no differences in the frequency with which either males or females were selected, although 39.5% indicated that males were mainly targeted, with only 5.6% of all informants indicating that females were more frequently selected.

Figure 2. Records of the most recent year in which each interviewee had killed a mammal species. Data are presented in 10-year increments for historical (1967-2016) and modern periods (2017). Response rates were calculate from the number of hunters reporting killing each species. Species are denoted by their English names and ordered top to bottom from the smallest to the largest species.

3.2 Motivations driving prey acquisition

From those hunters reporting intentionally capturing each species, the main intrinsic motivations were subsistence meat acquisition and retaliation against wildlife posing perceived threats; while extrinsic motivations were ~~the~~ bushmeat~~, bodyparts and pet~~

trade~~.~~, and trade in animal body-parts and pets. With the exception of Greater Galago, all other game species were captured to attend two or more motivations and seven species to attend for out of the five motivations (Common Duiker, Bushpig, Blue Monkey, Cape Porcupine, Malbrouck, Greater Cane Rat and Talapoin) (Supplementary Material, Fig 1). Some hunters also used some animal body parts for zootherapeutical ~~species body parts~~purposes, but this was rarely the primary motivation for killing ~~a~~any given species. All respondents reported killing at least one species for food, retaliation and ~~sale~~sales of meat. However, trade in body parts was restricted to only 40% of all informants.

Formatted: Default Paragraph Font, Font: +Body (Calibri), 11 pt, Portuguese (Brazil), Pattern: Clear (White)

Species hunted to supply both subsistence needs and the meat trade were trophically similar and primarily herbivores at low dietary energy levels. . The first dimension (Dim$_1$) of the MFA accounted for 25.9% of the variability in hunting motivation across different species (Fig. 3a). The meat trade, subsistence consumption and dietary level contributed 34%, 32% and 25% of the variance explained by Dim$_1$, respectively, whereas procurement for other body parts, the pet trade, retaliation against "problem" species and prey body size combined contributed with only 10% (Fig. 3b). The second MFA dimension (Dim$_2$) explained 19.6% of the variance with retaliation and body size explaining most of the variance (37% and 32%, respectively), whereas other factors were relatively unimportant (pet trade 14%, trade in body-parts 10%, subsistence needs 5%, dietary level 2% and the meat trade 2%; Fig. 3c).

Figure 3. Species compositional similarity in terms of hunting motivations obtained from (a) a multiple factor analysis (MFA). Contributions of each hunting motivation and species trait for (b) the first (Dim_1) and (c) the second dimension (Dim_2). The red dotted line indicates the percentage that would be obtained if all factors contributed equally to the overall variance.

Local meat consumption was associated with the killing of species at low trophic levels ($p < 0.001$), yet harvested prey spanned the entire spectrum of body mass, from ~~cane rats~~Cane Rats (4 kg) to ~~bushpigs~~Bushpigs (69 kg). Killings justified as retaliation ~~for~~against human-wildlife conflicts involved mainly smaller-bodied species ($p<0.01$) across all trophic levels, for reasons including crop-raiding (e.g. primates such as ~~blue monkeys~~Blue Monkeys and ~~malbrouck monkeys~~Malbrouck Monkeys) and predation on domestic livestock, fish or wild animals of human interest (e.g. ~~felids feeding~~felid depredation on chickens). For the bushmeat trade, species at low trophic levels were cited as significantly positively selected ($p < 0.001$), particularly large-bodied species. Kills to fuel the trade in ~~wild~~ animal ~~by-products~~body parts included mainly felids ($p < 0.01$) of varying sizes and intentional captures of ~~monkey~~primates were also motivated by the pet trade (Fig. ~~3,~~4; Table 1). Animal products were often sold to intermediaries who, in turn, traded at higher profits outside local communities. Although informants were unsure about reporting the ultimate motivation behind felid skeletal parts traded, they believed those would be used largely for ornamental or medicinal purposes either within Angola or other countries. In addition to Carnivores, other prey species provided

Formatted: Indent: First line: 0.5"

body parts that were secondarily used for medicinal purposes by local communities. However, ~~honey badgers~~Honey Badgers (*Mellivora capensis*) were reportedly killed primarily for ~~medicinal~~therapeutic purposes (Table 2).

Formatted: Heading 21, Font: 11 pt, Portuguese (Brazil)

Figure ~~3~~4. Species selected to meet different types of hunting motivations. Response rates were calculate from all interviewed hunters (n=115). Species are denoted by their English names and ordered left to right from smallest to largest~~ species~~. Circle sizes are proportional to species body mass; circle colours denote trophic level in terms of dietary energy, ranging from green (lowest) to red (highest).

Table 1. Details of the complete ~~model~~ and ~~the~~ null model using generalized linear ~~model for verify~~mixed models (GLMMs) to examine the effects of body ~~mass~~size and ~~trophic~~dietary level on the number of ~~hunter~~hunters reporting each form of hunting motivation for killing ~~a~~any given species. Estimated values indicate the ~~coefficient~~coefficients associated with the variable listed on the left. This represents the estimated amount by which the odds (~~of~~that each response variable would increase if each explanatory variable were one unit higher). Z-values indicate the degree to which explanatory variables exert a significant effect. Pr (>|z) denote significance levels as following: ns P > 0.05; ** P ≤ 0.01; *** P ≤ 0.001.

Response variable	Predictors	Estimate	Std. Error	z value	Pr(>|z|)		AIC	AIC Null model	ΔAIC
Subsistence meat	Body mass	-1.74E-06	6.96E-07	-2.502	0.0124	*	2604.5	3117.1	512.6
	Dietary level	-7.63E-01	5.18E-02	-14.755	<2e-16	***			
Wild meat trade	Body mass	-4.49E-04	6.97E-04	-0.644	0.519		2846	3260.7	414.7
	Dietary level	-7.29E-01	4.93E-02	-14.79	<2e-16	***			
Trade in other body parts	Body mass	2.94E-04	7.03E-04	0	0.675		1006.6	1113.9	107.3
	Dietary level	4.11E-02	5.11E+01	10.590	<2e-16	***			
Pet trade	Body mass	-8.90E-02	1.40E-03	-63.776	<2e-16	***	376	479	103
	Dietary level	-1.257	4.18E-01	-3.000	0.0027	**			
Retaliation	Body mass	-1.74E-06	6.96E-07	-2.502	0.0124	*	2604.5	3602.4	997.9
	Dietary level	-7.63E-01	5.18E-02	-14.755	<2e-16	***			

Formatted: Right: 0", Line spacing: 1.5 lines
Formatted: Right: 0", Line spacing: 1.5 lines
Formatted: Left, Right: 0", Line spacing: 1.5 lines
Formatted: Right: 0", Line spacing: 1.5 lines
Formatted: Right: 0", Line spacing: 1.5 lines
Formatted: Right: 0", Line spacing: 1.5 lines
Formatted: Right, Right: 0", Line spacing: 1.5 lines
Formatted: Right: 0", Line spacing: 1.5 lines
Formatted: Right: 0", Line spacing: 1.5 lines
Formatted: Right: 0", Line spacing: 1.5 lines
Formatted: Right, Right: 0", Line spacing: 1.5 lines
Formatted: Right: 0", Line spacing: 1.5 lines
Formatted: Right: 0", Line spacing: 1.5 lines
Formatted: Right: 0", Line spacing: 1.5 lines
Formatted: Left, Right: 0", Line spacing: 1.5 lines
Formatted: Right: 0", Line spacing: 1.5 lines
Formatted: Right: 0", Line spacing: 1.5 lines
Formatted: Right: 0", Line spacing: 1.5 lines
Formatted: Left, Right: 0", Line spacing: 1.5 lines
Formatted: Right: 0", Line spacing: 1.5 lines

Table 2- Species withMammal species and their respective body partparts used for medicinal and "magical" purposes inwithin Quiçama National Park and Quiçama Game Reserve, Angola, Southwest Africa

Species/Vernacular name	Ailment treated	Animal body part used	IUCN status
Panthera leo - Lion	Asthma	Fat	VU
Panthera pardus - Leopard	Ward off envy	Skin	VU
Mellivora capensis - Honey badger	Physical weakness	Bone	LC
Tragelaphus scriptus - Bushbuck kewel	Personal animosity	Skin	LC

3.3 Local revenues from wildlife offtake vs other activities

Large-bodied species yielded the highest household revenues obtained from sales of single prey items ($F_{1,17}$ = 299.2; p< 0.001; R^2_{adj} = 0.94). To make matters worse, speciesthese large mammals often exhibit low fecundity rate was negatively correlated with both market prices per species (p = 0.0367) and body mass (p= 0.0328)rates (Fig. 4, Supplementary material, Table 25). The five most valuable prey species fetched an average market price per individual carcass of US$ 1359 (range = US$ 638 - 3117 per individual). These species, however, were becoming scarce and had not been recently captured by local hunters (Fig. 2).

When comparing with theCompared to revenues provided by medium and large-bodied species, local revenues accrued from any alternative wage labour option were very modest and well below the poverty line for Angola, withwhere monthly income values ofare US$98, US$43, and US$33 for teaching, farmingteachers, farmers, and community leadership roles, respectively. In comparison, the value accrued from a

Formatted: Line spacing: 1.5 lines

Formatted: Line spacing: 1.5 lines

Formatted: Not Superscript/ Subscript

Formatted: Article Title, Font: +Body (Calibri), 11 pt, Portuguese (Brazil)

single kill exceeding 50 kg was considerably greater. For example, annual revenues from gazelle kills (*T. scriptus)* alone were twice that from teaching, and the sale value of a single leopard skin was nearly four-fold higher than the monthly wages of a primary school teacher, who earned a higher income than either a farmer or ~~a~~ community leader.

Figure ~~4. Value acquired for~~ 5. Monetary values obtained from each ~~specimen~~prey item traded~~. Species~~ in the local market. Mammal species are ordered ~~in the axe~~along different axes i) z: from top to bottom from least to most profitable~~ species~~; ii) x: from left to right from largest to smallest~~ species~~; and iii) y: from left to right from lowest to highest fecundity rate. Warmer colours indicate higher offtakes in monetary values. Dollar values were based on the 2017 Kwanza conversion rate (US$1 = 165 Kwanzas).

Formatted: Add space between paragraphs of the same style

Formatted: Default Paragraph Font, Font: +Body (Calibri), 11 pt, Not Italic, English (United Kingdom), Pattern: Clear (White)

Hunter traits and hunting motivations

In relation to the sites (regarding landscape structure and geographic position), GLMs showed that a higher number of kill reports in northern savannah areas were due to subsistence and the meat trade than in either southern savannah and forest sites. Forest sites showed a higher number of prey offtake reports induced by retaliation killings and trade in other body parts compared to savannah sites (Supplementary Material, Table 2). This is consistent with the MFA analysis which showed a high overlap of species selected for the meat trade and subsistence in northern savannahs, and a similar set of species killed due to the body-part trade or conflict retaliation across all

forest sites (Supplementary Material, Fig 2). Although middle-aged male hunters more frequently reported mammal kills, sex and age classes of informants had a low contribution in explaining MFA dimensions 1 and 2 (Supplementary Material, Fig 3). Hunting for the wildlife trade was primarily done by adult men, whereas kills attributed to local crop-raiding or livestock depredation and local subsistence, were also carried out by adult women, children and the elderly.

4 DISCUSSION

In the early stage of the Angolan civil war, target mammal species ~~were comprised~~ harvested at the Quiçama National Park region of Angola consisted primarily of medium- to large-bodied species~~ in this region.~~. This was consistent with the set of game species sold in the markets in Luanda (capital of Angola), which were commercially valuable as either bushmeat or other body parts. However, as large-bodied mammals became increasingly overhunted and their populations ~~increasingly~~commensurately depleted, the size structure of prey species gradually shifted towards small to medium bodied species (14). This explains why recent mammal captures are largely comprised of small to medium-sized species. In addition, even if the most important motivation associated with kills of large-bodied species was the wildmeat trade, medium and small-bodied species were also captured to supply the bushmeat market. These species were also often captured for other reasons, such as subsistence needs and retaliation against wildlife posing threats.

Given the temptation to participate in the bushmeat trade, some informants explained that they were prepared to invest several days searching more remote areas of Quiçama in pursuit of large-bodied mammals even if their numbers had been depleted. This is consistent with a study in Indonesia, where market demand and supply of wild pigs was unaffected when local wild pig populations collapsed because hunters simply travelled farther in pursuit of game (49). This hunter-prey system must, however, be carefully evaluated because despite the great effort by some hunters to capture species providing high energy and monetary returns, several species are absent from prey profiles in the recent years, which likely indicates severe population declines if not local extinctions. Among the five largest and most profitable species, red buffalos were recently slaughtered, but their remaining populations are now restricted to remote forest areas in the southern Quiçama region. Those buffalo populations, therefore, should receive immediate conservation attention, to prevent regional scale extirpation such as the case of native populations of Eland and Roan Antelope.

Intensification of hunting activities over the last decade can be understood given the dire post-war economic crisis in Angola, which is now further exacerbated by the COVID-19 outbreak that is plaguing the country. As a consequence, hunters from Northern Quiçama are hunting in the proximity or within the special protection zone of QNP, which corresponds to 10% of the park area that is patrolled on a daily basis by 15 rangers. This area and even the most remote portions of Quiçama have become increasingly accessible to not only local residents, but also outsiders in search of desirable game and other nontimber products. In a plight similar to that of red buffalos,

native elephant populations at Quiçama are now restricted to forest landscapes where they are less detectable (14). Elephant populations in Angola as a whole are experiencing intense poaching pressure as well as losing habitat to expanding human settlements. Based on observed carcass ratios, a study has shown that elephant populations in southern Angola started to increase after the war until 2005, but then began to decrease at an annual rate >2% (50)(Schlossberg et al 2019). Although many landmines remain in southeast Angola following the civil war, and elephants have been killed by mines (51) poaching for ivory trade, rather than landmines, is the primary cause of mortality based on a large number of carcasses observed in a survey conducted in 2015 in southern Angola(50). As a consequence, Angola is in the midst of one of the worst poaching crises of any country within the savannah range of African elephants and nationwide population declines will almost certainly continue if the drivers of mortality are not reversed.

Hunters reported that they often captured more than one individual larger than 40 kg each month to maximize income from bushmeat sales, but that those species often failed to contribute meat to their household subsistence for several months. In this case, only less desirable leftovers from dressed carcasses of large animals, such as the head and hooves, would be locally consumed, with consumption of whole prey items of large mammals in the hunter's household restricted to the most important holidays. This partially explains why body mass did not exert a significant effect on species selectivity in terms of community-level subsistence, given that more frequent local consumption of smaller-bodied species can be explained by both the

Formatted: Font: 12 pt

Formatted: Font: 12 pt

Formatted: Font: 12 pt

Formatted: Font: 12 pt, Font color: Auto

Formatted: Font: 12 pt, Font color: Auto

Formatted: Font: 12 pt

Formatted: Font: 12 pt, English (United States)

Formatted: Indent: First line: 0.5"

higher abundance of those species and the fact that larger species were primarily directed to the bushmeat trade. However, another important factor to be considered is overall preference in terms of palatability. For example, interviewees reported that medium-sized mammals such as Blue Duiker (5 kg) have "soft tasting" meat compared to several larger species such as Red Buffalo (600 kg). Also, villagers typically complied with some important dietary taboos regarding primates, which were rarely consumed at Quiçama even though they were one of the most abundant mammal taxa larger than 1 kg.

Primates at Quiçama were killed primarily in retaliation against crop raiding and livestock depredation and to supply the pet trade, with exception of Blue Monkeys (6 kg), reported by many hunters as killed to fuel the bushmeat trade. Although primates were rarely used as a source of meat across the study area, this practice is common in other African and Neotropical sites (52), and they account for the largest number of mammal species (126) threatened by hunting worldwide (5). Given plausible scenarios of wildlife depletion, however, hunters in the future may become less selective and gradually change their hunting practices to include primates as game species, which could be directed to either the bushmeat trade or household subsistence (14). Local use of mammals for medicinal purposes did not necessarily involve new kills since the animal parts used were essentially minor byproducts of prey acquisition to meet other purposes. In addition, there are cases in which the medicinal exploitation of wild mammals involved the non-invasive use of scats or dung, rather than actual mortality (32).

In Quiçama, the median abundance value of all large and medium-sized game species, and some small-bodied game species (Blue Duiker and Blue Monkey), in savannah areas declined during the war and did not recover during the post-war period. In contrast, in forest landscapes, 15.8% of all large and medium-sized game species, whose abundance had declined during the war, have since experienced post-war population recovery (14). This helps explain why hunting for both for subsistence and meat trade encompassed a larger number of species in the northern savannahs than in the forest sites; since savannah areas are the most defaunated, prey acquisition in this environment follows a generalist hunting strategy that goes well beyond selection of preferred species. On the other hand, hunters in the southern savannah areas had access to alternative fish protein, which was either consumed locally or sold. Hunters at these sites would thus also go fishing but concentrate on the most desirable game species when hunting. In terms of non-food motivations, the number of reports of species killed for trade in other animal body parts and retaliation was higher in forest than in savannah areas, because forest sites hosted the highest abundance of species whose body parts are traded (such as leopard skin and elephant ivory); and crops and domestic livestock were nearer a wider spectrum of crop-raiders and larger carnivores. In recent years, residents of forest sites in the Center-South of Quiçama have asked the local government for support because elephants that emigrated from the region during the war years, are currently returning to their habitat and raiding local crops.

We found that species at the lowest dietary trophic levels were the most frequently captured for both the wild meat trade and local subsistence. This is

consistent with data from kill profiles in Afrotropical forests showing that bushmeat harvest rates of frugivore–herbivores are the highest, both in terms of the number of carcasses (44.5%) and overall biomass (57.2%) (53). However, carnivores were frequently involved in human-wildlife conflicts and were also affected by commercial use of body parts for both medicinal (bones and fat) and ornamental (skin and teeth) purposes. Many leopards were incidentally caught in traps set for antelopes, and then subsequently killed for the illegal wildlife trade. Given that carnivore skin prices are based on quality which is reduced by bullet perforations, some hunters at Quiçama either hanged the animal or waited for it to die from bleeding, rather than immediately killing the animal. Protecting hide quality from projectile damage also explains why many carnivores were poisoned to death rather than shot(54).

Considering the hunting motivations described here (subsistence, animal trade and conflict retaliation), similar motivations were found in a study carried out in Tanzania, which showed that food acquisition and generation of income (mentioned by 79% and 78% of all interviewees, respectively) were most frequently cited as to why hunters poached(28). A review on hunting motivations in Vietnam also showed that crop protection and cash income were the main motivations behind hunting (55). On the other hand, a survey conducted in Oregon, USA, showed that recreational and trophy hunting were the main motivations driving deer hunting (56). These differences can be partly explained by the low-income populations of villagers at Quiçama who are unlikely to go hunting unless this can generate either direct or indirect income. Our results also show positive hunter selectivity targeting adults, which we attributed to

their larger size and greater profits from sales. Likewise, a global survey revealed that humans kill adult males and females, the reproductive capital of prey populations, up to 14 times more often than natural terrestrial predators, such as wolves or lions (2). As a consequence, life-history traits may often shift to early reproduction since older animals are consistently eliminated by hunters(57). We found that this also applied to males, which were generally larger and provided higher returns than females. In addition, interviewees indicated that males were more frequently captured because they are more "distracted" and consequently failed to detect active traps. Some studies have shown that such selection for adults of a single sex may lead higher reproductive investments (58,59). Additionally, due to the logic dictating that larger animals are more frequently selected in both subsistence and commercial exploitation systems (20), phenotypic changes of populations selected by humans show a 20% decline in traits related to size and 25% in traits associated with reproductive stage. These values are higher than those observed in societies in which hunters select prey only on the basis of local consumption (59).

In relation to division of labour and social norms, there was a male-bias in hunters involved in the bushmeat trade, whereas women primarily pursued smaller-bodied species for either local consumption or protecting livestock. Among modern hunter-gatherers, female exclusion from hunting large mammals is apparently closely related to the manufacture and use of traditional or fire weapons, and associated economic and/or religious norms (60). Women often have no hunting weapons of their own so their hunting activities under those restrictions are confined to small-bodied

prey. A number of anthropologists agree that the division of labour among such groups stems from culturally derived, gender-based beliefs, the importance attributed to big-game hunting, and the convertibility of these into prestige and authority that preclude female access to essential weapons, rather than fundamental sex-based anatomical or physiological differences. Male-biased hunting effort allocates to men society's most valued labour and opportunities to distribute high-value foods. Division of labour in relation to prey acquisition is thus grounded in gender politics rather than inherent sex differences (60).

Regarding the motivations across different sites, the roads that traverse savannah habitats in Northern to Southern Quiçama serves as an important route for the wildlife traffic. The greater difficulty in accessing and navigating forest areas has rendered hunting activities therein less intensive than in savannah areas (14), but we found that established traffic routes have connected cities like Muxima and Dondo to forest refugia serving as the last stronghold for buffalo and large carnivore populations. Another threat is the marked growth of urban development and extractive activities in Quiçama coastal areas. The detrimental effects of human incursion into Angolan protected areas on the abundance and distribution of large mammal species underscore the need for intensive mitigation of anthropogenic threats to restore the high wildlife conservation value in all areas impacted by the civil war (61). Also, the problem is collective, given that hunting is carried out by both residents and outsiders, often motivated by urban commerce and illegal traders of ammunition and fire weapons. Some of the arsenal typically used by hunters at Quiçama consisted of new ammunition

and automatic rifles, which indicate that they were sourced from military bases within the park, which were installed during the Angolan civil war.

Our results show that hunting is considerably more profitable in the short term compared to alternative sources of local income. It also pays to go hunting because enforcement against (and penalties related to) poaching are virtually non-existent and people cannot, and will not, protect species and natural habitats as a long-term strategy if their short-term needs are not met (28,62). However, these benefits are modest, largely unsustainable and come at a high price. They are modest because local hunters receive only the smallest share of the profits from the illegal wildlife supply chain, yet this follows a logic rationale because alternative opportunities to derive benefits from wildlife are unavailable. For example, although the income from a Cape Bushbuck sale was on average US$41 (24000kz) for a local hunter, the profits associated with selling this animal to an urban restaurant could reach US$100 (36000kz). The price discrepancy of ivory per kilogram was even greater, with local hunters earning less than US$50/kg of ivory, whereas this can fetch US$2150/kg in international markets despite the fact that ivory prices have plummeted since 2014(63). Needless to say, the ivory trade is largely unsustainable and comes at a high price in several African countries, with the value generated from the ivory trade corresponding to only 20% (US$85/km^2) of the value generated from ecotourism(64,65). A loss of up to US$41.1 million in potential annual income has therefore been estimated for some countries as consequence of bushmeat hunting. Hence, poaching should not be seen as a solution to the lack of alternative livelihoods because its food security benefits are generally

unsustainable and often brings fewer economic and livelihood benefits compared to ecotourism and trophy hunting (64,65).

The traditional population of Quiçama remains disenfranchised, in terms of healthcare, education, income opportunities and other rights. The lack of institutional capacity to effectively mitigate anthropogenic threats to both biodiversity and traditional communities at Quiçama is often justified by many as an ongoing consequence of the civil war, but there are examples that wildlife populations can recover through protection, and local residents can be ethically assisted even in the aftermath of the war. For example, after peace was declared, the Mozambique government partnered with a Foundation to restore Gorongosa National Park. Those living around parks received assistance for farming, education and healthcare and several hundreds also found jobs in the park(66). A persistent absence of institutional enforcement in countering threats to wildlife could thus be a lingering effect of sustained armed conflicts, but it should not always be justified as an obstacle to nature conservation. Although the mammal fauna of Quiçama is exposed to mounting perils, such as human population growth, deforestation and spread of exotic species, bushmeat exploitation for commerce is undoubtedly the most imperative threat. We therefore argue for the imminent implementation of activities that can ensure both wildlife recovery, and food security and egalitarian rights for the local human communities. These efforts should include environmental education, greater human rights and social equality, and the understanding and integration of legal anti-poaching instruments and hunting management. Given the frequent engagement of residents with hunting and the highly

scenic landscape value of the study area, we strongly encourage community-based ecotourism and wildlife management activities. In addition, considering that local ecological knowledge can efficiently strengthen management and conservation guidelines, we also highlight the importance of employing local villagers as wildlife guides and including them in official wildlife management and monitoring plans. We also draw attention to the need to implement adequate anti-poaching enforcement and full-time presence of park staff in the central and southern portions of Quiçama, especially in areas ~~of~~exposed to the most intensive hunting activity and along roads through which the wildlife trade operates. Finally, the routes that link Quiçama to large cities must be monitored and transformed from traffic routes into highways that enable sustainable activities that capitalize on the regional ecological and cultural diversity, such as educational endeavors for school kids and ecotourism.

Acknowledgements

We sincerely thank the people from local communities who shared their knowledge on wildlife and supported us in data collection through opportunist observations. Without them, this work would not be possible. We would like to thank the Environmental Ministry of Angola, Quiçama municipal administration, Quiçama Park administration and all village coordinators for authorizing and support this research. We thank the Faculdade de Ciências of Universidade de Agostinho Neto (Angola) and The Rufford Foundation for the financial support. F-BP thanks the Coordenação de Aperfeiçoamento Pessoal de Nível Superior (CAPES) for the master scholarship. RRNA thanks National Council for Scientific and Technological Development by a productivity research grant. We thank the reviewer for the important contribution to this manuscript.

REFERENCES

1. Bordes, F., Thibault C. Thoughts on the initial adaptation of Hominids to European glacial climates. Quat Res [Internet]. 1997; Available from: https://doi.org/10.1016/0033-5894(77)90059-X
2. Darimont, C.T., Fox, C.H., Bryan, H.M., Reimchen TE. The unique ecology of human predators. Science (80-) [Internet]. 2015; Available from: https://doi.org/10.1126/science.aac4249
3. Alves, R.R.N., Silva, J.S., Chaves, L.S., Albuquerque UP. Ethnozoology and Animal Conservation. In: Alves, R.R.N., Albuquerque UP, editor. Ethnozoology: animals in our lives. Academic Press. Elsevier, London,; 2018. p. 481–496.
4. Peres, C.A. Effects of hunting on western Amazonian primate communities. Biol Conserv. 1990;54(1):47–59.
5. Ripple WJ, Abernethy K, Betts MG, Chapron G, Dirzo R, Galetti M, et al. Bushmeat hunting and extinction risk to the world's mammals. R Soc Open Sci [Internet]. 2016;3(10):160498. Available from: http://rsos.royalsocietypublishing.org/lookup/doi/10.1098/rsos.160498
6. Benítez-López, A., Santini, L., Schipper, A. M., Busana, M., & Huijbregts MAJ. Intact but empty forests? Patterns of hunting-induced mammal defaunation in the tropics. PLOS Biol [Internet]. 2019;17(5). Available from: 10.1371/journal.pbio.3000247
7. Peres, C.A. and Lake IR. Extent of nontimber resource extraction in tropical forests: accessibility to game vertebrates by hunters in the Amazon basin. Conserv Biol. 2003;17(2):521–35.
8. Bogoni JA, Peres CA, Ferraz KMPMB. Extent, intensity and drivers of mammal defaunation: a continental-scale analysis across the Neotropics. Sci Rep [Internet]. 2020 Dec 15;10(1):14750. Available from: http://www.nature.com/articles/s41598-020-72010-w
9. Effiom, E.O., G. Nuñez-Iturri, H.G. Smith UO& OO. Bushmeat hunting changes regeneration of African rainforests. Proc R Soc B Biol Sci. 2013;280(1759):1–8.
10. Nasi, R., A. Taber and N van V. Empty forests, empty stomachs? Bushmeat and

Formatted: Line spacing: 1.5 lines

Formatted

livelihoods in the Congo and Amazon Basins. Int For Rev. 2011;13(3):355–68.
11. Leopold AS. Wildlife of Mexico: The Game Birds and Mammals. Berkeley and Los Angeles: Univ of California Press; 1959.
12. Redford, K.H., Robinson JG. The game of choice: patterns of indian and colonist hunting in the Neotropics. Am Anthr. 1987;89:650–667.
13. Cardillo M, Cardillo M, Mace GM, Jones KE, Bielby J, Bininda-Emonds ORP, et al. Multiple Causes of High Extinction Risk in Large Mammal Species. Science (80-) [Internet]. 2005;309(5738):1239–41. Available from: http://www.ncbi.nlm.nih.gov/pubmed/16037416%5Cnhttp://www.sciencemag.org/content/309/5738/1239%5Cnhttp://www.sciencemag.org.proxy.bibliotheques.uqam.ca:2048/content/309/5738/1239.abstract?ijkey=1c808d0dc7686f93d6eb0d6c1352429f2390e3d2&keytype2=tf_ipsecsha
14. Braga-Pereira F, Peres CA, Campos-Silva JV, Santos CV-D, Alves RRN. Warfare-induced mammal population declines in Southwestern Africa are mediated by species life history, habitat type and hunter preferences. Sci Rep. 2020;10(1):1–15.
15. Di Marco M, Boitani L, Mallon D, Hoffmann M, Iacucci A, Meijaard E, et al. A Retrospective evaluation of the global decline of carnivores and ungulates. Conserv Biol. 2014;28(4):1109–18.
16. Dirzo R, Young HS, Galetti M, Ceballos G, Isaac NJB, Collen B. Defaunation in the Antrhopocene. Science (80-). 2014;401(6195):401–6.
17. Davidson AD, Hamilton MJ, Boyer AG, Brown JH, Ceballos G. Multiple ecological pathways to extinction in mammals. Proc Natl Acad Sci [Internet]. 2009;106(26):10702–5. Available from: http://www.pnas.org/cgi/doi/10.1073/pnas.0901956106
18. Pac, D. F., & White GC. Survival and Cause-Specific Mortality of Male Mule Deer Under Different Hunting Regulations in the Bridger Mountains, Montana. J Wildl Manage. 2007;71(3):816–827.
19. Corlatti, L., Storch, I., Filli F and PA. Does selection on horn length in males and females differ between protected and hunted populations of a weakly dimorphic

ungulate? Ecol Evol. 2017;7:3713–3723.

20. Bugir, C.K.; Peres, C.A.; White, K.S.; Montgomery, R.A.; Griffin, A.S.; Rippon, P.; Clulow, J.; Hayward MW. Prey preferences of modern human hunter-gatherers. Food Webs. 2021;26.
21. Abernethy, K. A., Coad, L., Taylor, G., Lee, M. E., & Maisels F. Extent and ecological consequences of hunting in Central African rainforests in the twenty-first century. Philos Trans R Soc B Biol Sci. 2013;368(1625).
22. Bowler M, Beirne C, Tobler MW, Anderson M, Dipaola A, Fa JE, et al. LED flashlight technology facilitates wild meat extraction across the tropics. 2020;489–95.
23. Dudley JP, Ginsberg JR, Plumptre AJ, Hart J a, Campos LC. Effects of War and Civil Strife on Wildlife and Wildlife Habitats\nEfectos de la Guerra y Conflictos Civiles Sobre la Vida Silvestre y Sus Hábitats. Conserv Biol [Internet]. 2002;16(2):319–29. Available from: http://dx.doi.org/10.1046/j.1523-1739.2002.00306.x
24. Duda R. Hunting Techniques, Wildlife Offtake and Market Integration. A Perspective from Individual Variations among the Baka (Cameroon). The Center for African Area Studies, Kyoto University, Japan.; 2017.
25. Milner-Gulland EJ. Interactions between human behaviour and ecological systems. Philos Trans R Soc B Biol Sci. 2012;367(1586):270–278.
26. Muth, R. M., & Bowe JF. Illegal harvest of renewable natural resources in North America: Toward a typology of the motivations for poaching. Soc Nat Resour. 1998;11(1):9–24.
27. Bowen-Jones, E., Brown, D., & Robinson EJZ. Economic commodity or environmental crisis? An interdisciplinary approach to analysing the bushmeat trade in central and west Africa. Area. 2003;35(4):390–402.
28. Knapp EJ, Peace N BL. Poachers and Poverty: Assessing Objective and Subjective Measures of Poverty among Illegal Hunters Outside Ruaha National Park, Tanzania. Conserv Soc. 2017;15:24–32.
29. Mateo-Tomás, P., Olea, P. P., Sánchez-Barbudo, I. S., & Mateo R. Alleviating

human-wildlife conflicts: identifying the causes and mapping the risk of illegal poisoning of wild fauna. J Appl Ecol. 2012;49(2):376–385.

30. Bakarr M. OW and AE. West Africa: regional overview of the bushmeat crisis. In BCTF Collaborative Action Planning Meeting Proceedings. In: N. D. Bailey, H. E. Eves AS and JTS, editor. 2001. Silver Spring, MD;
31. Fa JE, Peres CA, Meeuwig J. Bushmeat Exploitation in Tropical Forests: an Intercontinental Comparison. Conserv Biol [Internet]. 2002 Feb;16(1):232–7. Available from: http://doi.wiley.com/10.1046/j.1523-1739.2002.00275.x
32. Braga-Pereira F, Santoro FR, Van-Duném Santos C, Alves RRN. First record of Angola's medicinal animals: A case study on the use of mammals in local medicine in quiçama national park. Indian J Tradit Knowl. 2017;16(4).
33. Wittemyer, G., Daballen, D. & Douglas-Hamilton I. Poaching prices: rising ivory prices threaten elephants. Nature. 2011;476:282–3.
34. Challender, D. W. S., & MacMillan DC. Poaching is more than an Enforcement Problem. Conserv Lett. 2014;7(5):484–494.
35. Pringle RM. Upgrading protected areas to conserve wild biodiversity. Nature. 2017;
36. Ministério do Urbanismo e Ambiente. Primeiro Relatório Nacional para a Conferência das Partes da Convenção da Diversidade Biológica [Internet]. 2006. Available from: https://www.cbd.int/doc/world/ao/ao-nr-01-pt.pdf
37. Liebenberg, A., Basterfield, P., Daehnke, A., Rogery GR. Report on the Elephants Relocated to the Quiçama National Park – Angola. University of Pretoria; 2001.
38. Bailey K. Methods of social research. 4th ed. The Free Press, New York; 1994.
39. H.R. B. Research methods in cultural anthropology. 1998. 200 p.
40. Braun V, Clarke V. Using thematic analysis in psychology. Qual Psychol Res. 2006;3(2):77–101.
41. JONES KE, BIELBY J, CARDILLO M, FRITZ SA, O'DELL J, ORME CDL, et al. PanTHERIA : a species-level database of life history , ecology , and geography of extant and recently extinct mammals. 2009;90(9).
42. Wilman, H., Belmaker, J., Simpson, J., de la Rosa, C., Rivadeneira, M. M., & Jetz

W. EltonTraits 1.0: Species-level foraging attributes of the world's birds and mammals. Ecology. 2014;95(7):2027–2027.

43. Bogoni, J. A., Silva, P. G. da, & Peres CA. Co‐declining mammal‐dung beetle faunas throughout the Atlantic Forest biome of South America. Ecography (Cop). 2019;

44. Tacutu, R., Thornton, D., Johnson, E., Budovsky, A., Barardo, D., Craig, T., Diana, E., Lehmann, G., Toren, D., Wang, J., Fraifeld, V. E., de Magalhaes JP. Human Ageing Genomic Resources: new and updated databases. Nucleic Acids Res. 2018;

45. ERNEST SKM. LIFE HISTORY CHARACTERISTICS OF PLACENTAL NONVOLANT MAMMALS Ecological. Ecol Soc Am. 2003;84(12):3402–2403.

46. Harrison, X. A., Donaldson, L., Correa-Cano, M. E., Evans, J., Fisher, D. N., Goodwin, C. E., ... & Inger R. A brief introduction to mixed-effects modelling and multi-model inference in ecology. PeerJ. 2018;6.

47. Husson F, Josse J, Le S, Mazet J. FactoMineR: multivariate exploratory data analysis and data mining. J Stat Softw [Internet]. 2018;25:1–18. Available from: https://rdrr.io/cran/FactoMineR/man/FactoMineR-package.html

48. Oksanen J, Blanchet F, Kindt R, Legendre P, Others, Other, et al. vegan:community ecology package. 2013.

49. Whitfield J. The law of the jungle. Nature. 2003;421(6918):8–9.

50. Schlossberg S, Chase MJ GC. Poaching and human encroachment reverse recovery of African savannah elephants in south-east Angola despite 14 years of peace. PLoS One. 2018;13(3).

51. AA B. The contribution of landmines to land degradation. L Degrad Dev. 2007;18:1–15.

52. Fa JE, Peres CA. Game vertebrate extraction in African and Neotropical forests: an intercontinental comparison. Conserv Exploit Species. 2001;203–41.

53. Fa, J. E., Ryan, S. F., & Bell DJ. Hunting vulnerability, ecological characteristics and harvest rates of bushmeat species in afrotropical forests. Biol Conserv. 2005;121(2):167–176.

54. Braga-Pereira F., Bogoni JA., Alves RRN. From spears to automatic rifles: The shift in hunting techniques as a mammal depletion driver during the Angolan civil war. Biol Conserv. 2020;249.
55. MacMillan, D.C. & Nguyen AQ. Factors influencing the illegal harvest of wildlife by trapping and snaring. 2013;
56. Shrestha, Suresh K.; Burns RC. Big game hunting practices, meanings, motivations and constraints: a survey of Oregon big game hunters. In: Fisher, Cherie LeBlanc; Watts, Clifton E. J, editor. Proceedings of the 2010 Northeastern Recreation Research Symposium. US; 2012. p. 1–8.
57. Milner, J.M., Nilsen, E.B., Amdreassen HP. Demographic side effects of selective hunting in ungulates and carnivores. Conserv Biol. 2007;
58. Milner-Gulland G. Sex-biased harvesting and population dynamics in ingulates: implications for conservation and sustainable use. Conserv Biol. 1994;
59. Darimont CT, Carlson SM, Kinnison MT, Paquet PC, Reimchen TE, Wilmers CC. Human predators outpace other agents of trait change in the wild. Proc Natl Acad Sci [Internet]. 2009;106(3):952–4. Available from: http://www.pnas.org/cgi/doi/10.1073/pnas.0809235106
60. Speth JD. Big-Game Hunting: Protein, Fat, or Politics? In: The Paleoanthropology and Archaeology of Big-Game Hunting Interdisciplinary Contributions to Archaeology. Springer, New York, NY; 2010.
61. Petracca, L. S.; Funston, P. J.; Henschel, P.; Cohen, J. B.; Maclennan, S.; Frair JL. Modeling community occupancy from line transect data: a case study with large mammals in post- war Angola. Anim Conserv. 2019;
62. Borgerhoff-Mulder M and PC. Conservation: linking ecology, economics, and culture. New Jersey Princet Univ Press. 2005;
63. Gao, Y., & Clark SG. Elephant ivory trade in China: Trends and drivers. Biol Conserv. 2014;180:23–30.
64. Lindsey, P.A., Balme, G., Becker, M., Begg. C., Bento, C., Bocchino, C., Dickman. A., Diggle, R.W., Eves, H., Henschel, P., Lewis . D., Marnewick , K., Mattheus, J., McNutt, W., McRobb, R., Midlane , N., Milanzi, J., Morley , R., Murphree , M.,

Opyene , V P. The bushmeat trade in African savannas: Impacts, drivers, and possible solutions. 2012.

65. Lindsey, P., Taylor, W.A., Nyirenda, V., Barnes L. Bushmeat, wildlife-based economies, food security and conservation: Insights into the ecological and social impacts of the bushmeat trade in African savannahs [Internet]. FAO. 2015. p. 58. Available from: FAO/Panthera/Zoological Society of London/SULi Report, Harare

66. Daskin JH, Pringle RM. Warfare and wildlife declines in Africa’s protected areas. Nature [Internet]. 2018;553(7688):328–32. Available from: http://dx.doi.org/10.1038/nature25194

Formatted: Line spacing: 1.5 lines

Angola, July 2021.

From: Franciany B-P.
Franbraga83@yahoo.com.br

To: Emmanuel Serrano
Academic Editor of Plos One

Dear Editor,

We are submitting a new version of our manuscript (ID: **PONE-D-21-09230**) entitled "**Intrinsic and extrinsic motivations governing prey choice by hunters in a post-war African forest-savannah macromosaic**"**.** We would like to thank the reviewer for the comprehensive comments and suggestions on the paper. We appreciate the feedback and believe that responding to these comments have improved the quality of our manuscript.
Please find below a point-by-point response to the comments provided by the Reviewer. We are providing two versions of the Manuscript, one with and other without track changes, so that you can see exactly what has been altered. Note that the lines indicated below is referring to the **clean version** of the Manuscript.

Yours sincerely

Franciany G. Braga-Pereira (on behalf of all co-authors)

Journal: Plos One

Ref:PONE-D-21-09230

Reviewer #1: The main merits of this manuscript are (1) using local knowledge to provide an interesting, useful and easy methodology to (2) estimate the motivations of hunters in a post-war area. In many places worldwide, the only data available for management and conservation proposes is the ecological local knowledge (besides hunting bags when available), therefore any method focused on using this knowledge to produce population estimates (management) and the motivations for killing certain species (conservation) will be proven very helpful, especially when referring to natural areas or countries with strong budget limitations or low-income economies.

As a matter of fact, it is impossible to change a person's behavior towards wildlife (e.g., feeding or poaching) without knowing the motivations behind that behaviour. This study addresses a stated need of new strategies which, based on the motivations towards hunting certain species, help to better plan for the management of ecosystems that are recovering from strong disturbances, such as wars, and where poaching is present with strong impact on the recovery of wildlife populations.

This is a sound-based manuscript; however, I have some remarks on the text.

Authors: We are very grateful for these positive comments and effort to improve our manuscript. We followed all of your suggestions and we believe that our manuscript has been further strengthened after these recommendations.

Reviewer #1

L79-82: I may not have understood this part of the manuscript correctly, so in that case the authors should state it clearer, but when asking about the motivation behind the pursuit each wild mammals during the semi-structured individual interviews, did the authors used a closed questionary based on Muth and Bowe 1998, and the rest of the intrinsic, extrinsic and individual based

motivations mentioned in the manuscript, or did you allowed the hunters to relate their motivations without any “constrain”?
I ask this question since you the authors had the opportunity to collect first-hand the motivations of hunters in a post-war area, I think that the appearance of unpredicted motivations could have had a great importance, both for the results and for the impact of the present manuscript.

Authors: We agree with your comments regarding the opportunity to first-hand collect the motivations of hunters and we confirmed we allowed the hunters to relate their motivations without any “constrain”. We had improve the main manuscript for a better explanation on that (lines 186-199) and we provided the whole questionnaire used in this study in the supplementary material.

Reviewer #1 Moreover, I think the discussion of the manuscript could be improved if the results of this study were compared with other studies, stating similarities and differences with the motivations of poachers in other post-war areas (if any, and ideally in other continents), or in non-post-war areas, such as the study cited Muth and Bowe 1998, in the USA, where the conditions for poachers were totally different.

Authors: We agree with you and had improved the discussion on that (lines 452-460; 510-514; 521-545)

Reviewer 1# L211 Data analysis. I have several concerns about the data analysis: The authors interviewed 115 hunters with different ages (19-80 years old), from eight settlements (one located in the Game reserve), with different population densities and located in different ecosystems (forest-savannah). If I was a manager of this or a similar natural area wanting to implement conservation strategies based on working with the communities, I would find attractive your study but after reading the manuscript, several questions would come to my mind. Are the motivations for hunting the same along all ages and settlements regardless of their differences? Besides stating the differences in the

activity among male and female and adults, children and the elderly, the manuscript would be improved if the authors evaluated possible differences in the motivations due to the hunters' characteristics.

As the authors did not evaluated differences due to hunters' characteristics, why did the authors used a GLM instead of a GLMM, where the hunter and the settlement were included as nested random effects? The results would be more robust.

But as I stated before, I think that the authors are not taking full advantage of what they could get from the data. In my opinion and based on my experience, to study the motivations of human behaviors towards wildlife based on surveys, the multivariate statistical tests can give you more information, especially those based on the canonical ordination, such as multiple factor analysis (http://www.sthda.com/english/articles/31-principal-component-methods-in-r-practical-guide/116-mfa-multiple-factor-analysis-in-r-essentials/).

Authors: We appreciated this comment. We had performed a MFA for better understand the similarities of species selected to attend different hunting motivations regarding species body mass and energetic level; sampled site; and interviewee age and sex.

In addition, to examine the effects of species body mass and trophic level; and interviewee age, gender and natal community on the number of hunters reporting each hunting motivation for killing any given species we performed generalized linear mixed models (GLMM) and generalized linear models (GLM). We performed a GLMM considering the interviewee as a random variable; and the body mass and energetic level as fixed predict variable.

We assigned the 8 sampled communities into four study sites according to type of landscape structure (forest of savannah) and geographic position. We therefore sampled two communities in savannah areas of northern Quiçama; two in savannah areas of southern Quiçama; two in central forest areas; and two communities in southern forest areas. By this way we could better explain our results for readers not familiar to the area and in the same time to ensure the interviewee confidentiality, as some communities contained few hunters.

See lines in the Methods section: 217-228; 232-249; 260-272.

See lines in the Results section: 313-328; Table 1 (355); 396-409.

See lines in the Discussion section: 489-509; 562-567.

See Supplementary Material: Figures 2 and 3.

Reviewer 1#L44-46: I recommend to review the citations in text and references. Just as an example I found two mistakes between L82 and L86 (e.g. In L82 you cited (Muth and Bowe 1998) but I cannot find the citation in references, L86-87 you cited as (Knapp and Peace 2017) but the article has three authors.

Authors: Thank you for your comment. We have reviewed the citations and references.

Reviewer1# L199-201: Again, I may not have understood this part correctly, if so, the authors should state it clearer. In the example: "if the grazer/frugivore Tragelaphus oryx consumes 70% grass and 30% fruit, its mean trophic energy level would be 2.7 (= (0.7 × 3) + (0.3 × 2)", but according to the levels in L195-197 ((1) grazer/folivore < (2) frugivore < (3) granivore…) the level should be 1.3 (= (0.7 × 1) + (0.3 × 2).

Authors: Thank you for your comment. We corrected the text (line 203-208).

Reviewer 1#L343-367: The authors should state the usefulness of ecological local knowledge as a tool to estimate wildlife abundance.

Authors:

Thank you for your comment. We agree and had edited the text (lines:621-624).
