# Supplementary material for: Intrinsic and extrinsic motivations governing prey choice by hunters in a post-war African forest-savannah macromosaic
Source: PLoS One. 2021 Dec 20;16(12):e0261198. doi: 10.1371/journal.pone.0261198 (PMC8687528; doi:10.1371/journal.pone.0261198)
Supplement: Attachment — Submitted filename: AIC model selection, example.pdf [file pone.0261198.s008.pdf]

DOI: 10.7589/2014-01-008 *Journal of Wildlife Diseases,* 51(1), 2015, pp. 148–156
© Wildlife Disease Association 2015

DISTINGUISHING DISEASE EFFECTS FROM ENVIRONMENTAL EFFECTS IN A MOUNTAIN UNGULATE: SEASONAL VARIATION IN BODY WEIGHT, HEMATOLOGY, AND SERUM CHEMISTRY AMONG IBERIAN IBEX (*CAPRA PYRENAICA*) AFFECTED BY SARCOPTIC MANGE

Jesús M. Pérez,[1,8] Emmanuel Serrano,[2,3] Ramón C. Soriguer,[4] Francisco J. González,[5] Mathieu Sarasa,[1] José E. Granados,[6] Francisco J. Cano-Manuel,[6] Rafaela Cuenca,[2] and Paulino Fandos[7]

[1] Departamento de Biología Animal, Biología Vegetal y Ecología, Universidad de Jaén, Campus Las Lagunillas, s.n., E-23071, Jaén, Spain
[2] Servei d'Ecopatologia de Fauna Salvatge (SEFaS), Departament de Medicina i Cirurgia Animals, Universitat Autònoma de Barcelona, E-08193, Bellaterra, Barcelona, Spain
[3] CESAM, Departamento de Biologia, Universidade de Aveiro, Campus Universitario de Santiago, 3810-193, Aveiro, Portugal
[4] Estación Biológica de Doñana (CSIC), Av. Américo Vespucio, s.n., E-41092 Sevilla, Spain
[5] Laboratorio PROLAB, Carrera 51, E-23600, Martos, Jaén, Spain
[6] Espacio Natural Sierra Nevada, Carretera Antigua de Sierra Nevada, Km 7, E-18071, Pinos Genil, Granada, Spain
[7] Agencia de Medio Ambiente y Agua, Isla de la Cartuja, E-41092, Sevilla, Spain
[8] Corresponding author (email: jperez@ujaen.es)

ABSTRACT: Our study focuses on the Iberian ibex (*Capra pyrenaica*) from the Sierra Nevada Natural Space (southern Spain), where sarcoptic mange is an endemic disease and animals are affected by a highly seasonal environment. Our aim was to distinguish between disease and environmental influences on seasonal variation in body weight, hematology, and serum biochemistry in Iberian ibex. We sampled 136 chemically immobilized male ibexes. The single effect of mange influenced hemoglobin, hematocrit, mean corpuscular volume, leukocytes, band neutrophils, monocytes, cholesterol, urea, creatine, and aspartate aminotransferase. Both mange and the period of the year also affected values of mean corpuscular hemoglobin, mean corpuscular hemoglobin concentration, neutrophils, glucose, and serum proteins. Scabietic animals showed a marked reduction in body weight (21.4 kg on average), which was more pronounced in winter. These results reveal that 1) infested animals are anemic, 2) secondary infections likely occur, and 3) sarcoptic mange is catabolic.
Key words: *Capra pyrenaica*, host-parasite relationships, *Sarcoptes scabiei*, wildlife diseases.

INTRODUCTION

Sarcoptic mange caused by *Sarcoptes scabiei* is responsible for epizootic disease in free-ranging populations of a broad range of wild mammals around the globe (Pence and Ueckermann 2002). Hematologic and biochemical responses to mange are relatively well known in domestic and wild mammals (Sarasa et al. 2010; Akomas et al. 2011; Kido et al. 2011). However, physiologic response of animals affected by mange in highly seasonal mountain ecosystems has not been evaluated. Using a sample of 136 male Iberian ibexes (*Capra pyrenaica*) from the Sierra Nevada Natural Space (southern Spain), we assessed whether mange affects body weight, which shows markedly seasonal variations, but also examined the effects of mange on hematologic and serum chemistry values of affected animals. Because ibexes in the Sierra Nevada experience strong seasonal changes in body reserves (Serrano et al. 2011) and such body stores are correlated to some hematologic parameters (Serrano et al. 2008), we expected that mange may drive the seasonal hematologic profile of this mountain ungulate.

MATERIALS AND METHODS

Study area

We studied the Iberian ibex population of the Sierra Nevada Natural Space (36°00′–37°10′N, 2°34′–3°40′W, southern Spain). This

TABLE 1. Mean±SE, sample size (applicable to all tables), and range (in parentheses) of body weights (kg) of 136 male Iberian ibex, *Capra pyrenaica*, >3 yr old, captured in the Sierra Nevada Natural Space, southern Spain, 2005–12. WS indicates ibexes captured in winter and spring and SA in summer and autumn. Numbers in columns represent four categories based on the percentage of skin surface affected by mange.

	Mange status[a]			
	0	1	2	3
WS				
Mean±SE (range)	53.5±1.4 (20–72)	52±2.2 (35–72)	46.2±2.3 (32–59)	36.3±5.1 (28–54)
n	47	19	14	5
SA				
Mean±SE (range)	64.6±1.7 (32–75)	56.8±2.7 (50–65)	45.7±3.5 (36–52)	38.8±1.6 (34–46)
n	34	6	4	7

[a] 0 = ibex without skin lesions; 1 = skin surface affected <25%; 2 = skin surface affected between 25 and 50%; 3 = skin surface affected >50%.

area comprises the alpine massif, including the highest peaks in the Iberian Peninsula (e.g., the Mulhacén, 3,481 m above sea level). This area is characterized by strong seasonal climatic variations; snow cover is present for 6 mo (December to May), and most plant growth occurs from June to August (Peinado and Rivas-Martínez 1987).

Ibex capture and blood collection

Between 2005 and 2012 blood samples from 136 male Iberian ibexes 3–11 yr old (81 clinically healthy and 55 scabietic animals) were collected. Animals were chemically immobilized with a mixture of xylazine (3 mg/kg) and ketamine (3 mg/kg; Casas-Díaz et al. 2011) and aged by growth segments on the horns (Fandos 1991). During immobilization we collected blood into tubes containing ethylenediaminetetraacetic acid tripotassium and serum tubes. Time required for blood collection was similar for all animals with a mean of 5 min (range, 3–15 min).

Scabietic animals were visually assigned to four categories according to the percentage of skin surface affected by mites (Pérez et al. 2011: 0 = ibexes without skin lesions; 1 = skin surface affected <25%; 2 = skin surface affected 25–50%; and 3 = skin surface affected >50%). Skin scrapings were analyzed at the laboratory to confirm the diagnoses.

Hematology

Whole blood samples were stored at 4 C until arrival at the laboratory. Hematologic analyses were performed within 24 hr of collection. Clotted tubes were centrifuged at 4,750 × G for 10 min, and sera were stored at −20 C until analysis. Red blood cell count (RBC), hemoglobin concentration (Hb), hematocrit, mean corpuscular volume (MCV), mean corpuscular hemoglobin (MCH), mean corpuscular hemoglobin concentration (MCHC), white blood cell count, and differential leukocyte count were determined with a Cell-Dyn 3500® autoanalyzer (Abbott Científica, S.A., Madrid, Spain).

Biochemistry

We measured: glucose, cholesterol, urea, creatine, aspartate aminotransferase (AST), creatine kinase (CK), lactate dehydrogenase (LDH), total proteins, and albumin using an autoanalyzer (BT 2245®, Biotechnica Instruments, Rome, Italy). Total proteins were measured using the Biuret method (BioSystems®–Atom, Barcelona, Spain), and serum proteins were separated using a power supply BTS-100 (Biosystems, S.A, Barcelona, Spain) on cellulose acetate strips (CellogeL®, Milan, Italy) and sodium veronal 0.04 M sodium diethylbarbiturate 8.25 *g/L* as a buffer at 220 V for 38 min. Bands obtained were stained with amido black and quantified using a 434 photodensitometer® (Digiscan, Barcelona, Spain).

Statistical analyses

We evaluated whether the interaction between mange infection and seasons influenced body weight of ibexes using linear models (LM). Four categories of mange infection were considered. The effects of seasons were evaluated in two periods of the year that represent the main seasonal changes of body stores (Serrano et al. 2011): winter–spring (15 December–15 June) and summer–autumn (16 June–14 December). To control for potential

TABLE 2. Mean±SE (range) of values of hematologic parameters in 136 male Iberian ibex, *Capra pyrenaica*, >3 yr old, captured in the Sierra Nevada Natural Space, southern Spain, 2005–12. WS indicates ibexes captured in winter and spring and SA in summer and autumn.

Parameter[a]	Mange status 0	1	2	3
RBC (10^{12}/L)				
WS	14.63±0.23 (10.40–18.11)	14.41±0.35 (10.80–16.5)	13.14±0.51 (9.19–14.8)	11.44±0.69 (9.32–13.3)
SA	14.97±0.37 (8.5–20.6)	13.93±0.46 (12.7–15.8)	12.63±0.61 (11.8–13.8)	12.96±0.64 (11.00–14.7)
Hemoglobin (g/dL)				
WS	147.63±2.34 (106.0–171.0)	156.05±6.08 (121.0–224.0)	134.35±4.96 (92.0–153.0)	118.31±7.98 (95.0–138.0)
SA	156.73±4.22 (84.2–200.0)	140.16±7.35 (124.0–173.0)	127.66±5.36 (120.0–138.0)	125.61±7.46 (104.0–145.0)
Hematocrit (%)				
WS	38.07±0.47 (31.0–44.0)	36.51±0.81 (32.0–43.0)	34.95±0.81 (29.0–40.0)	34.44±1.72 (29.0–39.0)
SA	38.19±0.84 (26.1–49.0)	36.06±1.27 (32.3–41.6)	35.46±0.24 (35.0–35.8)	34.22±1.71 (29.9–39.0)
MCH (pg)				
WS	10.26±0.07 (8.9–11.3)	10.53±0.21 (9.4–13.1)	10.29±0.14 (9.5–11.3)	10.23±0.17 (9.7–10.7)
SA	10.43±0.14 (8.6–12.99)	10.03±0.19 (9.69–10.95)	9.84±0.18 (9.52–10.16)	9.67±0.19 (9.19–10.31)
MCV (fL)				
WS	26.82±0.36 (22.9–32.1)	25.28±0.59 (22.6–30.7)	26.62±0.99 (23.7–36.1)	30.62±0.53 (29.36–32.16)
SA	25.29±0.39 (17.8–30.7)	25.91±0.56 (23.93–27.95)	27.02±1.09 (25.4–29.11)	26.43±0.61 (24.63–28.13)
MCHC (%)				
WS	38.44±0.47 (33.5–44.4)	41.99±1.25 (34.4–53.3)	38.62±0.94 (31.15–43.3)	33.3±0.26 (32.48–34.1)
SA	41.66±0.73 (32.26–52.63)	38.81±1.11 (34.93–41.59)	36.51±0.81 (34.92–37.51)	36.64±0.61 (34.78–38.46)
Leukocytes/mL				
WS	8,179.21±418.41 (2,860–13,600)	9,804.7±472.91 (6,960–13,500)	11,190±1,137.89 (1,990–17,500)	19,340.21±1,478.79 (14,600–21,701)
SA	9,674.54±494.85 (3,370–15,800)	10,376.66±1,687.08 (5,880–17,500)	12,866.67±1,373.96 (10,300–15,000)	12,482.0±2,282.13 (6,210–20,200)
Bands/mL				
WS	367.21±57.93 (0–1,428)	427.64±70.01 (93–1,080)	711.58±142.77 (20–1,750)	1,873.41±260.85 (1,080–2,388)
SA	280.48±38.45 (0–810)	379.66±154.23 (118–1,000)	677.66±38.62 (618–750)	868.0±450.32 (662–2,626)
Neutrophils/mL				
WS	3,470.31±224.59 (1,316–8,568)	4,057.71±340.04 (2,220–6,750)	5,296.33±543.27 (995–8,259)	13,497.81±1,861.07 (7,866–16,710)
SA	2,804.80±212.84 (1,370–6,812)	3,796.60±1,427 (3,302–12,600)	6,776.60±1,953.61 (4,944–11,700)	8,680.00±1,653.74 (994–9,912)

TABLE 2. Continued.

Parameter[a]	Mange status			
	0	1	2	3
Eosinophils/mL				
WS	525.28±82.08 (0–2,499)	704.05±99.92 (0–1,386)	901.0±310.78 (0–3,850)	371.41±335.83 (0–1,710)
SA	369.72±56.93 (0–1,314)	438.33±190.79 (0–1,180)	810.61±132.27 (0–1,064)	327.0±140.78 (0–816)
Lymphocytes/mL				
WS	3,553.21±265.85 (404–8,250)	4,348.64±573.41 (3,322–6,048)	4,073.75±573.41 (577–6,630)	2,752.21±782.02 (1,085–4,788)
SA	4,302.48±347.61 (2,234–8,456)	3,400.16±346.96 (2,234–4,720)	2,894.33±631.91 (1,650–3,708)	3,548.81±870.98 (944–6,060)
Monocytes/mL				
WS	145.26±60.84 (0–2,288)	163.71±26.23 (0–476)	118.16±41.57 (0–525)	739.61±329.18 (0–1,520)
SA	401.39±46.06 (0–1,053)	479.66±110.32 (59–875)	197.33±56.04 (133–309)	475.0±146.23 (212–1,010)

[a] RBC = red blood cell count; MCH = mean corpuscular hemoglobin; MCV = mean corpuscular volume; MCHC = mean corpuscular hemoglobin concentration.

effects of age, only ibexes close to reaching full body development were included in our analyses (>3 yr old; Serrano et al. 2004). The mean age of ibexes did not significantly differ among categories of mange infection ($F=1.525$, $P=0.2$).

We evaluated the physiologic response of ibexes to mange using multivariate analysis of variance (MANOVA). Our sampling design included a balanced proportion of sick and healthy ibexes in both periods of the year (Kruskal-Wallis=3.6, $P=0.06$), minimizing the possibility of spurious results.

For both LM and MANOVA, a model selection procedure based on the Akaike Information Criterion corrected for small sample size (AICc) was performed (Burnham and Anderson 2002). The model with the lowest AICc was retained, and the remaining competing models were ordered according to their Akaike differences (Δi) with respect to the best model (lowest AICc). The Akaike weight ($W_{i,}$) and the percentage of explained variability (R^2) for the best model were also estimated. Statistical analyses were carried out in the statistical software R version 3.0.3 (R Development Core Team 2014).

RESULTS

Descriptive statistics for body weight and hematologic and serum biochemical parameters studied are summarized in Tables 1–3. Our model selection suggested a seasonal-dependent effect of mange on ibex weight ($w_{\text{Mange status*Period of the year}}=0.52$; Table 4). However, and according to the principle of parsimony, the second competing model ($\Delta_i=0.17$), which suggested the additive effects of both factors, would have more support ($w_{\text{Mange status+Period of the year}}=0.47$; Table 4). The model suggests that 40% of the observed body weight variability relied on mange infection and period of the year. Reduced body weight in scabietic ibexes appeared in the early stages of infection ($\beta_{\text{2nd mange category}}=-10.19$, SE=2.5, $t=-4.01$) but peaked when more than half of the skin surface was affected ($\beta_{\text{3rd mange category}}=-21.47$, SE=2.9, $t=-7.21$). During the course of infection, weight loss in ibexes ranged between 32% (17 kg) in winter and 39.9% (25.8 kg) in summer (Table 1). Winter conditions also

TABLE 3. Mean±SE (range) of values of serum biochemical parameters in 136 male Iberian ibex, *Capra pyrenaica*, over 3 yr of age, captured in the Sierra Nevada Natural Space, southern Spain, 2005–12. WS indicates ibexes captured in winter and spring and SA in summer and autumn.

	Mange status			
Parameter[a]	0	1	2	3
Glucose (mmol/L)				
WS	3.36±0.33 (0.15–9.2)	3.17±0.51 (0.22–7.17)	3.41±0.79 (0.88–11.6)	0.51±0.19 (0.17–1.27)
SA	5.46±0.58 (0.27–13.65)	2.53±0.66 (0.22–4.27)	0.79±0.29 (0.27–1.65)	2.81±1.54 (0.11–10.76)
Cholesterol (mmol/L)				
WS	1.11±0.03 (0.77–1.65)	1.05±0.05 (0.64–1.57)	1.06±0.06 (0.38–1.49)	0.82±0.13 (0.51–1.18)
SA	1.33±0.04 (0.82–2.09)	1.37±0.08 (1.16–1.65)	1.35±0.11 (1.07–1.64)	1.15±0.11 (0.74–1.61)
Urea (mmol/L)				
WS	35.88±0.94 (24.06–51.41)	32.09±1.45 (22.84–47.12)	39.06±3.49 (26.41–80.68)	46.86±15.41 (16.42–84.61)
SA	24.33±1.54 (9.56–64.2)	35.27±6.32 (19.32–64.97)	29.43±1.76 (26.41–33.77)	45.22±8.04 (28.13–89.96)
Creatinine (μmol/L)				
WS	86.63±1.66 (64.53–111.38)	89.33±2.88 (68.95–114.92)	88.52±2.68 (73.37–110.5)	92.11±11.26 (68.06–134.36)
SA	108.76±3.51 (69.31–152.93)	116.68±6.87 (99.89–143.21)	87.95±3.33 (83.09–97.24)	78.04±5.98 (53.04–99.89)
AST (IU/L)				
WS	142.34±4.57 (91–263)	151.21±9.23 (97–263)	147.92±18.21 (106–375)	144.31±73.21 (30–426)
SA	133.81±10.46 (90.4–442)	142.66±13.08 (115–199)	120.92±10.75 (95.8–148)	194.28±41.15 (87–405)
CK (IU/L)				
WS	367.68±42.51 (115–2,110)	451.31±89.50 (137–1,876)	365.85±79.79 (137–1,333)	412.91±67.18 (241–573)
SA	267.52±24.45 (81–816)	356.66±54.89 (200–557)	176.25±35.59 (115–255)	461.57±233.44 (158–1,857)
LDH (IU/L)				
WS	475.29±15.97 (130–716)	585.15±68.08 (345–1,746)	478.64±45.57 (222–990)	473.71±87.33 (294–680)
SA	467.14±35.23 (247–1,227)	488±27.98 (423–600)	395.75±20.71 (342–443)	555.42±95.74 (363–1,099)
Proteins (g/L)				
WS	66.33±11.23 (50.5–80.0)	68.37±17.52 (49.0–86.0)	68.71±17.58 (60.0–81.0)	87.90±45.12 (76.0–98.5)
SA	72.12±14.47 (43.1–90.0)	72.0±29.21 (58.0–78.0)	79.25±29.26 (72.0–86.0)	85.14±18.95 (79.0–93.0)
Albumin (g/L)				
WS	32.97±10.28 (24.3–61.1)	32.76±15.31 (26.6–40.7)	29.11±7.72 (27.4–34.2)	18.8±4.63 (17.0–19.5)
SA	43.48±38.31 (30.3–57.7)	30.93±27.93 (23.0–35.0)	27.93±11.92 (26.5–30.3)	28.5±13.12 (23.5–31.3)

[a] AST = aspartate aminotransferase; CK = creatine kinase; LDH = lactate dehydrogenase.

TABLE 4. Model selection for the seasonal effects of sarcoptic mange on body weight and hematologic and serum biochemical parameters in 136 male Iberian ibex, *Capra pyrenaica*, >3 yr old, captured in the Sierra Nevada Natural Space, southern Spain, 2005–12. Models with substantial support are in bold.[a]

Biological models	K	AICc	Δi	*W*i
Body weight				
**Mange status*Period of the year**	9	1,008.31	0	0.52
**Mange status+Period of the year**	6	1,008.47	0.17	0.47
Mange status	5	1,028.80	20.49	<0.01
Period of the year	3	1,054.21	45.90	<0.01
Mo	2	1,066.92	58.61	<0.01
Hematologic parameters				
**Mange status*Period of the year**	9	10,354.79	0	1
Mange status+Period of the year	6	10,436.41	80.84	0
Mange status	5	10,439.68	83.92	0
Mo	2	10,847.27	491.14	0
Period of the year	3	10,848.08	492.04	0
Serum-biochemical parameters				
**Mange status*Period of the year**	9	9,341.04	0	1
Mange status+Period of the year	6	9,378.06	37.01	0
Mange status	5	9,405.88	64.83	0
Period of the year	3	9,419.90	78.86	0
Mo	2	9,446.14	105.10	0

[a] K = number of parameters; AICc = Akaike Information Criterion corrected for small samples sizes; Δi = difference of AICc with respect to the best model; *W*i = Akaike weight; Mo = null model.

represent a challenge in terms of energy ($\beta_{\text{Winter–Spring}}=-8.46$, SE=1.7, $t=-4.85$), with both infested and healthy ibexes between 2 and 11 kg, respectively, heavier in the warm season (see Table 1).

The effects of mange on both hematologic and serum biochemical parameters depended on the period of the year ($w_{\text{Mange status*Period of the year}}=1$ for both parameters; Table 4). This model explained between 97.8% ($F_{7,128}=848.4$) to 82.6% ($F_{7,128}=51.44$) of the observed hematologic and serum biochemical changes of ibexes, respectively. Nevertheless, all hematologic parameters were not affected by mange or seasons in the same way (Tables 5, 6). The single effect of mange influenced RBC, Hb, hematocrit, MCV, leukocytes, band neutrophils, and monocytes, but MCH, MCHC, and neutrophils were affected by both mange and the period of the year (Table 5). Similarly, cholesterol, urea, and AST were solely influenced by the severity of mange infection, whereas glucose and proteins were also influenced by the period of the year. The interaction of effects of mange and the period of the year influenced values of creatine, LDH and albumin (Table 6), and CK was exclusively affected by the period of the year.

DISCUSSION

Ibexes in our study were chemically immobilized in a way similar to that described elsewhere (Casas-Díaz et al. 2011), so confounding effects due to different capture methods were not expected. Anemia detected by reduced RBC, Hb, and hematocrit in scabietic ibexes was in agreement with previous studies (Arlian et al. 1988; Kido et al. 2011). Anemia in scabietic ibexes might be due to the inflammatory process associated with mange (Pence and Ueckermann 2002). In response to inflammatory cytokines, iron may be sequestered in bone marrow macrophages as a protective mechanism to deny this key element to potential pathogens that require it for

TABLE 5. Analysis of variance table showing those hematologic parameters influenced by the period of year, mange infection, or their interaction in 136 male Iberian ibex, *Capra pyrenaica*, >3 yr old, captured in the Sierra Nevada Natural Space, southern Spain, 2005–12. For both eosinophils and lymphocytes, neither mange infection, period of year, nor their interaction were statistically significant.

Parameter[a]	Mean sum of squares	*F*	*P*
RBC			
Mange status	35.59	12.92	<0.001
Hemoglobin			
Mange status	5,214.21	13.61	<0.001
Hematocrit			
Mange status	99.08	7.41	<0.001
MCH			
Mange status*Period of the year	1.39	3.22	0.02
MCV			
Mange status	28.16	4.88	<0.001
MCHC			
Mange status*Period of the year	5,214.21	13.61	<0.001
Leukocytes			
Mange status	0.32	13.71	<0.001
Bands			
Mange status	2.28	11.72	<0.001
Neutrophils			
Mange status*Period of the year	0.10	2.88	0.03
Monocytes			
Mange status	1.97	5.28	0.001

[a] RBC = red blood cells; MCH = mean corpuscular hemoglobin; MCV = mean corpuscular volume; MCHC = mean corpuscular hemoglobin concentration.

growth and multiplication (Carlson 1990; Weiss and Goodnough 2005). Iron-related measurements were not carried out in our study. Iron may also have a nutritional origin due to combined protein, vitamin, and mineral deficiencies (Watson and Canfield 2000). Neutrophils were higher in ibexes affected by sarcoptic mange than in clinically normal animals, in agreement with previous studies on infected mammals (Kido et al. 2011). Season-related neutrophilia has been reported in wild ungulates (Cross et al. 1994). In winter, the additive effect of season and disease could lead to a rise in neutrophils and, therefore, in total leukocytes.

Ibexes showing more extensive and chronic lesions (category 3) showed the lowest values of blood eosinophils throughout the year. The number of eosinophils in the dermis was positively correlated with the density of mites (Skerrat 2003); the fact that mites decrease in lesions as the hypersensitivity reaction progresses (Arlian 1996) may explain our finding. However, we did not count mites.

The increased blood urea, particularly in winter-spring, coupled with a decrease in creatine concentration suggest an accelerating net catabolism of body protein storage consistent with the progressive weight loss previously reported in scabietic Alpine chamois (Tataruch et al. 1985). Scabietic animals had higher values of total serum proteins than nonaffected animals, which is expected in chronic infections (Jain 1993; Lastras et al. 2000). Values of

TABLE 6. Analysis of variance table showing those serum biochemical parameters influenced by period of the year, mange infection, or their interaction in 136 male Iberian ibex, *Capra pyrenaica*, >3 yr old, captured in the Sierra Nevada Natural Space, southern Spain, 2005–12.

Parameter[a]	Mean sum of squares	F	*P*
Glucose			
Mange status	15.09	3.2	0.02
Period of the year	28.66	6.18	0.02
Cholesterol			
Mange status	0.19	3.99	0.01
Urea			
Mange status	507.20	3.79	0.01
Creatinine			
Mange status*Period of the year	597.12	3.31	0.02
AST			
Mange status	28.16	4.88	<0.001
CK			
Period of the year	177,796.50	4.58	0.03
LDH			
Mange status*Period of the year	69,493	3.36	0.02
Proteins			
Mange status	103,139	16.53	<0.001
Period of the year	60,057	9.62	0.002
Albumin			
Mange status*Period of the year	16,369	3.81	0.01

[a] AST = aspartate aminotransferase; CK = creatine kinase; LDH = lactate dehydrogenase.

other analytes such as AST, CK, and LDH differ and agree with those described in southern chamois and Iberian ibex (López-Olvera et al. 2006; Casas-Díaz et al. 2008).

In conclusion, sarcoptic mange produced a marked reduction of body weight in infested ibexes with a clear seasonal trend. Scabietic animals exhibited anemia and poor nutritional condition. Most of the hematologic parameters studied were related to mange status and leukograms, suggesting secondary infection was involved. Some of the biochemical parameters sampled depended on both the disease stage and the period of the year (e.g., glucose, creatine, LDH, proteins, and albumin); others (e.g., cholesterol, urea, and AST), depended exclusively on mange status; finally, CK depended solely on the period of the year.

Continued studies of how food shortages, due to changes in feeding behavior, primary productivity, or overpopulation of wild and domestic herbivores, influence the response of ibexes to mange infection will provide new strategies and opportunities for controlling this disease in the wild.

ACKNOWLEDGMENTS

Thanks to the staff of the Sierra Nevada Natural Space for their help. Our study was funded by the Ministerio de Economía y Competitividad of the Spanish Government (project CGL2012-40043-C02-01). The authors' research activities are partially funded by the Plan Andaluz de Investigación (RNM-118 group), and E.S. was supported by the postdoctoral program (SFRH/BPD/96637/2013) of the Fundação para a Ciência e a Tecnologia, Portugal. This study complied with current Andalusian and Spanish laws.

LITERATURE CITED

Akomas SC, Obijuru OC, Herbert U. 2011. Hematologic and serologic changes following ivermec-

tin treatment in mange infested West African dwarf goats. *Adv Environ Biol* 5:2557–2560.

Arlian LG. 1996. Immunology of scabies. In: *The immunology of host-ectoparasitic arthropod relationships*, Wikel SK, editor. CAB International, Wallingford, UK, pp. 232–258.

Arlian LG, Ahmed M, Vyszenski-Moher DL. 1988. Effects of *S. scabiei* var. *canis* (Acari: Sarcoptidae) on blood indexes of parasitized rabbits. *J Med Entomol* 25:360–369.

Burnham KP, Anderson DR. 2002. *Model selection and multimodel inference: A practical information-theoretic approach*. Springer-Verlag, New York, New York, 488 pp.

Carlson GP. 1990. Diseases of the hematopoietic and hemolymphatic systems. In: *Large animal internal medicine*, Smith BP, editor. C. V. Mosby Company, Maryland Heights, Missouri, pp. 1068–1126.

Casas-Díaz E, López-Olvera JR, Mentaberre G, Marco I, Lavín S. 2008. Hematologic and biochemical values for Spanish ibex (*Capra pyrenaica*) captured via drive-net and box-trap. *J Wildl Dis* 44:965–972.

Casas-Díaz E, Marco I, López-Olvera JR, Mentaberre G, Lavín S. 2011. Comparison of xylazine-ketamine and medetomidine-ketamine anaesthesia in the Iberian ibex (*Capra pyrenaica*). *Eur J Wildl Res* 57:887–893.

Cross JP, Mackintosh CG, Griffin JFT. 1994. Haematological reference values for farmed red deer (*Cervus elaphus*) in New Zealand. *Comp Haematol Int* 4:76–85.

Fandos P. 1991. *La cabra montés* (Capra pyrenaica) *en el Parque Natural de las Sierras de Cazorla, Segura y Las Villas*. ICONA-CSIC, Madrid, Spain, 176 pp.

Jain NC. 1993. *Essentials of veterinary hematology*. Lea and Febiger, Philadelphia, Pennsylvania, 417 pp.

Kido N, Kamegaya C, Omiya T, Wada Y, Takahashi M, Yamamoto Y. 2011. Hematology and serum biochemistry in debilitated, free-ranging raccoon dogs (*Nyctereutes procyonoides*) infested with sarcoptic mange. *Parasitol Int* 60:425–428.

Lastras ME, Pastor J, Marco I, Ruiz M, Viñas L, Lavin S. 2000. Effects of sarcoptic mange on serum proteins and immunoglobulin G levels in chamois (*Rupicapra pyrenaica*) and Spanish ibex (*Capra pyrenaica*). *Vet Parasitol* 88:313–319.

López-Olvera JR, Marco I, Montané J, Lavín S. 2006. Haematological and serum biochemical values of southern chamois (*Rupicapra pyrenaica*). *Vet Rec* 158:479–484.

Peinado M, Rivas-Martínez S, editors. 1987. *La vegetación de España*. Servicio de Publicaciones de la Universidad de Alcalá de Henares, Alcalá de Henares, Spain, 744 pp.

Pence DB, Ueckermann E. 2002. Sarcoptic mange in wildlife. *Rev Sci Tech OIE* 21:385–398.

Pérez JM, Granados JE, Sarasa M, Serrano E. 2011. Usefulness of estimated surface area of damaged skin as a proxy of mite load in the monitoring of sarcoptic mange in free-ranging populations of Iberian wild goat, *Capra pyrenaica*. *Vet Parasitol* 176:258–264.

R Development Core Team. 2014. *R: A language and environment for statistical computing*. R Foundation for Statistical Computing, Vienna, Austria, http://www.R-project.org. Accessed January 2014.

Sarasa M, Rambozzi L, Rossi L, Meneguz PG, Serrano E, Granados JE, González FJ, Fandos P, Soriguer RC, Gonzalez G, et al. 2010. *Sarcoptes scabiei*: Specific immune response to sarcoptic mange in the Iberian ibex *Capra pyrenaica* depends on previous exposure and sex. *Exp Parasitol* 124:265–271.

Serrano E, Gállego L, Pérez JM. 2004. Ossification of the appendicular skeleton in the Spanish ibex *Capra pyrenaica* Schinz, 1838 (Artiodactyla: Bovidae), with regard to determination of age. *Anat Histol Embryol* 33:33–37.

Serrano E, González FJ, Granados JE, Fandos P, Soriguer RC, Pérez JM. 2008. The use of total serum proteins and triglycerides for monitoring body condition in the Iberian wild goat (*Capra pyrenaica*). *J Zoo Wildl Med* 39:646–649.

Serrano E, Granados JE, Sarasa M, González FJ, Fandos P, Soriguer RC, Pérez JM. 2011. The effects of winter severity and population density on body stores in the Iberian wild goat (*Capra pyrenaica*) in a highly seasonal mountain environment. *Eur J Wildl Res* 57:45–55.

Skerrat LF. 2003. Cellular response in the dermis of common wombats infected with *Sarcoptes scabiei* var. *wombati*. *J Wildl Dis* 39:93–202.

Tataruch F, Steineck T, Onderscheka K. 1985. Investigations on the metabolism of chamois suffering from sarcoptic mange. In: *The biology and management of mountain ungulates*, Lovari S, editor. Croom Helm, London, UK, pp. 250–255.

Watson ADJ, Canfield PJ. 2000. Nutritional deficiency anaemias. In: *Schalm's veterinary hematology*, 5th Ed., Feldman BF, Zinkl JG, Jain NC, editors. Lippincott Williams & Wilkins, Baltimore, Maryland, pp. 190–195.

Weiss G, Goodnough LT. 2005. Anemia of chronic disease. *N Engl J Med* 352:1011–1123.

Submitted for publication 13 September 2013.
Accepted 6 June 2014.
